# G-cleave LC3B biosensor: monitoring autophagy and assessing resveratrol's synergistic impact on doxorubicin-induced apoptosis in breast cancer cells

**DOI:** 10.1186/s13058-024-01951-1

**Published:** 2024-12-30

**Authors:** Chiao-Chun Liao, Yuqing Long, Ming-Lin Tsai, Chun-Yu Lin, Kai-Wen Hsu, Chia-Hwa Lee

**Affiliations:** 1 Department of Tropical Medicine, School of Medicine, College of Medicine, National Yang Ming Chiao Tung University, Taipei, Taiwan; 2 School of Medical Laboratory Science and Biotechnology, College of Medical Science and Technology, Taipei Medical University, New Taipei City, Taiwan; 3 Respiratory Medicine Unit and National Institute for Health Research (NIHR) Oxford Biomedical Research Centre (BRC), Nuffield Department of Medicine Experimental Medicine, University of Oxford, Oxford, UK; 4 Chinese Academy of Medical Sciences Oxford Institute (COI), University of Oxford, Oxford, UK; 5 Department of General Surgery, Cathay General Hospital, Taipei, Taiwan; 6 Institute of Bioinformatics and Systems Biology, National Yang Ming Chiao Tung University, Hsinchu, Taiwan; 7 Center for Intelligent Drug Systems and Smart Bio-Devices (IDS2B), National Yang Ming Chiao Tung University, Hsinchu, Taiwan; 8 Institute of Translational Medicine and New Drug Development, China Medical University, Taichung City, Taiwan; 9 Research Center for Cancer Biology, China Medical University, Taichung City, Taiwan; 10 TMU Research Center of Cancer Translational Medicine, Taipei Medical University, Taipei, Taiwan; 11 Ph.D. Program in Medical Biotechnology, College of Medical Science and Technology, Taipei Medical University, New Taipei City, Taiwan

**Keywords:** Autophagy, Non-invasive autophagy detection system (NIADS), G-cleave LC3B biosensor, Resveratrol, Doxorubicin, Breast cancer

## Abstract

Autophagy, a crucial process in cancer, is closely intertwined with both tumor progression and drug resistance development. However, existing methods used to assess autophagy activity often pose invasiveness and time-related constraints, limiting their applicability in preclinical drug investigations. In this study, we developed a non-invasive autophagy detection system (NIADS-autophagy, also called G-cleave LC3B biosensor) by integrating a split-luciferase-based biosensor with an LC3B cleavage sequence, which swiftly identified classic autophagic triggers, such as Earle's Balanced Salt Solution and serum deprivation, through protease-mediated degradation pathways. The specificity of G-cleave LC3B biosensor was confirmed via CRISPR gene editing of pivotal autophagy regulator ATG4B, yielding diminished luciferase activity in MDA-MB-231 breast cancer cells. Notably, the G-cleave LC3B biosensor exhibited strong concordance with established autophagy metrics, encompassing LC3B lipidation, SQSTM1 degradation, and puncta accumulation analysis. To underscore the usage potential of the G-cleave LC3B biosensor, we discovered that resveratrol acts as a synergistic enhancer by significantly potentiating apoptosis in MDA-MB-231 cells when combined with doxorubicin treatment. Overall, the luminescence-based G-cleave LC3B biosensor presents a rapid and dependable avenue for determining autophagy activity, thereby facilitating high-throughput assessment of promising autophagy-associated anti-cancer therapies across diverse malignancies.

**Supplementary Information:**

The online version contains supplementary material available at 10.1186/s13058-024-01951-1.

## Introduction

Autophagy, an evolutionarily conserved cellular process, plays a pivotal role in maintaining cellular homeostasis and viability by degrading cytosolic components, including damaged organelles, misfolded proteins, and dysfunctional molecules [1]. The intricate relationship between autophagy and cancer has garnered extensive research attention, revealing the dichotomous impact of autophagy acting as a tumor suppressor and promoter, contingent upon the complex interplay within the cancer microenvironment and developmental stage. Cellular stressors, notably nutrient deprivation and stimuli such as Earle's Balanced Salt Solution (EBSS) or serum starvation, are frequently employed as triggers to invoke canonical autophagy. Once activated, autophagy-related (ATG) genes orchestrate the formation of autophagosomes, double-membrane structures that merge with lysosomes, thereby enabling degradation and energy replenishment [2]. A critical juncture in autophagosome assembly involves the conjugation of LC3B to produce the lipid-conjugated LC3-II species, serving as a pivotal marker for determining autophagy [3]. In addition, the ubiquitin-proteasome system (UPS) is a primary mechanism interplay with autophagy for intracellular protein degradation. While UPS efficiently manages short-lived proteins, autophagy targets long-lived proteins and organelles for degradation via lysosomal pathways. These systems coordinated especially under conditions of cellular stress, where autophagy can compensate for UPS dysfunction, particularly when it becomes overwhelmed by protein aggregates, providing a complementary approach in maintaining proteostasis [4, 5]. Selective cargo degradation mediated by autophagy receptor SQSTM1 entails ubiquitinated substrates binding, enclosing them within autophagosomes, and subsequent degradation upon fusion with lysosomes [6]. The multifunctional nature of autophagy in various cellular contexts makes it a complex regulator of tumor development. Therefore, understanding the role of autophagy in cancer progression is essential for developing effective therapeutic strategies.

Autophagy is an essential process that maintains cellular homeostasis and is also involved in regulating tumorigenesis. While the tumor-suppressive mechanism of autophagy can disrupt the initial occurrence of cancer cells in the early stages of tumorigenesis, the protective function of autophagy can promote the survival, metastasis, and drug resistance of cancer cells during late-stage tumorigenesis [7]. Recent investigations have highlighted the involvements of autophagy in drug resistance and tumor relapse in breast cancer. Notably, specific chemotherapy-resistant breast cancer cells exhibit elevated autophagic activity, and inhibiting this process can restore their sensitivity to chemotherapy [8, 9]. Conversely, hyperactivation of autophagy can also lead to cell death and enhance susceptibility to anti-cancer therapies, particularly in scenarios involving apoptosis-resistant therapies [10, 11]. Thus, developing an effective and sensitive autophagy sensor system in a well-studied breast cancer model is critical for the high-throughput screening of autophagy target drugs and modulating the effects of anti-cancer drugs on autophagy-related tumorigenesis.

Breast cancer is characterized as one of the most aggressive malignancies, with the highest incidence rate among women worldwide. The tumorigenesis and treatment model of breast cancer has been extensively studied over the years [12–14]. Recently, clinical trials have explored using autophagy-targeting drugs, alone or in combination with standard therapies, in breast cancer treatment, some autophagy-targeting drugs used in combination with standard therapies include autophagy activators or inhibitors such as Everolimus, Rapamycin, chloroquine, Hydroxychloroquine, Bafilomycin A1 [10]. Studies have demonstrated the association and relationship between autophagy and apoptosis with cell growth, metastasis, and chemo-resistance in the tumor microenvironment on breast cancers. In recent discoveries, autophagy in breast cancer can either protect tumor cells from cell death [15] or enhance the efficacy of cancer therapies [16], depending on the specific cellular context and treatment strategy. As a result, developing novel strategies that target autophagy in breast cancer holds potential to enhance therapeutic efficacy and improve patient outcomes.

In recent years, there has been increasing interest in developing robust methods to evaluate autophagy dynamics and identify autophagy-modulating agents for cancer therapy. Although some approaches have been reported to directly or indirectly quantify autophagic flux by monitoring ratio changes using autophagy inducers and inhibitors, such as GFP/RFP [17] or pHluorin/mKate [18], many existing methods reflect distinct autophagy steps without quantifying the complete process. These methods primarily assess autophagy accumulation and subsequent biological effects through inhibitor treatments [19]. Furthermore, many of these measurements are fluorescence-based and conducted long after the initial perturbations, which limits their effectiveness in real-time assessment of autophagic flux activation or inhibition in live cancer cells.

Autophagy plays a dual role, and it can be a target to induce apoptosis and a mechanism to enhance immune responses against breast cancer, as demonstrated in previous studies [20, 21]. The manipulation of autophagy, whether through gene silencing [22], miRNA delivery [21, 23], or photothermal therapy [22], holds promise for improving breast cancer treatment outcomes. Due to the complex and multifaceted nature of autophagy in breast cancer treatment, there is an urgent need to develop efficient and effective therapies targeting this process. This study addressed this challenge by introducing a novel bioluminescence-based detection system, the G-cleave LC3B biosensor. The cleavage of LC3 by ATG4 at the Gly residue to expose the COOH group is a key initiating step in LC3 priming, which is followed by subsequent LC3 lipidation or delipidation during the LC3-PE conjugation process [24]. This system utilizes a critical LC3B substrate peptide specific to ATG4B cleavage to monitor autophagy activation through luminescence degradation in highly aggressive and well-characterized triple-negative breast cancer (TNBC). Our innovative autophagy biosensor provides valuable insights into the complexities of autophagy and heralds a new era for high-throughput screening of autophagy-regulating drugs, which hold the potential to improve anti-cancer efficacy in clinical cancer treatments.

## Material and methods

### Cell culture

The human mammary gland epithelial cancer lines MDA-MB-231, MDA-MB-453 and MDA-MB-468 were obtained from the American Type Culture Collection (ATCC). The above cells expressing *pEGFP-LC3B*^*pepABLuc*^ and ATG4B gene-edited cells were cultured in DMEM/F12 medium supplemented with 10% fetal bovine serum (FBS), 100 units/mL penicillin, and 100 µg/mL streptomycin in a humidified atmosphere containing 5% CO_2_ at 37 °C.

### Plasmid construction of *pEGFP-LC3B*^*pepABLuc*^

The plasmid of *pEGFP-LC3B*^*pepABLuc*^ is modified from our previous non-invasive apoptosis detection sensor (NIADS) system [25–28]. In brief, pepA and pepB nucleotide sequences were linked with N’ and C’ split luciferase, respectively. The fragments of pepA-N-luc and pepB-C-luc were constructed in PLAS3.1-neo lentivirus plasmid with 3X LC3 cleavage sequences (TFGMKLSV) in between [29]. The LC3B^*pepABLuc*^ sequence was linked with the EGFP sequence, separated by P2A and T2A autocleavage sequences. The whole *pEGFP-LC3B*^*pepABLuc*^ sequence was confirmed by Sanger sequencing.

### Autophagy flux observing by *mCherry-EGFP-LC3B* puncta formation

MDA-MB-231 cells expressing *pmCherry-EGFP-LC3B* were generated by lentivirus infection system, as previously described [25, 28, 30, 31]. After appropriate treatments, fluorescent signals of mCherry (red), EGFP (green), and mCherry/EGFP merge (yellow/orange) puncta from MDA-MB-231 cells expressing *pmCherry-EGFP-LC3B* were visualized using a fluorescent microscope. The increase in autophagy flux was assessed by examining the levels of EGFP, mCherry, and mCherry/EGFP puncta with or without autophagy degradation inhibition. The appearance of increased mCherry/EGFP merge puncta (autophagolysosomes) in the presence of the degradation inhibitor chloroquine (CQ) indicated the activation of complete autophagy flux. Fluorescent images were captured using a Zeiss LSM900 Inverted Laser Scanning Confocal Microscope with Airyscan (Carl Zeiss AG, Germany). The fluorescence puncta per cell were quantified by analyzing 20 to 30 cells per experiment using MetaMorph software (Molecular Devices, Inc.).

### Gene editing lentivirus packing and transfection

The production of the fusion protein from the *pEGFP-LC3B*^*pepABLuc*^ construct and the CRISPR/CAS9 gene editing of the ATG4B gene were carried out using the lentivirus infection system, as previously described [25, 28, 30, 31]. In brief, phoenix cells were co-transfected with the plasmid of *pEGFP-LC3B*^*pepABLuc*^ (G-cleaved LC3B biosensor) or lentiCRISPR v2 targeting ATG4B along with pMD2.G (Addgene plasmid #12259) and psPAX2 (Addgene plasmid #12260, both kindly provided by Didier Trono, EPFL, Lausanne, Switzerland), using the TransIT^®^-LT1 transfection reagent for 3 days. The medium containing lentiviral particles was collected and concentrated using Lenti-X Concentrator^®^ (Clontech, Mountain View, CA, USA). The concentration of lentivirus production was evaluated by qPCR before infecting MDA-MB-231 cells. Antibiotics of neomycin (6 µg/ml) or puromycin (2.5 µg/ml) were added 2 days after infection. MDA-MB-231 cells expressing *pEGFP-LC3B*^*pepABLuc*^ (G-cleaved LC3B biosensor) were analyzed with gene expression of EGFP and luciferase using Microscope and Immunoblotting. The gene editing efficiency and the expression level of the G-cleaved LC3B biosensor infected with or without lentiCRISPR v2 targeting ATG4B were evaluated using Sanger sequencing and Immunoblotting.

### Immunoblot analysis

Protein extraction from cell lysates of MDA-MB-231, MDA-MB-453 and MDA-MB-468 cells expressing *pEGFP-LC3B*^*pepABLuc*^ following appropriate treatments was conducted using radioimmunoprecipitation assay (RIPA) lysis buffer. RIPA lysis buffer recipe includes following chemicals, 140 mM NaCl, 20 mM Tris-HCl pH7.9, 10 mM NaF, 5 mM EDTA, 1 mM EGTA, 1%Triton X-100, 1 mM sodium orthovanadate, 1 mM sodium pyrophosphate, 100 μM β-glycerophosphate and 1 mM PMSF. Equal concentrations of protein extracts were denatured at 95 °C for 5 min and loaded onto sodium dodecyl sulfate polyacrylamide gel electrophoresis (SDS-PAGE) with SDS loading buffer. The resolved proteins in SDS-PAGE were then transferred onto polyvinylidene difluoride (PVDF) membranes, which were blocked with 5% skim milk, hybridized with specific primary and corresponding secondary antibodies with horseradish peroxidase conjugation. TBST (20 mM Tris-base, 0.15 M NaCl, 0.1% Tween 20) buffer was used for all antibody dilution in this study. The anti-GAPDH (sc-32233) was purchased from Santa Cruz Biotechnology (Santa Cruz, CA, USA); the anti-EGFP (GTX113617), anti-Luciferase (GTX125850) and anti-caspase 3 (GTX110543) antibodies were purchased from GeneTex Inc. (Irvine, CA, USA); and the anti-β-actin (A5441) was purchased from Sigma-Aldrich (St. Louis, MO, USA); and the anti-ATG4B (M134-3MS) was purchased from Medical & Biological Laboratories co. (Tokyo, Japan); and the anti-SQSTM1 (H00008878-M01) antibody was purchased from Novus Biologicals, LLC (Colorado, USA); and anti-LC3B (#3868), anti-PARP (#9532) antibodies were purchased from Cell Signaling Technology (Danvers, MA, USA). The secondary anti-mouse and anti-rabbit antibodies were purchased from Santa Cruz Biotechnology. All primary antibodies were used at a 1:1000 dilution with overnight hybridization, followed by one-hour incubation with a 1:5000 dilution of the secondary antibodies. All western blot assays in TNBC cell lines were performed in at least three independent experiments. Protein immunoreactivity was captured using an ImageQuant LAS 500 or GE Amersham Imager-600/680 chemiluminescence CCD camera. The quantification of protein immunoreactivity for all blots was performed using densitometric analysis with ImageJ software (1.53 k, NIH Image, Bethesda, MD, USA), whereas the protein intensities were normalized by their internal control of either β-actin or GAPDH. Detailed results and figures are provided in Supplementary Figs. 8, 9, 10, 11 and 12, along with additional information in the Supplementary section.

### Bio-luminescence detections of luciferase degradation activity

We utilized both bioluminescence imaging (IVIS) and a cellular bioluminescence assay to monitor the degradative luciferase activity of the G-cleave LC3B biosensor, as previously described [26]. Briefly, MDA-MB-231 cells expressing *pEGFP-LC3B*^*pepABLuc*^ were seeded in 24-well or 96-well plates, treated with drugs or nutrient depletion for 4 h, and then incubated with 1.5 mg/mL D-luciferin in PBS. Bioluminescence was detected immediately and quantified either by imaging photon counts or by measuring the luminescence signal with a Molecular SpectraMax ID3 or Biotek Synergy HT Multi-detection microplate reader. Images of luminescence photon counts were captured by the Xenogen IVIS CCD camera and analyzed by Living Image^®^ software 4.0, while the total photon flux was measured using the luminescence microplate reader. The luciferase degradation activity was quantified as the relative reduction fold in cellular luminescence subsequent to treatment, normalized against the corresponding luminescence in the control group. All bioluminescent assays were conducted in triplicate independent experiments. The detailed quantification of these results can be found in the Supplementary information file.

### Autophagy activity calculation

The luciferase degradation fold change was calculated as the ratio of the decline in cellular luminescence following treatment compared to the control. The reciprocal of the luciferase degradation fold change was then used to represent autophagy activity, as indicated in the figures. This process involves measuring luminescence and EGFP values using a microplate reader and then normalizing each luminescence value against its corresponding EGFP value. The autophagy activity is then calculated as follows:

$${\text{Luminescence normalization}} = \frac{{{\text{Luminescence}}}}{{{\text{EGFP}}}}$$

$${\text{Autophagy }}\;{\text{activity}} = \frac{1}{{{\text{Average}}\;{\text{ treatment/Average }}\;{\text{control}}}}$$

This method accurately interprets the decrease in luminescence as an indicator of autophagy activity specific to the G-cleave LC3B biosensor. The calculating results were provided in the supplementary information.

### Cell viability assay

Cell survival and cytotoxicity were evaluated using the WST-8/CCK8 cell counting reagent according to the manufacturer's instructions. Specifically, MDA-MB-231 cells were seeded onto 96-well plates and treated with DOX (HY-15142, MedChem Express, NJ, USA) and RSV (HY-16561, MedChem Express, NJ, USA) alone or in combination, and incubated at 37 °C with 5% CO_2_. After the indicated treatment times, the medium was removed, and 10 μl of WST-8/CCK8 solution in 100 μl of culture medium was added to the wells and incubated for 2 h. The optical density (O.D.) values at 450 nm and 650 nm were then measured using an Absorbance Microplate Reader. The ratios of surviving cells were calculated by subtracting the 650 nm absorbance value from the 450 nm value to eliminate background noise.

### Sub-G1 apoptosis determination

The apoptotic cells population of sub-G1 phase in the distribution of cell cycle in MDA-MB-231 cells was analyzed by flow cytometry. Briefly, MDA-MB-231 cells were treated with 25–100 μM RSV in combine with 0.5 μM of DOX. After 48 h incubation, suspended and attached cells were collected, fixed with 4% paraformaldehyde, incubated with 40 μg/ml RNAse A for 30 min at 37 ℃, and subsequently stained with 50 μg/ml Propidium Iodide. Representative histograms of sub G1 phase distribution and the percentage of parent population depict apoptosis were analyzed by Beckman CytoFLEX.

### Combination index

The combination index (CI) was used to evaluate the interaction between RSV and DOX in combination treatments, determining whether the effects were synergistic (CI < 1), additive (CI = 1), or antagonistic (CI > 1). IC50 values for RSV (10 to 500 μM) and DOX (0.5 to 25 μM) alone, as well as combination treatments of DOX (0.5, 1, 5, 10, 25 μM) with fixed RSV concentrations (5, 10, 25 μM) and RSV (10, 100, 500 μM) with fixed DOX concentrations (0.5, 1, 2 μM), were determined through cell viability assays in MDA-MB-231 cells. The combination doses that achieved 50% inhibition of cell viability were calculated using SigmaPlot's pharmacology module and presented in a table in Fig. 5C. The CI values for specific combinations, shown in Fig. 5C, were calculated according to the Loewe additivity model [26] and interpreted as follows: CI < 1 indicates synergism, CI = 1 indicates additivity, and CI > 1 indicates antagonism. All calculations and details are available in the supplementary information file.

### Statistical analysis

All statistical results were presented as mean ± SE (Standard Error) as analyzed by the two-tailed student t-test. The statistical comparisons were performed using SigmaPlot software. The evaluated *p* values between groups that expressed as *, *p*≦0.05; **, *p*≦0.01; ***, *p*≦0.001; *****, *p*≦0.0001 were considered statistically significant, and all statistical tests were two-sided.

## Results and discussion

### Establishment of lentivirus-mediated G-cleave LC3B biosensor in MDA-MB-231 breast cancer cells

**Fig. 1:**
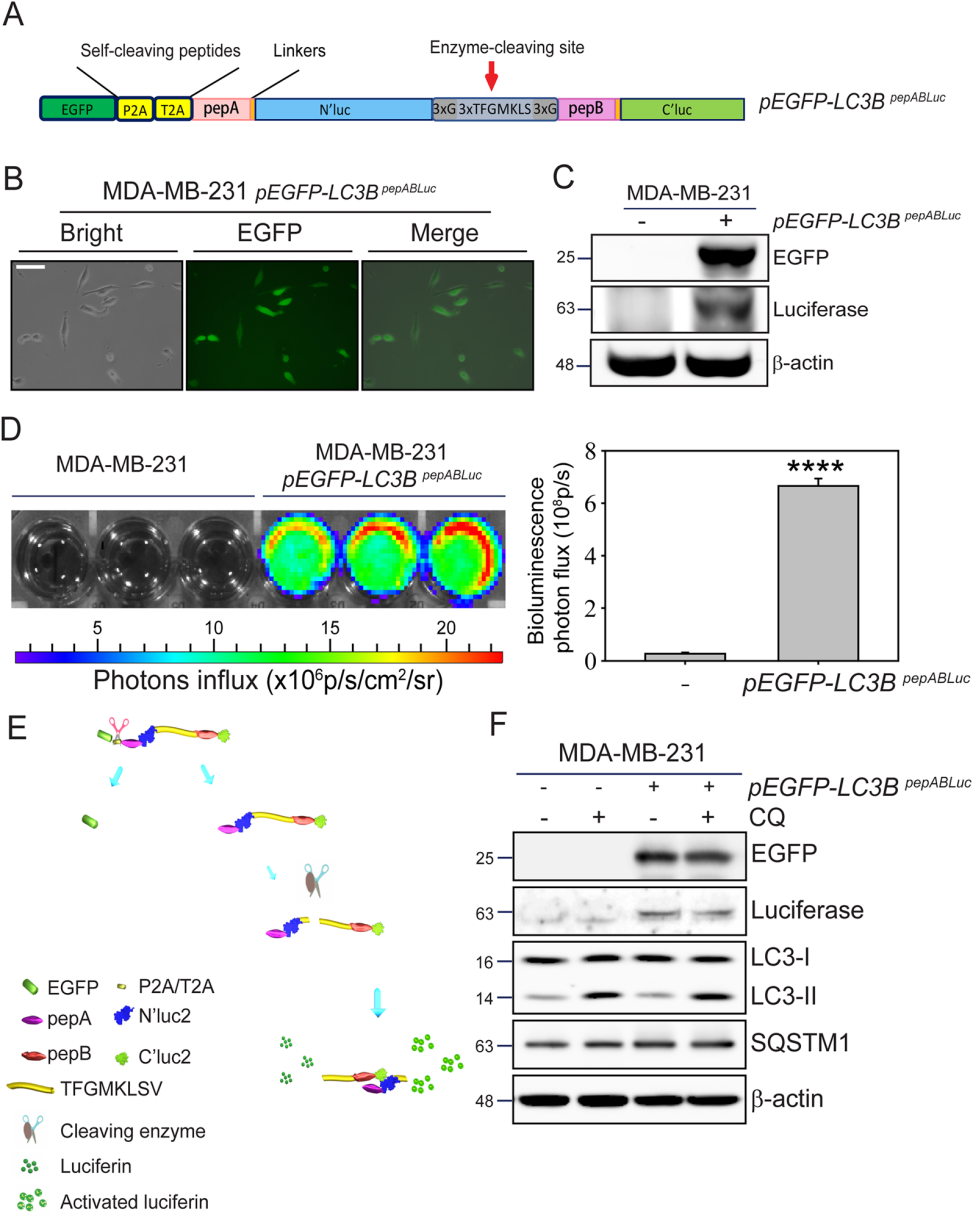
Establishment of G-cleave LC3B biosensor on MDA-MB-231 breast cancer cells. **A** Schematic diagram of the *pEGFP-LC3B*^*pepABLuc*^ construct expressing EGFP- pepABLuc LC3B cleavage peptide. **B** Lentivirus-mediated *pEGFP-LC3B*^*pepABLuc*^ expression in MDA-MB-231 cells was visualized by fluorescence microscope. Scale bar: 50 μm. **C** MDA-MB-231 cells with *pEGFP-LC3B*^*pepABLuc*^ expression were collected, and EGFP and luciferase protein expressions were determined. **D** IVIS images show the luciferase activity of MDA-MB-231 cells with or without lentivirus-mediated expression of *pEGFP-LC3B*^*pepABLuc*^. Quantification of total photons flux was analyzed (n = 3 replicates; student-t test; ****, *p* < 0.0001; bars represent mean ± SE). **E** Schematic representation of the EGFP and luciferase expression of the G-cleave LC3B biosensor. Upon expression of the fusion peptide EGFP-P2A-pepABLC3B in cells, self-cleavage at the P2A/T2A domain releases EGFP from the subsequent pepABLC3B peptide. During autophagy, autophagy signaling cleaves the specific autophagy cleavage sequences (3X TFGMKLSV) and enables the formation of cleaved pepA-N’luc2 and pepB-C’luc2 due to the strong interaction of pepA and pepB fragments. The luciferin addition is, therefore, able to determine autophagy activity (photons influx) through IVIS. **F** Cell lysates from *pEGFP-LC3B*^*pepABLuc*^ expression cells were collected with or without 30 μM CQ. The expressions of EGFP, full-length luciferase, LC3B lipidation, and autophagic degradation were determined by immunoblot. β-actin was used as a loading control

Autophagy is a cellular mechanism with dual roles that regulate tumor initiation, progression, and chemoresistance [32]. It promotes the therapeutic resistance of cancer cells to toxic stress or activates cell death mechanisms to increase treatment efficiency in tumor cells [33]. Several methods and pathways have been reported for detecting autophagy in cancer cells [19]. Still, a responsive and rapid autophagy detection with high-throughput potential among live-cell detection remains to be established. To effectively monitor autophagy induction in cells, we modified the previous NIADS sensor [25, 26, 28] and inserted three repeats of the autophagy cleavage sequence (3X TFGMKLS) between pepA-N’luc and pepB-C’luc fusion proteins, with the repeats of glycine serving as a linker [29, 34]. Next, to utilize this G-cleave LC3B autophagy biosensor (*LC3B*^*pepABLuc*^) for cell normalization, we fused the enhanced green fluorescent protein (EGFP) gene with a self-cleaving peptide P2A/T2A at the amino termini of pepABLC3B (*pEGFP-LC3B*^*pepABLuc*^, Fig. 1A).

After introducing the *pEGFP-LC3B*^*pepABLuc*^ sensor into MDA-MB-231 breast cancer cells via a lentiviral delivery system, strong EGFP signals were observed under fluorescence microscopy (Fig. 1B). The expression of EGFP and luciferase proteins was further validated through immunoblot analysis (Fig. 1C), confirming EGFP as a transfection marker of the *pEGFP-LC3B*^*pepABLuc*^ sensor in MDA-MB-231 cells, alongside the expression of the luciferase fusion protein. To explore the broader applicability of the *pEGFP-LC3B*^*pepABLuc*^ sensor, we also transfected it into MDA-MB-468 and MDA-MB-468 TNBC cells. The expression of both EGFP and luciferase proteins was similarly validated in these cell lines (Supplementary Fig. 1).

We also assessed the enzyme activity of luciferase by adding luciferin as the substrate in MDA-MB-231 cells expressing *pEGFP-LC3B*^*pepABLuc*^. The IVIS detection showed strong and significant luciferase activity in MDA-MB-231 cells transfected with the *pEGFP-LC3B*^*pepABLuc*^ sensor (*p*≦0.0001), compared to the parental MDA-MB-231 cells (Fig. 1D). Based on the above result, we displayed a brief schematic model of how this G-cleave LC3B biosensor functions in MDA-MB-231 cells (Fig. 1E). First, EGFP is released from the pepABLC3B fusion protein via cells self-cleaving at P2A/T2A. Second, autophagy signaling cleaves the specific autophagy cleavage sequences and enables the formation of cleaved (N’luc2 and C’luc2) due to high protein–protein interaction affinity (pepA and pepB). Finally, luciferin exposure activates the luciferase protein, and bioluminescence represents the autophagy LC3B cleavage activity during autophagy events.

It is well known that the LC3B lipidation and autophagy cargo protein (SQSTM1) degradation are commonly used to assess autophagy events [35]. To understand whether the *pEGFP-LC3B*^*pepABLuc*^ sensor might activate autophagy, we measured the LC3B lipidation and cargo protein degradation of *pEGFP-LC3B*^*pepABLuc*^ in MDA-MB-231 and parental cells treated with/without the autophagy degradation inhibitor CQ (Fig. 1F). Immunoblot analysis revealed that LC3-II expression were increased in both MDA-MB-231 (*p*≦0.01) and cells expressing *pEGFP-LC3B*^*pepABLuc*^ (*p*≦0.01) by CQ, while SQSTM1 accumulation occurred in cells expressing *pEGFP-LC3B*^*pepABLuc*^ in the presence (*p*≦0.05) and absence (*p*≦0.05) of CQ, suggesting the expression of *pEGFP-LC3B*^*pepABLuc*^ did not alter the levels of LC3B lipidation or SQSTM1 degradation in MDA-MB-231 cells compared to the parental cells, indicating that this *pEGFP-LC3B*^*pepABLuc*^ biosensor construct does not interfere with endogenous autophagy process. This finding also suggests that the *pEGFP-LC3B*^*pepABLuc*^ (G-cleave LC3B biosensor) in MDA-MB-231 breast cancer cells is ideal for exploring autophagy activation without intruding into endogenous autophagy activity.

### Activation of autophagy in MDA-MB-231 breast cancer cells expressing G-cleave LC3B biosensor

**Fig. 2:**
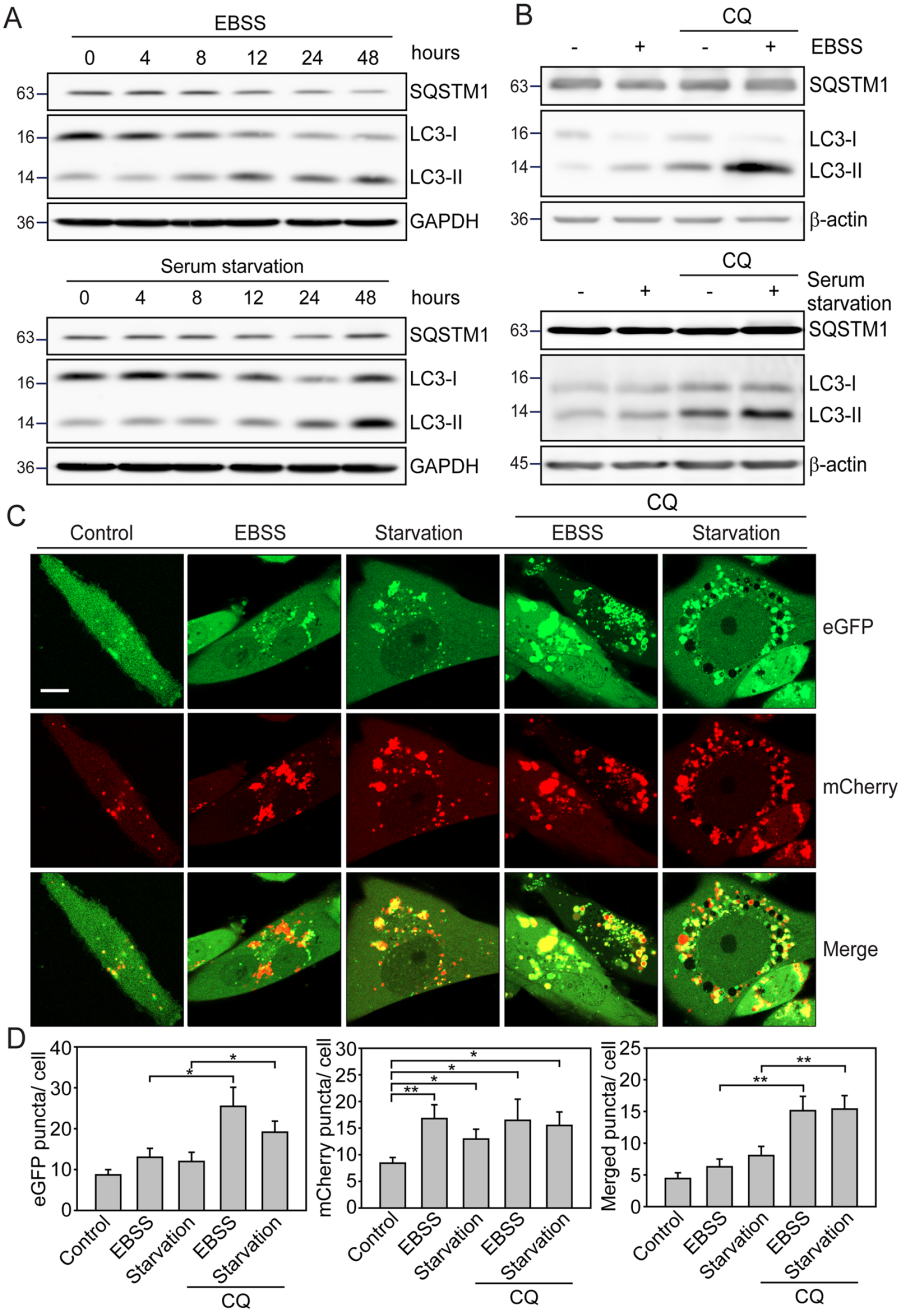
EBSS and serum starvation induce autophagy in MDA-MB-231 cells expressing the G-cleave LC3 biosensor. **A** MDA-MB-231 cells expressing *pEGFP-LC3B*^*pepABLuc*^ were subjected to either Earle balanced salt solution (EBSS) or serum starvation for varying durations as indicated. **B** MDA-MB-231 cells expressing *pEGFP-LC3B*^*pepABLuc*^ were subjected to EBSS or serum starvation in the presence or absence of 30 μM CQ for 24 h. Cell lysates were collected and subjected to immunoblotting analysis to assess the levels of autophagic lipidation (LC3B) and degradation (SQSTM1). GAPDH and β-actin were used as loading controls. **C** MDA-MB-231 cells expressing *pmCherry-EGFP-LC3B* were treated with EBSS or serum starvation medium in the presence or absence of 30 μM CQ for 24 h to determine autophagic flux. The formation of EGFP (green), mCherry (red), and merge (yellow/orange) puncta was observed by the confocal microscopy. Scale bar: 10 μm. **D** Quantitate analysis of mCherry (red), EGFP (green), and merged (yellow/orange) puncta per cell are represented as means ± SEM in 20 to 30 cells in three independent experiments

Autophagy initiation is a multi-step process comprising nucleation, elongation, fusion, and degradation. To elucidate the duration and efficiency of autophagy detection using the G-cleave LC3B biosensor, we evaluated the activation of autophagy in MDA-MB-231 cells expressing the biosensor by nutrient depletion (Fig. 2A). The levels of LC3B lipidation and SQSTM1 degradation were evaluated under EBSS (Fig. 2A, upper panel) or serum starvation (Fig. 2A, lower panel) conditions, both of which led to a time-dependent increase (*p*≦0.05 and *p*≦0.001) in LC3B-II (lipidated LC3B) expression. Correspondingly, the time-dependent degradation of SQSTM1 occurred, with notable compensation in autophagic degradation 48 h after serum starvation, indicating intensified cycles of autophagic degradation. To ensure that the observed increase in LC3B-II was not due to inhibition of autophagy degradation, we co-treated the cells with EBSS/nutrient depletion and CQ. Upon adding CQ, LC3B lipidation induced by either EBSS (Fig. 2B, upper panel) or serum starvation (Fig. 2B, lower panel) was further enhanced (*p*≦0.05 and *p*≦0.01), while the EBSS-mediated SQSTM1 degradation was slightly inhibited (*p*≦0.05). These findings suggest that EBSS/nutrient depletion strategies can be effectively used to explore the G-cleave LC3B biosensor in MDA-MB-231 cells.

To monitor the autophagic flux by visualizing the autophagosomes fusion with lysosomes to form autophagolysosomes, we observed the fluorescent punctate signals in MDA-MB-231 cells transduced with mCherry-EGFP-LC3B. The formation of mCherry (red) and EGFP (green) puncta was visualized in the structure of autophagosomes, shown as yellow/orange puncta in the merged images. Due to the pH sensitivity of EGFP fluorescence (autophagosome formation), which is quenched in the acidic environment of autophagolysosomes, red puncta appear in merged images, representing the lysosome-autophagosome fusion stage. Fluorescence microscopy showed that nutrient depletion increased the number of green puncta, indicating autophagosome formation (Fig. 2C). This was followed by an increase in red puncta, representing autophagosome-lysosome fusion, after EBSS or serum starvation treatment. Furthermore, yellow/orange puncta accumulated, particularly in cells treated with CQ, compared to controls (*p*≦0.05, Fig. 2D), indicating that EBSS/nutrient depletion effectively activated autophagic flux in MDA-MB-231 cells. These results demonstrate that nutrient depletion enhances LC3B lipidation, SQSTM1 degradation, and autophagic flux, all of which can be observed in MDA-MB-231 cells or cells expressing the G-cleave LC3B biosensor using traditional immunoblotting and fluorescence-based autophagy assays.

### EBSS/nutrient depletion stimulates luciferase activity of G-cleave LC3B biosensor in a proteasome degradation-dependent manner

**Fig. 3:**
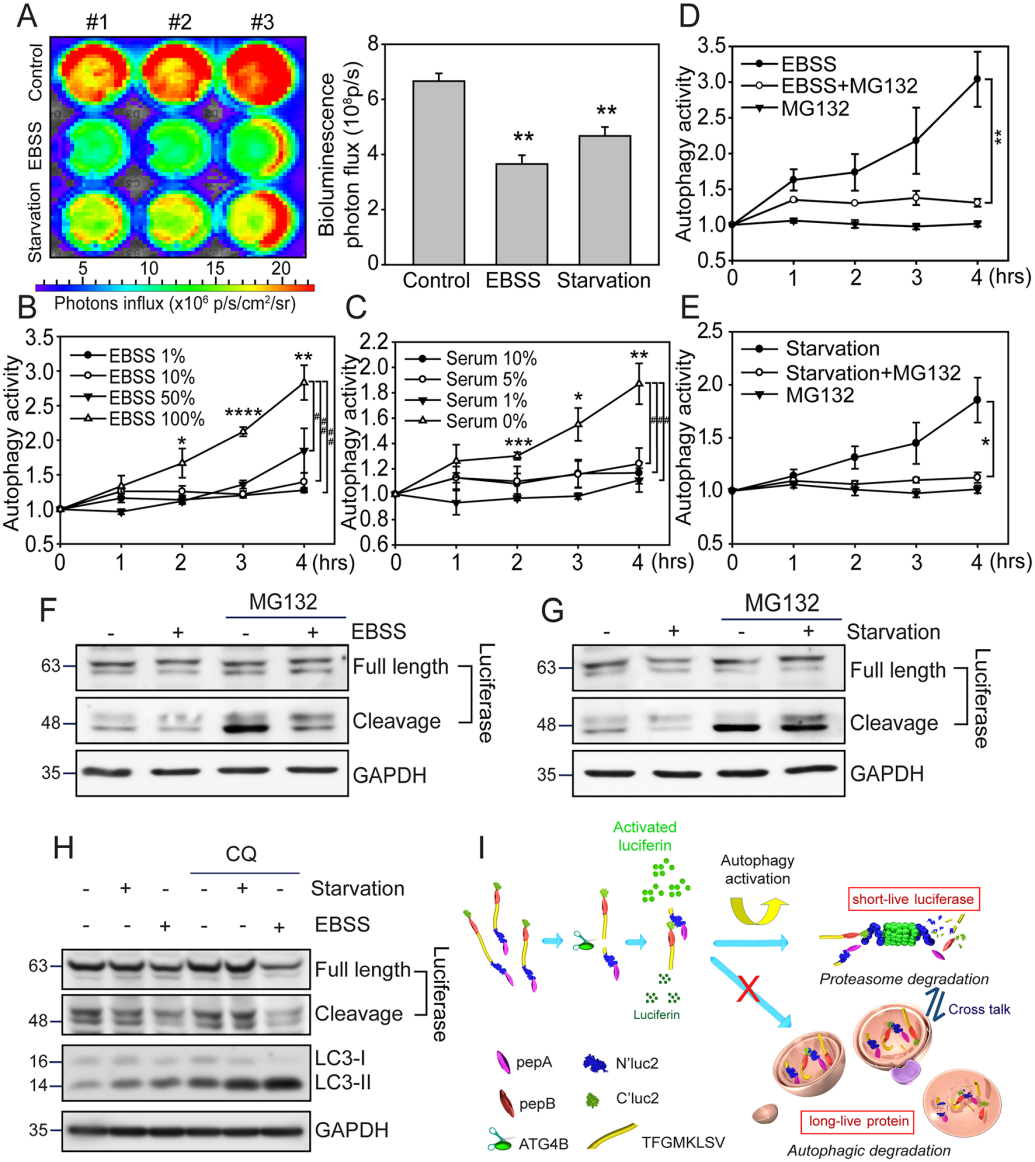
Enhanced autophagic flux and G-cleave LC3B biosensor luciferase activity in MDA-MB-231 cells through proteosome-related degradation. **A** IVIS images show the decline of bioluminescence in MDA-MB-231 cells expressed *pEGFP-LC3B*^*pepABLuc*^ with 4 h of EBSS and serum starvation treatments. Quantification of total photons flux was analyzed (n = 3 replicates; student-t test; **, *p* < 0.01; bars represent mean ± SE). MDA-MB-231 cells expressing *pEGFP-LC3B*^*pepABLuc*^ were exposed to varying concentrations of **B** EBSS and **C** serum starvation for 1 to 4 h, or pre-treatment with or without 10 μM MG132 followed by indicated incubation with **D** EBSS and **E** serum starvation. The luciferase degradation activity (convert to autophagy activity) was assessed as described in materials and methods (n = 3 replicates; student-t test; *, *p* < 0.05; **, *p* < 0.01; error bars represent mean ± SE). The lysates of 4 h **F** EBSS-treated and **G** serum starvation-treated were also collected and immunoblotted with anti-luciferase antibody. The full-length luciferase appeared at around 63 KDa, while the cleavage form was around 48 KDa on SDS-PAGE. **H** The level of cleaved luciferase and autophagy degradation was evaluated by immunoblotting in MDA-MB-231 cells expressing *pEGFP-LC3B*^*pepABLuc*^, treated with EBSS in the presence or absence of 30 μM CQ for 24 h. GAPDH was used as a loading control. **I** A schematic diagram of the G-cleave LC3B biosensor is presented. The fusion protein of pepA-N’luc2 and pepB-C’luc2 were linked with a 3X LC3B cleavage sequence (TFGMKLS). The bioluminescence expression of the cleavage form of luciferase mediated by ATG4B was detected upon the addition of luciferin. This bioluminescence is predominantly degraded in a proteosome-dependent manner during the activation of autophagy. The luciferase activity reflects the degradation ratio of luciferase upon complete autophagy activation

To investigate whether nutrient depletion efficiently activates the G-cleave LC3B biosensor, we employed a bioluminescence-based assay to monitor the luciferase degradation activity of the biosensor in MDA-MB-231 cells. We revealed that EBSS and serum starvation significantly decreased the bioluminescence activity in MDA-MB-231 cells (*p*≦0.01, Fig. 3A), which refers to the previous finding that autophagy elevated LC3B lipidation and cargo protein degradation. The maximum luciferase degradation activity of the G-cleave LC3B biosensor occurred four hours after EBSS or serum starvation, with no further increase after prolonged treatment (Supplementary Fig. 2). We further assessed the applicability of *pEGFP-LC3B*^*pepABLuc*^ biosensor in MDA-MB-453 and MDA-MB-468 TNBC cells, where EBSS and serum starvation also significantly decreased bioluminescence activity (*p*≦0.01, Supplementary Fig. 3A, B), with autophagy activity peaking at four hours post-treatment (*p*≦0.01, Supplementary Fig. 3C, D). Additionally, the G-cleave LC3B biosensor exhibited a titratable response in a dose-dependent manner with higher EBSS exposure (*p*≦0.01, Fig. 3B), demonstrating the biosensor's sensitivity to varying degrees of autophagy induction. In contrast, serum depletion resulted in a mild time-dependent increase in autophagy activity over 1–4 h (*p*≦0.01, Fig. 3C) we found that FBS concentrations between 1 and 5% did not produce significant changes in bioluminescence, suggesting that low FBS levels may sufficiently support cell survival and prevent autophagy activation. It is speculated that to surpass the threshold for autophagy induction, FBS concentrations should be reduced to less than 1% in cell culture conditions. Importantly, the biosensor showed no response in cells cultured with 10% serum within four hours, confirming that the observed signal loss was due to autophagy-specific cleavage rather than nonspecific degradation (Supplementary Fig. 4).

The proteolytic processing of LC3 by the ATG4 cysteine protease is essential for the initiation of autophagy membrane conjugation [36] and the ubiquitin-mediated autophagic degradation [37]. To determine whether the autophagy-triggered reduction in G-cleave LC3B bioluminescence in MDA-MB-231 cells requires a protease degradation process, we exposed cells to MG132, a potent proteasome inhibitor to examine the bioluminescence changes upon EBSS/serum starvation treatments. We found that the high autophagy activity of EBSS/serum starvation treatments was significantly reduced during MG132 exposure (*p*≦0.01 and *p*≦0.05), whereas MG132 treatment only maintained low autophagy activity (Fig. 3D, E). Since it is known that the UPS is the primary mechanism that degrades the short-lived protein [37], we proposed that luciferase, a cleaved form of LC3B^pepABLuc^, is degraded through a proteasome-associated pathway upon autophagy activation. Indeed, immunoblot results showed that both EBSS-(Fig. 3F) and serum starvation-(Fig. 3G) mediated luciferase expression can be enhanced (*p*≦0.05) in cells pre-treated with MG132. To investigate whether autophagy degradation responsible for the long-lived protein is involved in this luciferase degradation process, we treated cells with the autophagy lysosome-fusion inhibitor chloroquine (CQ) for 24 h. Unlike the effects of MG132, CQ did not attenuate the degradation of luciferase in response to EBSS or serum starvation (Fig. 3H). However, long-term luminescence degradation was partially reduced when CQ inhibited autophagy activity (*p*≦0.05, Supplementary Fig. 5A), indicating that the lysosome-mediated long-term degradation pathway is not the primary mechanism driving the G-cleave LC3B biosensor activity (Fig. 3I).

We further evaluated the biosensor response to non-canonical autophagy modulators, specifically Monensin (autophagy activator) and Bafilomycin A1 (autophagy inhibitor), which are involved in the CASM pathway [38, 39]. The G-cleave LC3B biosensor was found to be highly sensitive to canonical autophagy induction by EBSS (Fig. 3B and Supplementary Fig. 5A), but showed minimal response to non-canonical autophagy modulators, including Monensin (Supplementary Fig. 5B), CQ (Supplementary Fig. 5C) and Bafilomycin A1 (Supplementary Fig. 5D). Unlike nutrient depletion, which markedly increased luciferase degradation, non-canonical autophagy stimulation did not lead to a decrease in luciferase degradation, as confirmed by immunoblotting (Supplementary Fig. 6). The quantified immunoblot results indicated that the expression level of either full length or cleaved forms of luciferase modulated by Monensin were not significantly altered by bafliomycin A1 exposure (Supplementary Fig. 12).

Taken together, the above results provide evidence that autophagic stimuli such as EBSS and serum starvation trigger the rapid degradation of short-lived cleaved-luciferase and increase the autophagy activity of the G-cleave LC3B biosensor in MDA-MB-231 cells primarily via proteasome-related degradation mechanisms. The sensor's selectivity is driven by its responsiveness to short-lived, canonical autophagic degradation rather than long-lived non-canonical autophagy. This is evident as non-canonical autophagy stimuli do not enhance cleaved-luciferase degradation or LC3 lipidation, even in the presence of autophagy inhibitors (Fig. 3I & Supplementary Fig. 6).

### LC3B conjugating enzyme ATG4B is required for nutrient depletion-mediated activation of autophagy activity in G-cleave LC3B biosensors

**Fig. 4:**
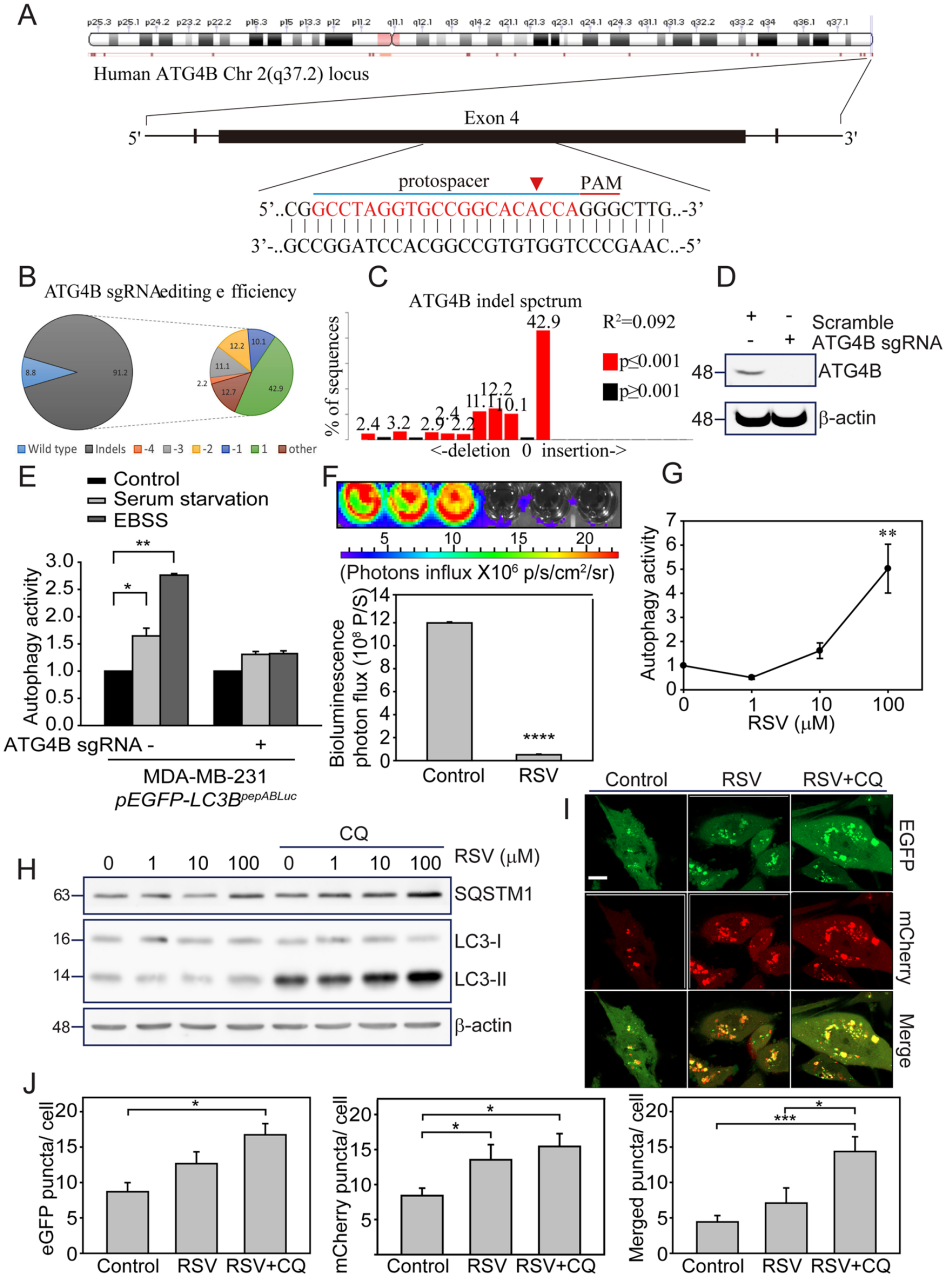
The specific autophagic roles of ATG4B and RSV sensitized the autophagy process of the G-cleave LC3B biosensor in MDA-MB-231. **A** The schematic of the CRISPR/Cas9 sgRNA targeting sequence located in the human ATG4B chromosome. TIDE analysis displayed the **B** gene editing efficiency and **C** gene indel spectrum of ATG4B DNA locus in MDA-MB-231 cells expressing *pEGFP-LC3B*^*pepABLuc*^. **D** Immunoblotting verified the depletion of ATG4B protein expression. **E** MDA-MB-231 cells expressing the *pEGFP-LC3B*^*pepABLuc*^ were transduced with lentivirus-mediated CRISPR/Cas9 targeting ATG4B or scrambled control (SC) and then treated with either EBSS or serum starvation for 4 h. The luciferase degradation activity (convert to autophagy activity) of the G-cleave LC3B biosensor was assessed. **F** MDA-MB-231 cells expressing *pEGFP-LC3B*^*pepABLuc*^ were treated with or without 100 μM RSV for 4 h. IVIS images were obtained, and quantification of total bioluminescence photon flux was analyzed. **G** MDA-MB-231 cells expressing *pEGFP-LC3B*^*pepABLuc*^ were treated with RSV at indicated concentrations for 4 h. Luciferase degradation activity (convert to autophagy activity) was determined as described in the materials and methods (n = 3 replicates; student-t test; *, *p* < 0.01; **, *p* < 0.01; error bars represent mean ± SE). **H** MDA-MB-231 cells were treated with 1 to 100 μM RSV for 24 h, with or without 30 μM CQ co-treatment. Cell lysates were collected, and the accumulation of LC3B-II and SQSTM1 was examined by immunoblotting. β-actin was used as a loading control. **I** MDA-MB-231 cells with *pmcherry-EGFP-LC3B* expression were treated with 25 μM RSV with or without 30 μM CQ for 24 h. Autophagic flux was determined by observing the formation of EGFP (green), mcherry (red), and merge (yellow/orange) puncta under the confocal microscopy. Scale bar: 10 μm. **J** Quantitate analysis of mCherry (red), EGFP (green), and merged (yellow/orange) puncta per cell are represented as means ± SEM in 20 to 30 cells in three independent experiments

The post-translational modification of LC3B is an essential step in autophagy, in which the C-terminus of the soluble LC3-I is cleaved by ATG4B, an autophagy-related cysteine protease, to expose the C-terminal glycine residue required for the formation of the membrane-bound LC3-II [40]. The G-cleave LC3B biosensor used in this study was designed to incorporate an autophagy cleavage sequence that contains the critical C-terminal glycine residue needed for LC3B-PE conjugation [29, 34]. To investigate the role of ATG4B in regulating the luciferase degradation (autophagy activity) of G-cleave LC3B biosensor, we used lentivirus-mediated CRISPR-Cas9 gene editing to establish ATG4B gene-edited MDA-MB-231 cells with *pEGFP-LC3B*^*pepABLuc*^ sensor (Fig. 4A). Through Tracking of Indels by DEcomposition (TIDE) analysis, we noticed that the average indels rate was 91.2% in ATG4B gene edited pool MDA-MB-231 cells with *pEGFP-LC3B*^*pepABLuc*^ sensor (Fig. 4B), whereas the most abundant indel was + 1 nucleotide insertion with 42.9% of all population (Fig. 4C). On the other hand, ATG4B protein expression was completely abolished in the ATG4B gene-edited pool MDA-MB-231 cells with *pEGFP-LC3B*^*pepABLuc*^ sensor, compared with SC parental cells (Fig. 4D). In addition, we found that the EBSS- and serum starvation- triggered autophagy activity of the G-cleave LC3B biosensor was significantly inhibited in ATG4B gene-edited cells (*p*≦0.01 and *p*≦0.05, Fig. 4E), indicating that the autophagic lipidation enzyme ATG4B is essential for initiating autophagy process and downstream proteasome-related degradation of G-cleave LC3B biosensor during autophagic stimulation.

### Screening of autophagy-modulating drugs by G-cleave LC3B biosensor in MDAMD231 breast cancer cells

To identify potent drugs that regulate autophagy in breast cancer, we performed a drug screening assay using the G-cleave LC3B biosensor, including clinical anti-breast cancer drugs, autophagy modulators, and flavonoids. The drug details used to validate the luciferase degradation activity (autophagy activity) of the biosensor in MDA-MB-231 cells are listed in Supplementary Table 2. We found that EBSS and serum starvation increased the autophagy activity of the biosensor by 2.82 ± 0.48 and 2.04 ± 0.19 folds, respectively (Supplementary Table 1). The autophagy-inducing peptide Tat-Beclin1 L11, which specifically activates autophagy via interaction with the autophagy suppressor GAPR-1/GLIPR2 [41], increased the autophagy activity by 3.89 folds (Supplementary Table 2). The autophagy inhibitors CQ and 3-MA did not alter the autophagy activity of the biosensor in MDA-MB-231 cells. Additionally, we found that Resveratrol (RSV), a natural anti-tumor phenol isolated from grapes, achieved the highest autophagy activity (4.31 folds) compared to other drugs as determined by the G-cleave LC3B biosensor in MDA-MB-231 cells. The IVIS assay also demonstrated significant biosensor degradation after RSV treatment (*p*≦0.0001, Fig. 4F).

Based on the drug pre-screening results (Supplementary Table 2), we found that RSV, a natural phenol known for its anti-tumoral properties [42], increased the autophagy activity of MDA-MD-231cells much more than other drugs. It was evident that RSV treatment at 10 to 100 µM increased autophagy activity dose-dependently (*p*≦0.01, Fig. 4G). Additionally, 24 h after RSV exposure, MDA-MB-231 maintained autophagy characteristics, such as LC3B lipidation and SQSTM1 degradation in cells with or without CQ (*p*≦0.05*,* Fig. 4H). RSV also promoted autophagy flux, as indicated by the formation of green (autophagosome) and red (autophagolysosome) puncta in *pmCherry-EGFP-LC3B*-transfected MDA-MB-231 cells (Fig. 4I). Additionally, CQ treatment led to enhanced accumulation of yellow/orange (autophagolysosome) puncta compared to RSV treatment alone (*p*≦0.05), as quantified from MDA-MB-231 cells (Fig. 4J). These findings highlight the effectiveness of the G-cleave LC3B biosensor in identifying promising autophagy-targeting drugs for potential TNBC treatments.

### RSV enhances the drug-sensitivity of DOX in MDA-MB-231 breast cancer cells

The anti-tumoral role of autophagy has also been reported in breast cancer [43]. Dysregulation of autophagy caused by the loss of the autophagy-related gene BECN1 facilitates tumor formation and progression in TNBC. Despite numerous studies aimed at verifying potential cancer therapeutic drug combinations by inhibiting or stimulating autophagy, no authorized pharmaceuticals are currently designed to manipulate autophagy for addressing TNBC. To explore whether selected autophagy agents synergize with clinical chemotherapy agents, such as doxorubicin (DOX) to improve the anti-cancer effect on TNBC, we combined RSV and DOX to treat MDA-MB-231 cells. The cytotoxicity determination showed that the IC50 concentration of RSV and DOX were 90 µM (Supplementary Fig. 7A) and 2.0 µM (Supplementary Fig. 7B), respectively. Since the development of drug resistance in malignant breast tumors is frequently observed, the combination of DOX with other anti-neoplastic agents is therefore required in clinical settings [44, 45]. Hence, we evaluated the anti-cancer potential of RSV in combination with DOX to suppress breast cancer cell survival and growth.

RSV is a natural compound that exhibited health benefits wildly, including its anti-cancer properties. The effective concentration of resveratrol for the anti-tumor study can vary depending on the specific type of cancer, the experimental conditions, and the study design. Typically, the effective anti-cancer concentration of RSV ranges from 50 to 100 µM, depending on the specific cancer. Research shows that long-term RSV treatment, or its combination with other chemotherapeutic agents, enhances therapeutic outcomes by promoting apoptosis and inhibiting cancer cell proliferation. This is particularly true for aggressive cancers like TNBC, where RSV has demonstrated efficacy in reducing tumor growth and increasing treatment sensitivity [46, 47]. RSV has been shown to inhibit the mechanistic target of rapamycin complex 1 (mTORC1), which facilitates early autophagosome formation by promoting the interaction between unc-51 like autophagy activating kinase 1 (ULK1) and the class III phosphatidylinositol 3-kinase (PtdIns3K) complex [48–50]. By inhibiting mTORC1, RSV activates autophagy and prevents the overactivation of the PI3K/Akt/mTOR pathway, a known contributor to DOX chemotherapy resistance. This dual action ultimately enhances apoptosis [48, 49]. Therefore, RSV may promote DOX-induced apoptosis through mTOR-dependent autophagy activation, offering potential therapeutic benefits in combating aggressive breast cancer progression.

**Fig. 5:**
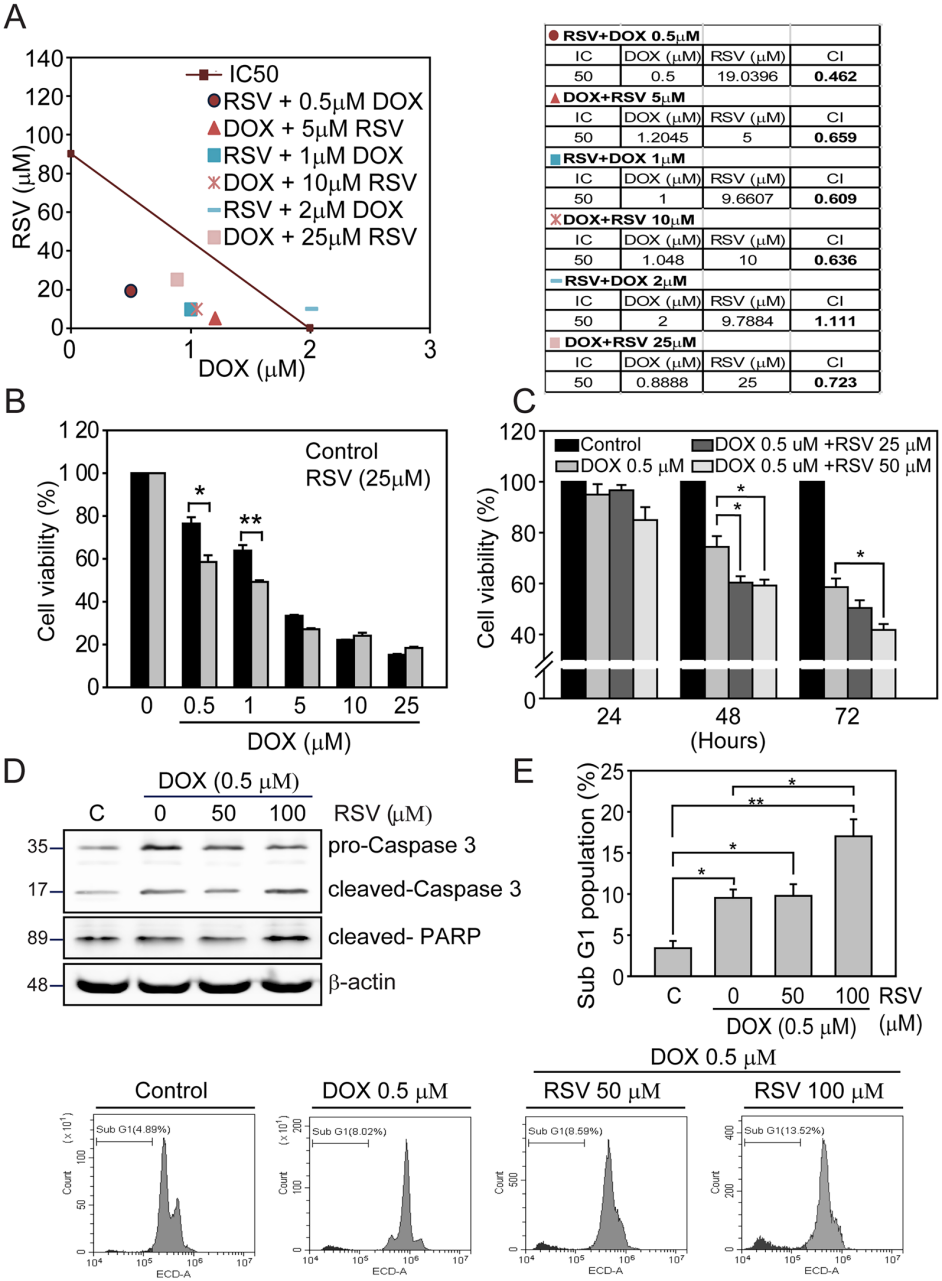
RSV potentiates DOX-induced cytotoxicity and apoptosis in MDA-MB-231 breast cancer cells. **A** The combination index (CI) of RSV and DOX was determined for 48 h on MDA-MB-231 cells by CCK-8 cell viability assay. An isobologram was generated to represent the synergistic/antagonistic effect of each combination. **B** MDA-MB-231 cells were treated with DOX (0.5 to 25 μM) with or without RSV (25 μM) for 48 h, and cell viability was measured by CCK-8 assay. **C** MDA-MB-231 cells were treated with a low concentration of DOX (0.5 μM) in combination with increasing concentrations of RSV (25 to 50 μM) for 24 to 72 h, and cell viability was measured by CCK-8 assay. (n = 3 replicates; student-t test; *, *p* < 0.05; **; error bars represent mean ± SE). MDA-MB-231 cells were treated with DOX (0.5 μM) with or without RSV (50 to 100 μM) for 48 h. **D** The activity of caspase 3 and PARP cleavage was examined by Immunoblot. β-actin was used as a loading control. **E** Flow cytometry determined the apoptosis cell population of the sub-G1 phase, and the percentage of sub-G1 cell populations are listed (n = 3 replicates; student-t test; *, *p* < 0.05; **; error bars represent mean ± SE)

As shown previously, the IC50 concentrations of RSV and DOX were determined to be 90 µM and 2.0 µM. To evaluate the potential synergistic cytotoxicity of RSV and DOX in MDA-MB-231 cells, we used the Combination Index (CI) algorithm to categorize drug combinations as additive (CI = 1), synergistic (CI < 1) or antagonistic (CI > 1), respectively (Fig. 5A). Among these combinations, co-treatment with 19.03 µM RSV and 0.5 µM DOX (red circle) achieved the greatest cytotoxic effect (CI = 0.462), indicating a strong synergy. Other combinations, such as reducing RSV or increasing DOX concentrations, did not result in better CI values. To confirm this finding, we tested fixed concentrations of RSV (25 µM) with varying concentrations of DOX (0.5–25 µM). The results demonstrated that the co-treatments of 25 µM RSV with 0.5 µM or 1 µM DOX showed the most significant cytotoxicity in MDA-MB-231 cells (*p*≦0.05 and *p*≦0.01, Fig. 5B). Additionally, in a time-dependent analysis, the combination of 25 µM or 50 µM RSV with 0.5 µM DOX led to a more pronounced reduction in cell viability compared to 0.5 µM DOX alone after 48 h of treatment (*p*≦0.05, Fig. 5C). Even after 72 h, the combination of 50 µM RSV with 0.5 µM DOX maintained a significant inhibitory effect on cell viability compared to DOX treatment alone (*p*≦0.05).

Lastly, we confirmed the above anti-cancer finding with immunoblotting. It was evident that the combined treatment with 100 µM of RSV and 0.5 µM DOX significantly induced caspase 3 and PARP cleavages on MDA-MB-231 cells (*p*≦0.05 and *p*≦0.001, Fig. 5D). In addition, we used flow cytometry to confirm the above synergic apoptosis activity of RSV and DOX exposures. After the combination drug treatments for 2 days, 100 µM of RSV and 0.5 µM DOX obtained the most effective apoptosis event (sub-G1 phase) than DOX monotherapy and control group (*p*≦0.01, Fig. 5E). These findings indicate that RSV, the anti-cancer compound identified from the G-cleave LC3B biosensor cells, enhances the cytotoxic and apoptotic effects during DOX exposure to MDA-MB-231, implying this potential anti-cancer drug combination may be used in clinical breast cancer therapy.

The toxicity of DOX is known to decrease in acidic conditions (pH 6.3) due to reduced cell membrane permeability [50]. Additionally, an acidic extracellular environment can hinder the efficacy of anti-cancer drugs by inhibiting autophagy [51]. However, autophagy can help cancer cells survive in acidic environments. All conditions were maintained at pH 7.3 to pH 7.6 to minimize pH interference in our experiments-the impact of acidic tumor microenvironments on drug efficacy identified by our biosensor warrants further investigation. Our findings suggest the potential use of biomaterials to mitigate acidic environmental effects, enhancing autophagy-based therapies in breast cancer.

### The advantage of G-cleave LC3B biosensor in monitoring of autophagy

The G-cleave LC3B biosensor offers several distinct advantages in the monitoring of autophagy. We summarized these advantages and compared them to traditional autophagy detection methods commonly employed in cancer research (Table 1). Firstly, conventional techniques such as immunoblotting and flow cytometry provide insights into the autophagy process by monitoring changes in specific autophagy-related proteins, allowing researchers to identify the stage of autophagy, whether it involves elongation, fusion, or protein degradation. However, these methods have significant drawbacks, including their time-consuming and expensive cost requirements, primarily due to the need for various antibodies. Secondly, imaging-based approaches, such as puncta formation and electron microscopy (EM), are frequently used to detect the autophagic flux and formation of the double-membrane autophagosome structure. While these techniques are more cost-effective than immunoblotting and flow cytometry, they demand a deep understanding of molecular cloning techniques, access to sophisticated fluorescence or electron microscopes, and, most importantly, the expertise of trained personnel to obtain reliable imaging results. Furthermore, the data obtained from these traditional methods, often image-based analysis, are either non-quantitative or semi-quantitative, limiting their utility for large-scale drug screening due to the substantial sample requirements. In contrast, our study introduces an innovative autophagy detection approach that is quantifiable through digital scale-based analysis, rapid and high-throughput. By culturing live cells that carry the G-cleave LC3B biosensor in multi-well plates, applying autophagy-inducing drugs less than 4 h, whereas researchers can obtain digitalized quantitative experimental data using a fluorescence/luminescence microplate reader within 30 min. The G-cleave LC3B biosensor minimizes sample requirement and provides high detection capacity in the multi-well plates, make it an efficient tool for autophagy drug screening. This method also holds great promise for advanced applications in precision medicine, enables the screening of autophagy/apoptosis-targeting drugs in clinical cancer patients, paving the way for more personalized and effective cancer treatments.

**Table 1:**
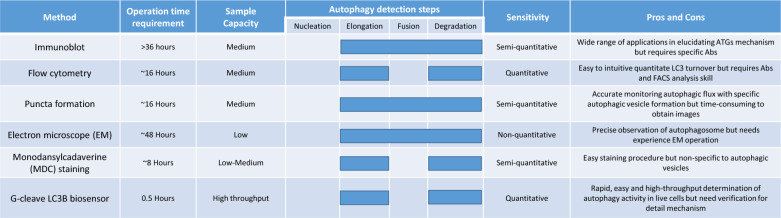
The comparison between G-cleave LC3 biosensor and other routine autophagy detection methods

## Conclusions

Our study highlights the significant potential of the G-cleave LC3B biosensor, a groundbreaking biotechnological innovation, in screening candidate drugs that target autophagy. This technology focuses on the autophagic process, particularly the elongation and degradation of the LC3B Glycine cleavage. What sets this biosensor apart is its ability to swiftly and quantitatively monitor autophagy activation, mainly by measuring bioluminescence quenching value with mathematic calculation. This rapid assessment eliminates the need for time-consuming experimental procedures and intricate techniques, making it an ideal tool for expeditious screening of potential autophagy-related anticancer drugs, ultimately improving the effectiveness for developing cancer therapeutic strategy. Additionally, our comprehensive screening efforts have unveiled resveratrol's capacity to enhance and synergistically induce apoptotic effects when combined with DOX, a highly effective chemotherapeutic regimen for treating triple-negative breast cancer, proposing RSV as a potential adjunct therapy for breast cancer treatment, underlining the critical role of autophagy modulation in enhancing the overall efficacy of cancer treatments.

## Supplementary Information

Supplementary material 1

Supplementary material 2

Supplementary material 3

## Data Availability

No datasets were generated or analysed during the current study.

## Abbreviations

ATG: Autophagy-related, BECN1, NIADS-autophagy, non-invasive autophagy detection system
EBSS: Earle's balanced salt solution
G-cleave LC3B: LC3B cleaving peptide
LC3B: Microtubule-associated protein 1 light chain 3 beta
PE: Phosphatidylethanolamine
IVIS: In vivo imaging system
CQ: Chloroquine
3-MA: 3-Methyladenine
SQSTM1/p62: Sequestosome-1/ubiquitin binding protein p62
RSV: Resveratrol
DOX: Doxorubicin
TNBC: Triple-negative breast cancer
PARP: Poly(ADP-ribose) polymerase

## References

[1] Li X, He S, Ma B. Autophagy and autophagy-related proteins in cancer. Mol Cancer. 2020;19(1):12. 31969156 10.1186/s12943-020-1138-4 PMC6975070

[2] Mizushima N. The ATG conjugation systems in autophagy. Curr Opin Cell Biol. 2020;63:1–10. 31901645 10.1016/j.ceb.2019.12.001

[3] Parzych KR, Klionsky DJ. An overview of autophagy: morphology, mechanism, and regulation. Antioxid Redox Signal. 2014;20(3):460–73. 23725295 10.1089/ars.2013.5371 PMC3894687

[4] Nedelsky NB, Todd PK, Taylor JP. Autophagy and the ubiquitin-proteasome system: collaborators in neuroprotection. Biochim Biophys Acta. 2008;1782(12):691–9. 18930136 10.1016/j.bbadis.2008.10.002 PMC2621359

[5] Li Y, Li S, Wu H. Ubiquitination-proteasome system (UPS) and autophagy two main protein degradation machineries in response to cell stress. Cells. 2022;11(5):851. 35269473 10.3390/cells11050851 PMC8909305

[6] Lamark T, Svenning S, Johansen T. Regulation of selective autophagy: the p62/SQSTM1 paradigm. Essays Biochem. 2017;61(6):609–24. 29233872 10.1042/EBC20170035

[7] Ferrari R, Kia DA, Tomkins JE, Hardy J, Wood NW, Lovering RC, Lewis PA, Manzoni C. Stratification of candidate genes for Parkinson’s disease using weighted protein-protein interaction network analysis. BMC Genom. 2018;19(1):452. 10.1186/s12864-018-4804-9 PMC6000968 29898659

[8] Vazquez-Martin A, Oliveras-Ferraros C, Menendez JA. Autophagy facilitates the development of breast cancer resistance to the anti-HER2 monoclonal antibody trastuzumab. PLoS ONE. 2009;4(7):e6251. 19606230 10.1371/journal.pone.0006251 PMC2708925

[9] Wang M, Liu M, Yang C, Hu Y, Liao X, Liu Q. Autophagy modulation in therapeutic strategy of breast cancer drug resistance. J Cancer. 2024;15(16):5462–76. 39247603 10.7150/jca.97775 PMC11375553

[10] Maycotte P, Thorburn A. Targeting autophagy in breast cancer. World J Clin Oncol. 2014;5(3):224–40. 25114840 10.5306/wjco.v5.i3.224 PMC4127596

[11] Vera-Ramirez L, Vodnala SK, Nini R, Hunter KW, Green JE. Autophagy promotes the survival of dormant breast cancer cells and metastatic tumour recurrence. Nat Commun. 2018;9(1):1944. 29789598 10.1038/s41467-018-04070-6 PMC5964069

[12] Babavalian A, Tekie FSM, Ayazi H, Ranjbar S, Varshochian R, Rad-Malelkshahi M, Akhavan O, Dinarvand R. Reduced polydopamine coated graphene for delivery of Hset1 antisense as A photothermal and gene therapy of breast cancer. J Drug Deliv Sci Technol. 2022;73:103462.

[13] Hatamie S, Akhavan O, Sadrnezhaad SK, Ahadian MM, Shirolkar MM, Wang HQ. Curcumin-reduced graphene oxide sheets and their effects on human breast cancer cells. Mater Sci Eng C Mater Biol Appl. 2015;55:482–9. 26117780 10.1016/j.msec.2015.05.077

[14] Mintz RL, Gao MA, Lo K, Lao YH, Li M, Leong KW. CRISPR technology for breast cancer: diagnostics, modeling, and therapy. Adv Biosyst. 2018;2(11):1800132. 32832592 10.1002/adbi.201800132 PMC7437870

[15] Chiu CF, Chin HK, Huang WJ, Bai LY, Huang HY, Weng JR. Induction of apoptosis and autophagy in breast cancer cells by a novel HDAC8 inhibitor. Biomolecules. 2019;9(12):824. 31817161 10.3390/biom9120824 PMC6995545

[16] Alhoshani A, Alatawi FO, Al-Anazi FE, Attafi IM, Zeidan A, Agouni A, El Gamal HM, Shamoon LS, Khalaf S, Korashy HM. BCL-2 inhibitor venetoclax induces autophagy-associated cell death, cell cycle arrest, and apoptosis in human breast cancer cells. Onco Targets Ther. 2020;13:13357–70. 33414642 10.2147/OTT.S281519 PMC7783200

[17] Kaizuka T, Morishita H, Hama Y, Tsukamoto S, Matsui T, Toyota Y, Kodama A, Ishihara T, Mizushima T, Mizushima N. An autophagic flux probe that releases an internal control. Mol Cell. 2016;64(4):835–49. 27818143 10.1016/j.molcel.2016.09.037

[18] Beesabathuni NS, Park S, Shah PS. Quantitative and temporal measurement of dynamic autophagy rates. Autophagy. 2023;19(4):1164–83. 36026492 10.1080/15548627.2022.2117515 PMC10012960

[19] Mizushima N, Yoshimori T, Levine B. Methods in mammalian autophagy research. Cell. 2010;140(3):313–26. 20144757 10.1016/j.cell.2010.01.028 PMC2852113

[20] Ming H, Li B, Tian H, Zhou L, Jiang J, Zhang T, Qiao L, Wu P, Nice EC, Zhang W, et al. A minimalist and robust chemo-photothermal nanoplatform capable of augmenting autophagy-modulated immune response against breast cancer. Mater Today Bio. 2022;15:100289. 35634171 10.1016/j.mtbio.2022.100289 PMC9130112

[21] Assali A, Akhavan O, Adeli M, Razzazan S, Dinarvand R, Zanganeh S, Soleimani M, Dinarvand M, Atyabi F. Multifunctional core-shell nanoplatforms (gold@graphene oxide) with mediated NIR thermal therapy to promote miRNA delivery. Nanomedicine. 2018;14(6):1891–903. 29885900 10.1016/j.nano.2018.05.016

[22] Alkaraki A, Alshaer W, Wehaibi S, Gharaibeh L, Abuarqoub D, Alqudah DA, Al-Azzawi H, Zureigat H, Souleiman M, Awidi A. Enhancing chemosensitivity of wild-type and drug-resistant MDA-MB-231 triple-negative breast cancer cell line to doxorubicin by silencing of STAT 3, Notch-1, and beta-catenin genes. Breast Cancer. 2020;27(5):989–98. 32328816 10.1007/s12282-020-01098-9

[23] Assali A, Akhavan O, Mottaghitalab F, Adeli M, Dinarvand R, Razzazan S, Arefian E, Soleimani M, Atyabi F. Cationic graphene oxide nanoplatform mediates miR-101 delivery to promote apoptosis by regulating autophagy and stress. Int J Nanomed. 2018;13:5865–86. 10.2147/IJN.S162647 PMC6171513 30319254

[24] Kauffman KJ, Yu S, Jin J, Mugo B, Nguyen N, O’Brien A, Nag S, Lystad AH, Melia TJ. Delipidation of mammalian Atg8-family proteins by each of the four ATG4 proteases. Autophagy. 2018;14(6):992–1010. 29458288 10.1080/15548627.2018.1437341 PMC6103404

[25] Chen SH, Chow JM, Hsieh YY, Lin CY, Hsu KW, Hsieh WS, Chi WM, Shabangu BM, Lee CH. HDAC1,2 knock-out and HDACi induced cell apoptosis in imatinib-resistant K562 cells. Int J Mol Sci. 2019;20(9):2271. 31071955 10.3390/ijms20092271 PMC6539538

[26] Hsu KW, Huang CY, Tam KW, Lin CY, Huang LC, Lin CL, Hsieh WS, Chi WM, Chang YJ, Wei PL, et al. The application of non-invasive apoptosis detection sensor (NIADS) on histone deacetylation inhibitor (HDACi)-induced breast cancer cell death. Int J Mol Sci. 2018;19(2):452. 29393914 10.3390/ijms19020452 PMC5855674

[27] Lin CL, Tsai ML, Chen YH, Liu WN, Lin CY, Hsu KW, Huang CY, Chang YJ, Wei PL, Chen SH, et al. Platelet-derived growth factor receptor-alpha subunit targeting suppresses metastasis in advanced thyroid cancer in vitro and in vivo. Biomol Ther (Seoul). 2021;29(5):551–61. 34031270 10.4062/biomolther.2020.205 PMC8411021

[28] Lin CL, Tsai ML, Lin CY, Hsu KW, Hsieh WS, Chi WM, Huang LC, Lee CH. HDAC1 and HDAC2 double knockout triggers cell apoptosis in advanced thyroid cancer. Int J Mol Sci. 2019;20(2):454. 30669676 10.3390/ijms20020454 PMC6359659

[29] Tanida I, Ueno T, Kominami E. Human light chain 3/MAP1LC3B is cleaved at its carboxyl-terminal Met121 to expose Gly120 for lipidation and targeting to autophagosomal membranes. J Biol Chem. 2004;279(46):47704–10. 15355958 10.1074/jbc.M407016200

[30] Chen SH, Hsieh YY, Tzeng HE, Lin CY, Hsu KW, Chiang YS, Lin SM, Su MJ, Hsieh WS, Lee CH. ABL genomic editing sufficiently abolishes oncogenesis of human chronic myeloid leukemia cells in vitro and in vivo. Cancers (Basel). 2020;12(6):1399. 32485885 10.3390/cancers12061399 PMC7352505

[31] Lin CY, Lee CH, Chuang YH, Lee JY, Chiu YY, Wu Lee YH, Jong YJ, Hwang JK, Huang SH, Chen LC, et al. Membrane protein-regulated networks across human cancers. Nat Commun. 2019;10(1):3131. 31311925 10.1038/s41467-019-10920-8 PMC6635409

[32] Galluzzi L, Pietrocola F, Bravo-San Pedro JM, Amaravadi RK, Baehrecke EH, Cecconi F, Codogno P, Debnath J, Gewirtz DA, Karantza V, et al. Autophagy in malignant transformation and cancer progression. EMBO J. 2015;34(7):856–80. 25712477 10.15252/embj.201490784 PMC4388596

[33] Mulcahy Levy JM, Thorburn A. Autophagy in cancer: moving from understanding mechanism to improving therapy responses in patients. Cell Death Differ. 2020;27(3):843–57. 31836831 10.1038/s41418-019-0474-7 PMC7206017

[34] Kabeya Y, Mizushima N, Ueno T, Yamamoto A, Kirisako T, Noda T, Kominami E, Ohsumi Y, Yoshimori T. LC3, a mammalian homologue of yeast Apg8p, is localized in autophagosome membranes after processing. EMBO J. 2000;19(21):5720–8. 11060023 10.1093/emboj/19.21.5720 PMC305793

[35] Orhon I, Reggiori F. Assays to monitor autophagy progression in cell cultures. Cells. 2017;6(3):20. 28686195 10.3390/cells6030020 PMC5617966

[36] Kaminskyy V, Zhivotovsky B. Proteases in autophagy. Biochim Biophys Acta. 2012;1824(1):44–50. 21640203 10.1016/j.bbapap.2011.05.013

[37] Kocaturk NM, Gozuacik D. Crosstalk between mammalian autophagy and the ubiquitin-proteasome system. Front Cell Dev Biol. 2018;6:128. 30333975 10.3389/fcell.2018.00128 PMC6175981

[38] Durgan J, Lystad AH, Sloan K, Carlsson SR, Wilson MI, Marcassa E, Ulferts R, Webster J, Lopez-Clavijo AF, Wakelam MJ, et al. Non-canonical autophagy drives alternative ATG8 conjugation to phosphatidylserine. Mol Cell. 2021;81(9):2031. 33909989 10.1016/j.molcel.2021.03.020 PMC8122138

[39] Hooper KM, Jacquin E, Li T, Goodwin JM, Brumell JH, Durgan J, Florey O. V-ATPase is a universal regulator of LC3-associated phagocytosis and non-canonical autophagy. J Cell Biol. 2022;221(6):e202105112. 35511089 10.1083/jcb.202105112 PMC9082624

[40] Tanida I, Ueno T, Kominami E. LC3 conjugation system in mammalian autophagy. Int J Biochem Cell Biol. 2004;36(12):2503–18. 15325588 10.1016/j.biocel.2004.05.009 PMC7129593

[41] Shoji-Kawata S, Sumpter R, Leveno M, Campbell GR, Zou Z, Kinch L, Wilkins AD, Sun Q, Pallauf K, MacDuff D, et al. Identification of a candidate therapeutic autophagy-inducing peptide. Nature. 2013;494(7436):201–6. 23364696 10.1038/nature11866 PMC3788641

[42] Han G, Xia J, Gao J, Inagaki Y, Tang W, Kokudo N. Anti-tumor effects and cellular mechanisms of resveratrol. Drug Discov Ther. 2015;9(1):1–12. 25788047 10.5582/ddt.2015.01007

[43] Ahn JS, Ann EJ, Kim MY, Yoon JH, Lee HJ, Jo EH, Lee K, Lee JS, Park HS. Autophagy negatively regulates tumor cell proliferation through phosphorylation dependent degradation of the Notch1 intracellular domain. Oncotarget. 2016;7(48):79047–63. 27806347 10.18632/oncotarget.12986 PMC5346697

[44] Liu S, Li R, Qian J, Sun J, Li G, Shen J, Xie Y. Combination therapy of doxorubicin and quercetin on multidrug-resistant breast cancer and their sequential delivery by reduction-sensitive hyaluronic acid-based conjugate/d-alpha-tocopheryl poly(ethylene glycol) 1000 succinate mixed micelles. Mol Pharm. 2020;17(4):1415–27. 32159961 10.1021/acs.molpharmaceut.0c00138

[45] Tang Y, Soroush F, Tong Z, Kiani MF, Wang B. Targeted multidrug delivery system to overcome chemoresistance in breast cancer. Int J Nanomed. 2017;12:671–81. 10.2147/IJN.S124770 PMC5268372 28176940

[46] Liang ZJ, Wan Y, Zhu DD, Wang MX, Jiang HM, Huang DL, Luo LF, Chen MJ, Yang WP, Li HM, et al. Resveratrol mediates the apoptosis of triple negative breast cancer cells by reducing POLD1 expression. Front Oncol. 2021;11:569295. 33747905 10.3389/fonc.2021.569295 PMC7970754

[47] Rauf A, Imran M, Butt MS, Nadeem M, Peters DG, Mubarak MS. Resveratrol as an anti-cancer agent: a review. Crit Rev Food Sci Nutr. 2018;58(9):1428–47. 28001084 10.1080/10408398.2016.1263597

[48] Alayev A, Berger SM, Kramer MY, Schwartz NS, Holz MK. The combination of rapamycin and resveratrol blocks autophagy and induces apoptosis in breast cancer cells. J Cell Biochem. 2015;116(3):450–7. 25336146 10.1002/jcb.24997 PMC4491987

[49] Basho RK, Gilcrease M, Murthy RK, Helgason T, Karp DD, Meric-Bernstam F, Hess KR, Herbrich SM, Valero V, Albarracin C, et al. Targeting the PI3K/AKT/mTOR pathway for the treatment of mesenchymal triple-negative breast cancer: evidence from a phase 1 trial of mTOR inhibition in combination with liposomal doxorubicin and bevacizumab. JAMA Oncol. 2017;3(4):509–15. 27893038 10.1001/jamaoncol.2016.5281

[50] Trebinska-Stryjewska A, Swiech O, Opuchlik LJ, Grzybowska EA, Bilewicz R. Impact of medium pH on DOX toxicity toward HeLa and A498 cell lines. ACS Omega. 2020;5(14):7979–86. 32309708 10.1021/acsomega.9b04479 PMC7161040

[51] Pellegrini P, Strambi A, Zipoli C, Hagg-Olofsson M, Buoncervello M, Linder S, De Milito A. Acidic extracellular pH neutralizes the autophagy-inhibiting activity of chloroquine: implications for cancer therapies. Autophagy. 2014;10(4):562–71. 24492472 10.4161/auto.27901 PMC3984580

