# Supplementary material for: G-cleave LC3B biosensor: monitoring autophagy and assessing resveratrol's synergistic impact on doxorubicin-induced apoptosis in breast cancer cells
Source: Breast Cancer Res. 2024 Dec 30;26:190. doi: 10.1186/s13058-024-01951-1 (PMC11687128; doi:10.1186/s13058-024-01951-1)
Supplement: Supplementary file 1 — Supplementary material 1 [file 13058_2024_1951_MOESM1_ESM.docx]

**Supplementary tables and figures for:**

**G-cleave LC3B Biosensor: Monitoring Autophagy and Enhancing the Synergistic Effects of Resveratrol and Doxorubicin-Induced Apoptosis in Breast Cancer Cells**

Chiao-Chun Liao^1,2,3,4^, Yuqing Long^5,6^, Ming-Lin Tsai^7^, Chun-Yu Lin^8,9^, Kai-Wen Hsu^10,11^ and Chia-Hwa Lee^4,9,12,13^*

^1^ Department of Tropical Medicine, National Yang Ming Chiao Tung University, Taipei, Taiwan

^2^ School of Medicine, College of Medicine, National Yang Ming Chiao Tung University, Taipei, Taiwan

^3^ Institute of Public Health and Department of Social Medicine, National Yang Ming Chiao Tung University, Taipei, Taiwan

^4^ School of Medical Laboratory Science and Biotechnology, College of Medical Science and Technology, Taipei Medical University, New Taipei City, Taiwan

^5^ Nuffield Department of Medicine, University of Oxford, Oxford, UK

^6^ Chinese Academy of Medical Science Oxford Institute, University of Oxford, Oxford, UK

^7^ Department of General Surgery, Cathay General Hospital, Taipei, Taiwan

^8^ Institute of Bioinformatics and Systems Biology, National Chiao Tung University, Hsinchu, Taiwan

^9^ Center for Intelligent Drug Systems and Smart Bio-devices (IDS2B), National Chiao Tung University, Hsinchu, Taiwan

^10^ Institute of New Drug Development, China Medical University, Taichung City, Taiwan

^11^ Research Center for Cancer Biology, China Medical University, Taichung City, Taiwan

^12^ TMU Research Center of Cancer Translational Medicine, Taipei Medical University, Taipei, Taiwan

^13^ Ph.D. Program in Medicine Biotechnology, College of Medicine, Taipei Medical University, New Taipei City, Taiwan

***Correspondence to:** Chia-Hwa Lee; Email: @tmu.edu.tw

**Contents**

**Supplementary Table 1………………………………………………………………………………S3**

**Supplementary Table 2………………………………………………………………………………S4**

**Supplementary Figure 1……………………………………………………………………………S5**

**Supplementary Figure 2……………………………………………………………………………S6**

**Supplementary Figure 3……………………………………………………………………………S7**

**Supplementary Figure 4……………………………………………………………………………S8**

**Supplementary Figure 5……………………………………………………………………………S9**

**Supplementary Figure 6……………………………………………………………………………S10**

**Supplementary Figure 7……………………………………………………………………………S11**

**Supplementary Figure 8……………………………………………………………………………S12**

**Supplementary Figure 9……………………………………………………………………………S13**

**Supplementary Figure 10…………………………………………………………………………S14**

**Supplementary Figure 11…………………………………………………………………………S15**

**Supplementary Figure 12…………………………………………………………………………S16**

**Supplementary Table 1**

| **Canonical autophagy activator** | **Degradation Fold** | **Activity** |
| --- | --- | --- |
| Earle's Balanced Salt Solution (EBSS) | 0.3764 ± 0.064 | 2.82 ± 0.4755 |
| Serum starvation | 0.4986 ± 0.0487 | 2.04 ± 0.19 |

**Supplementary Table 1. The gold-standard autophagy induction methods.**

We employed EBSS and starvation medium to induce autophagy on MDA-MB-231 cells. The table reveals the bioluminescent degradation in turns of autophagy activity.

**Supplementary Table 2**

| **Category** | **Mechanism** | **Name** | **Dose** | **Degradation Fold** | **Autophagy activity** |
| --- | --- | --- | --- | --- | --- |
| Anti-breast cancer drugs | HDAC inhibitor | Panobinostat | 1 μM | 1.22 ± 0.06 | 0.83 ± 0.04 |
|  |  | Belinostat | 1 μM | 1.37 ± 0.27 | 0.81 ± 0.14 |
|  |  | Vorinostat | 1 μM | 1.66 ± 0.22 | 0.64 ± 0.09 |
|  | PARP inhibitor | Olaparib | 1 μM | 1.09 ± 0.19 | 1.02 ± 0.20 |
|  | Topoisomerase II inhibitor | Doxorubicin | 1 μM | 1.38 ± 0.20 | 0.78 ± 0.12 |
|  | Mitotic arrest | Paclitaxel | 1 μM | 0.64 ± 0.07 | 1.64+ ± 0.23 |
|  | ErbB family inhibitor | Afatinib | 1 μM | 0.57 ± 0.06 | 1.83 ± 0.19 |
|  | EGFR/Tyrosin Kinase inhibitor | Lapatinib | 1 μM | 0.62 ± 0.05 | 1.63 ± 0.12 |
| Autophagy modulator | BECN1-activating peptide | Tat-Beclin1 L11 | 20 μM | 0.26 ± 0.02 | 3.89 ± 0.36 |
|  | mTOR inhibitor | Rapamycin | 100 μM | 1.35 ± 0.23 | 0.82 ± 0.15 |
|  | Caloric restriction mimetic | Resveratrol (Flavonoid) | 100 μM | 0.24 ± 0.02 | 4.31 ± 0.33 |
|  | Blockage of autophagosome-lysosome fusion | CQ | 30 mM | 1.13 ± 0.17 | 0.96 ± 0.15 |
|  | Type III Phosphatidylinositol 3-kinases inhibitor | 3-MA | 5 mM | 0.98 ± 0.16 | 1.13 ± 0.22 |
|  | Non-canonical autophagy activator | Monensin | 100 μM | 0.99 ± 0.02 | 1.01 ± 0.02 |
|  | Vacuolar H^+^-ATPase Inhibitor | Bafilomycin A1 | 100 nM | 0.98 ± 0.02 | 1.02 ± 0.02 |
| Flavonoid | Antioxidant and anti-inflammatory agent | Quercetin | 100 μM | 2.05 ± 0.08 | 0.49 ± 0.02 |
|  | anti-neoplastic agent | Curcumin | 100 μM | 1.40 ± 0.13 | 0.73 ± 0.07 |

**Supplementary Table 2. The potential autophagy induction agents.**

In order to discover potential autophagy induction agents, we screened various anti-breast cancer drugs, autophagy modulators and flavonoids on G-cleave LC3B biosensor activity of MDA-MB-231 cells for 4 hours. By determining bioluminescence degradation, we calculated the autophagy activity of each agent.

**Supplementary Figure 1**

**Supplementary Figure 1. Establishment of G-cleave LC3B biosensor on MDA-MB-453 and MDA-MB-468 breast cancer cells.**

Lentivirus-mediated *pEGFP-LC3B^pepABLuc^* expressions on (A) MDA-MB-453 and (B) MDA-MB-468 cells were visualized by fluorescence microscope. Scale bar: 50 μm. The breast cancer cells carried *pEGFP-LC3B^pepABLuc^* expression were determined with EGFP and luciferase protein expressions.

**Supplementary Figure 2**

**Supplementary Figure 2. The luciferase activity of G-cleave LC3B biosensor in response to long-term autophagic stimulation.**

MDA-MB-231 cells expressing *pEGFP-LC3B^pepABLuc^* (G-cleave LC3B biosensor) were subjected to (A) EBSS treatment or (B) serum starvation for 4 to 24 hours, and the luciferase degradation activity (autophagy activity) was measured as described in materials and methods (n = 3 replicates).

**Supplementary Figure 3**

**Supplementary Figure 3. Enhanced autophagic flux and G-cleave LC3B biosensor luciferase activity in MDA-MB-453 and MDA-MB-468 cells.**

IVIS images show the decline of bioluminescence in (A) MDA-MB-453 and (B) MDA-MB-468 cells expressed *pEGFP-LC3B^pepABLuc^* with 4 hours of EBSS and serum starvation treatments. Quantification of total photons flux was analyzed (n = 3 replicates; student-t test; **, *p*≦0.01; bars represent mean ± SE). Bioluminescent from both (C) MDA-MB-453 and (D) MDA-MB-468 cells expressing *pEGFP-LC3B^pepABLuc^* were determined with EBSS and serum starvation for 1 to 4 hours treatments. The luciferase degradation activity (convert to autophagy activity) was assessed as described in materials and methods (n = 3 replicates; student-t test; *, *p*≦0.05; **, *p*≦0.01; error bars represent mean ± SE).

**Supplementary Figure 4**


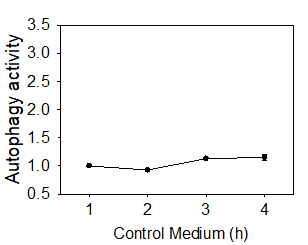


**Supplementary Figure 4. The autophagy activity of G-cleave LC3B biosensor in response to the culture medium.**

MDA-MB-231 cells expressing *pEGFP-LC3B^pepABLuc^* (G-cleave LC3B biosensor) were cultured in DMEM/F12 medium supplied with 10% serum for 1 to 4 hours, and the luciferase degradation activity (autophagy activity) was measured as described in materials and methods (n = 3 replicates; error bars represent mean ± SE).

**Supplementary Figure 5**


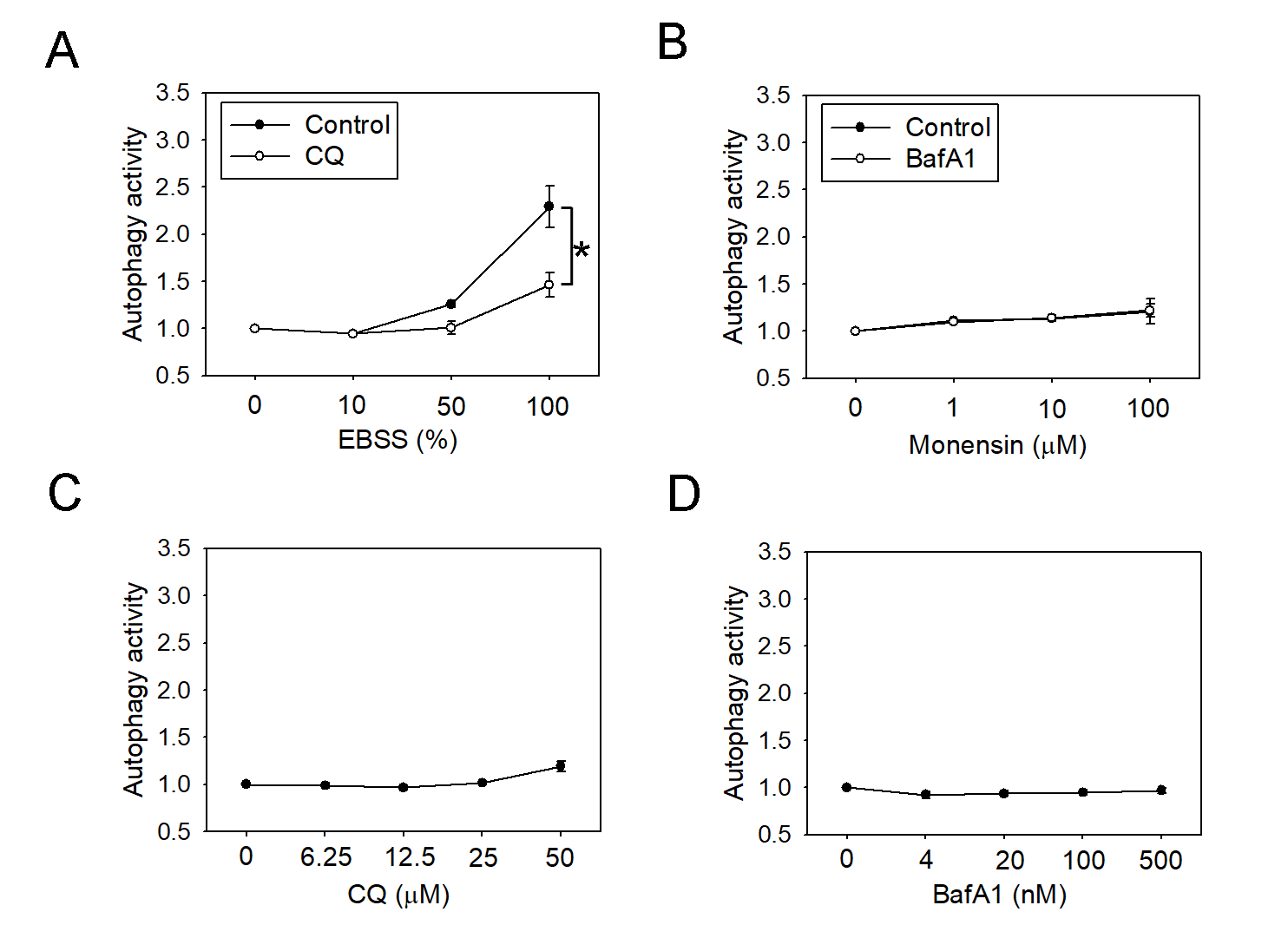


**Supplementary Figure 5. The autophagy activity of G-cleave LC3B biosensor in response to the canonical and non-canonical autophagy activator and inhibitor.**

MDA-MB-231 cells expressing *pEGFP-LC3B^pepABLuc^* (G-cleave LC3B biosensor) were subjected to (A) 0% to 100% EBSS with or without 16-hour pretreatment of 30 μM CQ, (B) 0 to 100 μM Monensin with or without 30 min pretreatment of 100 nM bafilomycin A1, (C) 0 to 50 μM CQ or (D) 0 to 500 nM bafilomycin A1 treatments for 4 hours, the luciferase degradation activity (autophagy activity) was measured as described in materials and methods (n = 3 replicates; student-t test; *, *p*≦0.05; error bars represent mean ± SE).

**Supplementary Figure 6.**


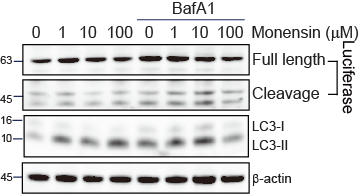


**Supplementary Figure 6. The autophagic effects of Monensin and BafA1 on the G-cleave LC3B biosensor in MDA-MB-231.**

MDA-MB-231 cells expressing *pEGFP-LC3B^pepABLuc^* were subjected to pre-treatment with or without 100 nM BafA1 autophagy inhibitor, followed by 4 hours with 1 to 100 μM Monensin treatments. The lysates were collected and immunoblotted with anti-luciferase and LC3B antibodies. β-actin was used as a loading control. The protein expression density of blots was quantitated in Supplementary Figure 12 by at least three replicates.

**Supplementary Figure 7**

**

**

**Supplementary Figure 7. Cell viability assessment of RSV and DOX in MDA-MB-231 breast cancer cells.**

The cell viability assay was conducted to determine the half-maximal inhibitory concentration (IC50) of (A) RSV and (B) DOX treatments on MDA-MB-231 breast cancer cells. The experiments were carried out over a 48-hour duration using the CCK-8 cell viability assay.

**Supplementary Figure 8**

**Supplementary Figure 8. Statistical protein expression analysis of Figure 1F.**

Cell lysates from *pEGFP-LC3B^pepABLuc^* expression cells were collected with or without 30 μM CQ. The expressions of EGFP, full-length luciferase, autophagic lipidation (LC3B-II) and degradation (SQSTM1) were determined by immunoblot. β-actin was used as a loading control. The protein expression density of blots was quantitated by at least three replicates (error bars represent mean ± SE; *, *p*≦0.05; **, *p*≦0.01; ***, *p*≦0.001)

**Supplementary Figure 9**

**Supplementary Figure 9. Statistical protein expression analysis of Figure 2A.**

MDA-MB-231 cells expressing *pEGFP-LC3B^pepABLuc^* were subjected to either EBSS or serum starvation for varying durations as indicated. (Figure 2B) MDA-MB-231 cells expressing *pEGFP-LC3B^pepABLuc^* were subjected to EBSS or serum starvation in the presence or absence of 30 μM CQ for 24 hours. Cell lysates were collected and subjected to immunoblotting analysis to assess the levels of autophagic lipidation (LC3B-II) and degradation (SQSTM1). GAPDH and β-actin were used as loading controls. The protein expression density of blots was quantitated by at least three replicates (error bars represent mean ± SE; *, *p*≦0.05; **, *p*≦0.01; ***, *p*≦0.001).

**Supplementary Figure 10**

**Supplementary Figure 10. Statistical protein expression analysis of Figure 3F-3H.**

MDA-MB-231 cells expressing *pEGFP-LC3B^pepABLuc^* were subjected to pre-treatment with or without 10 μM MG132, followed by 4 hours with (Figure 3F) EBSS and (Figure 3G) serum starvation treatment. The lysates were collected and immunoblotted with anti-luciferase antibody. (Figure 3H) The level of cleaved luciferase and autophagy degradation was evaluated by immunoblotting in MDA-MB-231 cells expressing *pEGFP-LC3B^pepABLuc^*, treated with EBSS in the presence or absence of 30 μM CQ for 24 hours. GAPDH was used as a loading control. The protein expression density of blots was quantitated by at least three replicates (error bars represent mean ± SE; *, *p*≦0.05; **, *p*≦ 0.01; ***, p≦0.001; N.S., not significant).

**Supplementary Figure 11**

**Supplementary Figure 11. Statistical protein expression analysis of Figure 4H&5D.**

MDA-MB-231 cells were treated with 1 to 100 μM RSV for 24 hours, with or without 30 μM CQ co-treatment. Cell lysates were collected, and the accumulation of LC3B-II and SQSTM1 was examined by immunoblotting. (Figure 5D) MDA-MB-231 cells were treated with DOX (0.5 μM) with or without RSV (50 to 100 μM) for 48 hours. The activity of caspase3 and PARP cleavage was examined by Immunoblot. β-actin was used as a loading control. The protein expression density of blots was quantitated by at least three replicates (error bars represent mean ± SE; *, *p*≦0.05; ***, *p*≦0.001).

**Supplementary Figure 12**

**Supplementary Figure 12. Statistical protein expression analysis of Supplementary Figure 6.**

MDA-MB-231 cells expressing *pEGFP-LC3B^pepABLuc^* were subjected to pre-treatment with or without 100 nM BafA1 autophagy inhibitor, followed by 4 hours with 1 to 100 μM Monensin treatments. The lysates were collected and immunoblotted with anti-luciferase and LC3B antibodies. β-actin was used as a loading control. The protein expression density of blots was quantitated by at least three replicates (error bars represent mean ± SE; *, *p*≦0.05; **, *p*≦0.01; N.S., not significant).
