# Supplementary material for: G-cleave LC3B biosensor: monitoring autophagy and assessing resveratrol's synergistic impact on doxorubicin-induced apoptosis in breast cancer cells
Source: Breast Cancer Res. 2024 Dec 30;26:190. doi: 10.1186/s13058-024-01951-1 (PMC11687128; doi:10.1186/s13058-024-01951-1)
Supplement: Supplementary file 3 — Supplementary material 3 [file 13058_2024_1951_MOESM3_ESM.docx]

Supplementary information

Figure 1C

EGFP


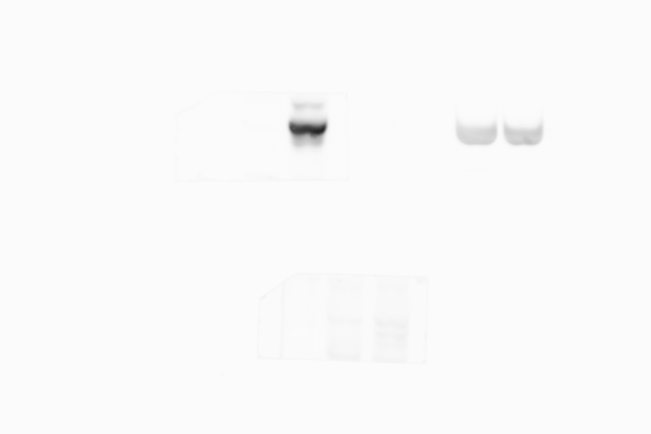


pEGFP-LC3B^pepABLuc^

MDA-MB-231

- +

luciferase


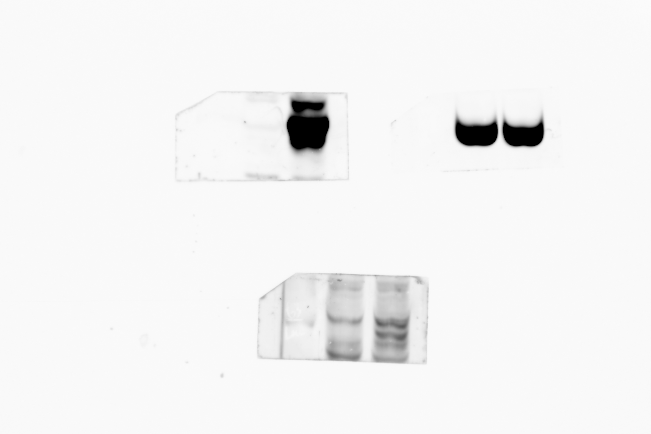


- +

pEGFP-LC3B^pepABLuc^

MDA-MB-231

β-actin


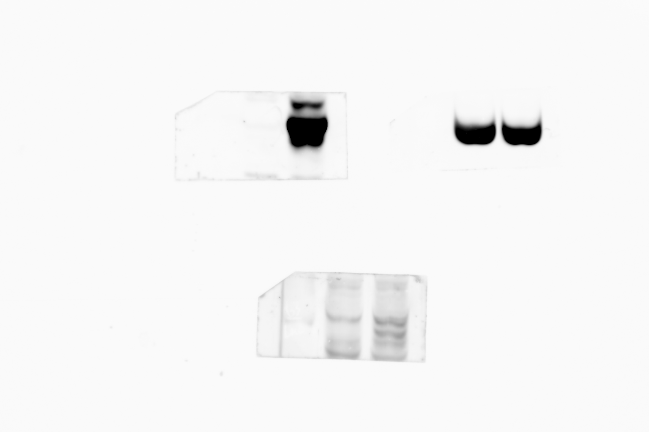


- +

MDA-MB-231

pEGFP-LC3B^pepABLuc^

Figure 1F

EGFP_(n1)


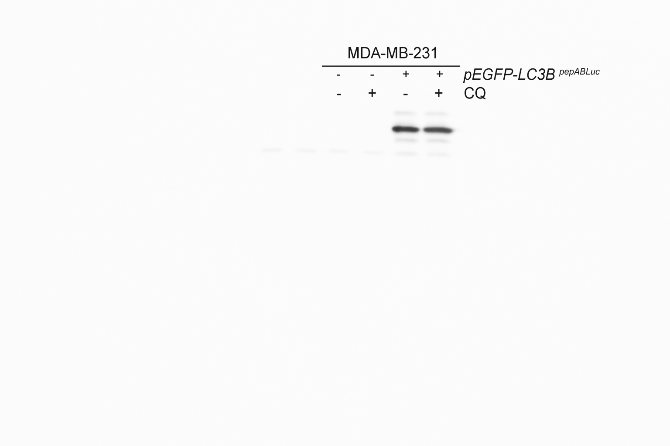


Luciferase_(n1)


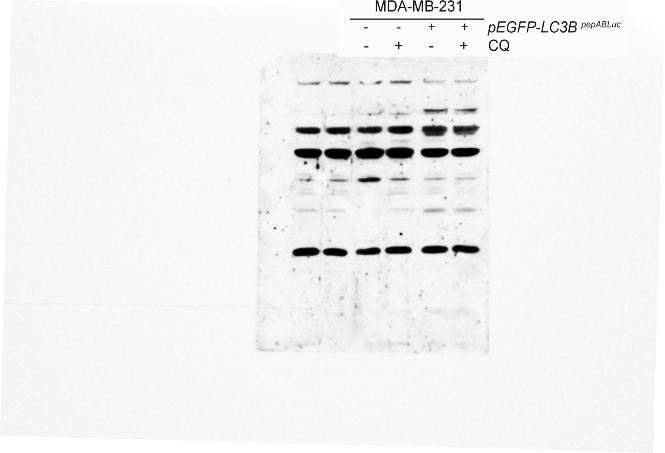


LC3_(n1)


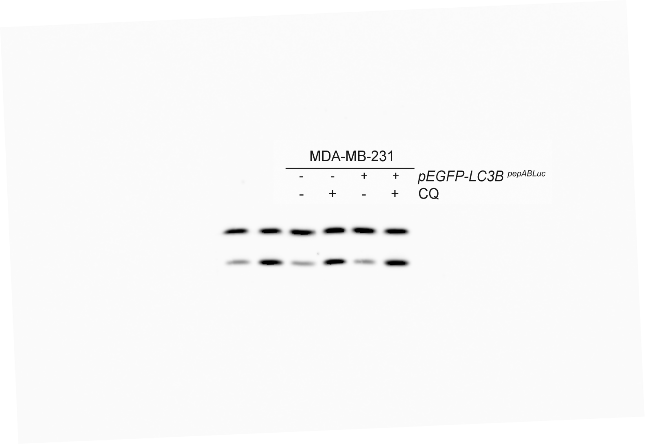


SQSTM1_(n1)


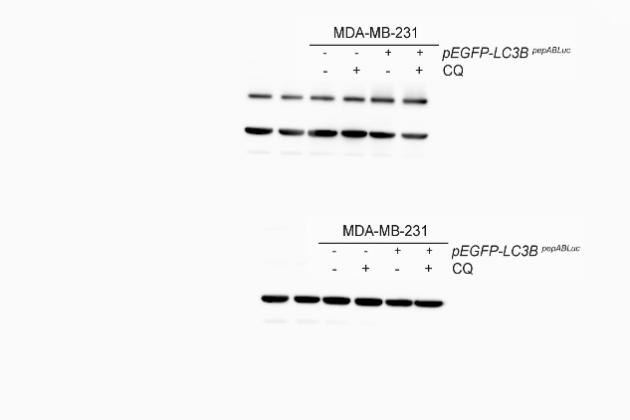


β-actin_(n1)


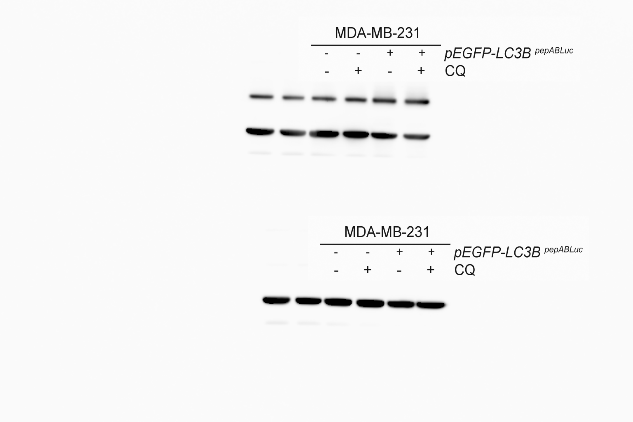


EGFP_(n2)


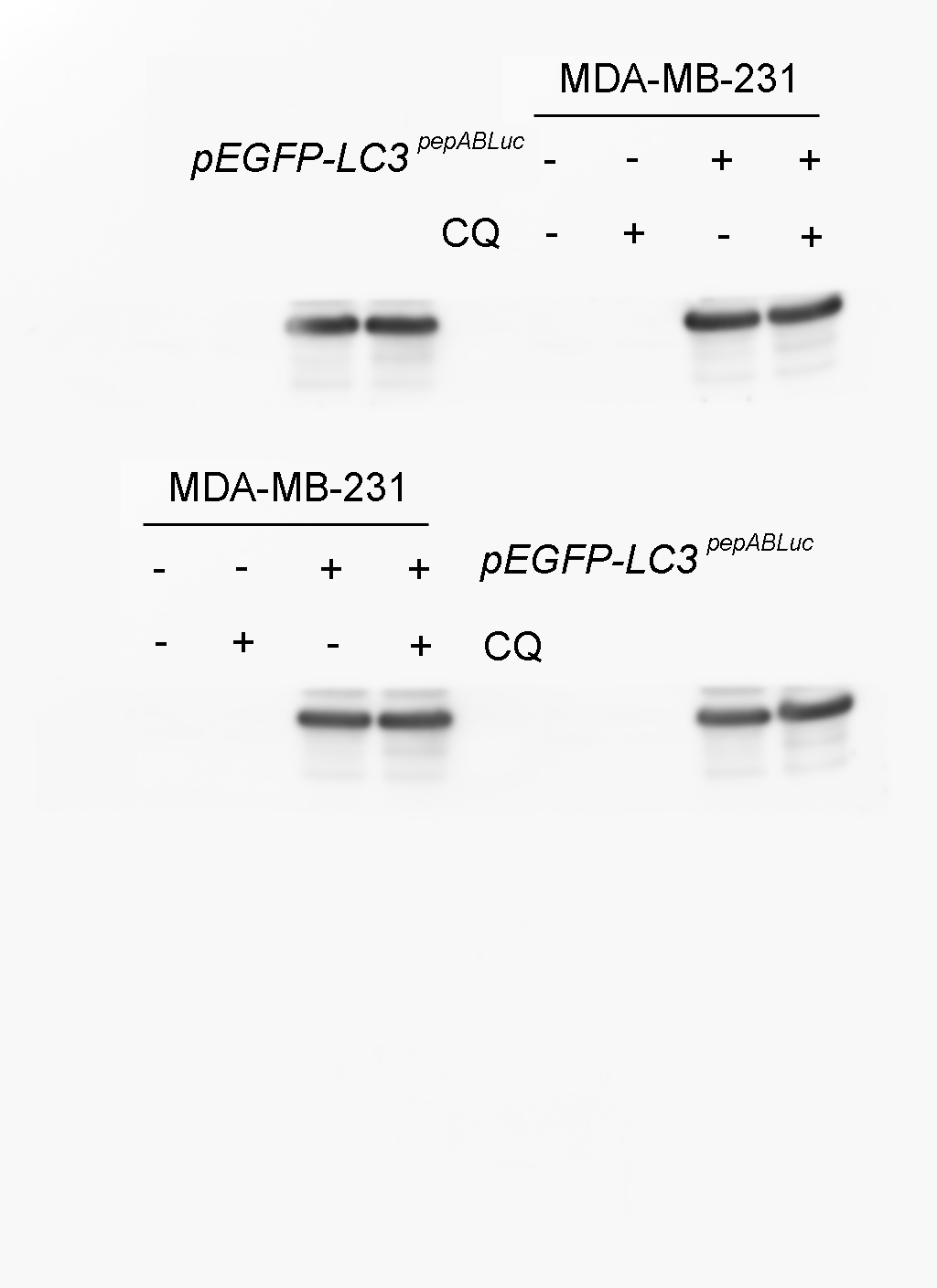


Luciferase_(n2)


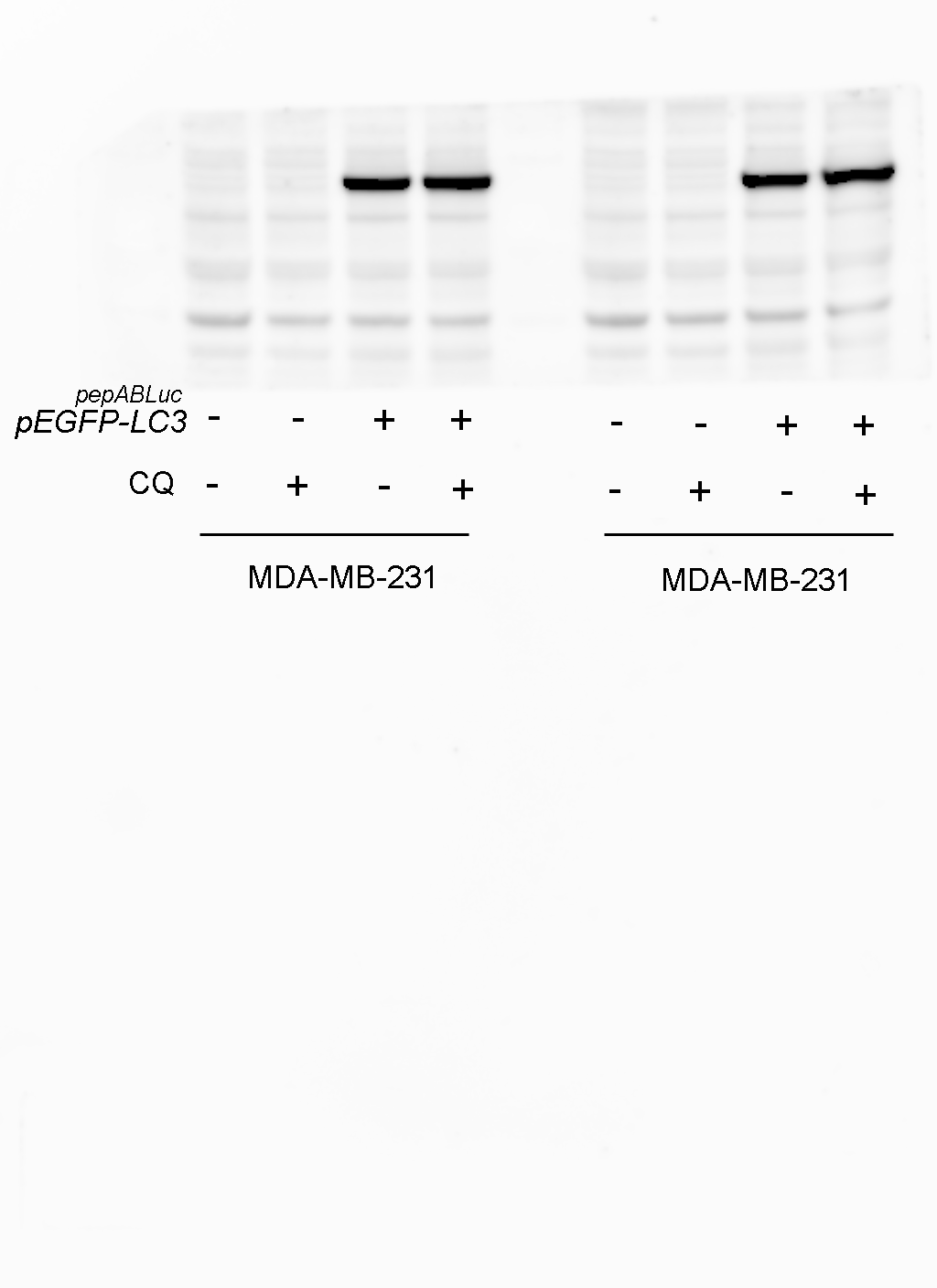


LC3_(n2)


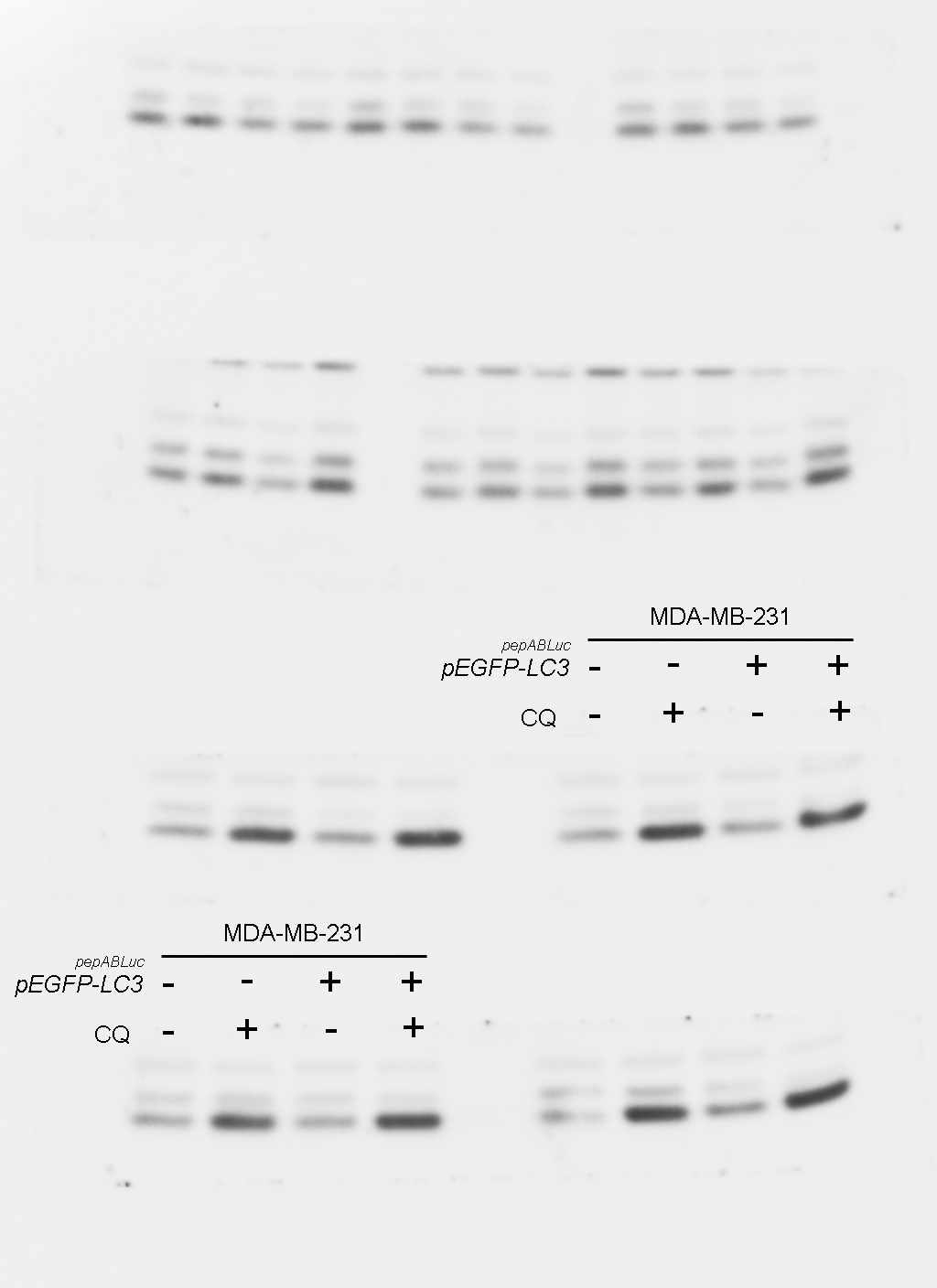


SQSTM1_(n2)


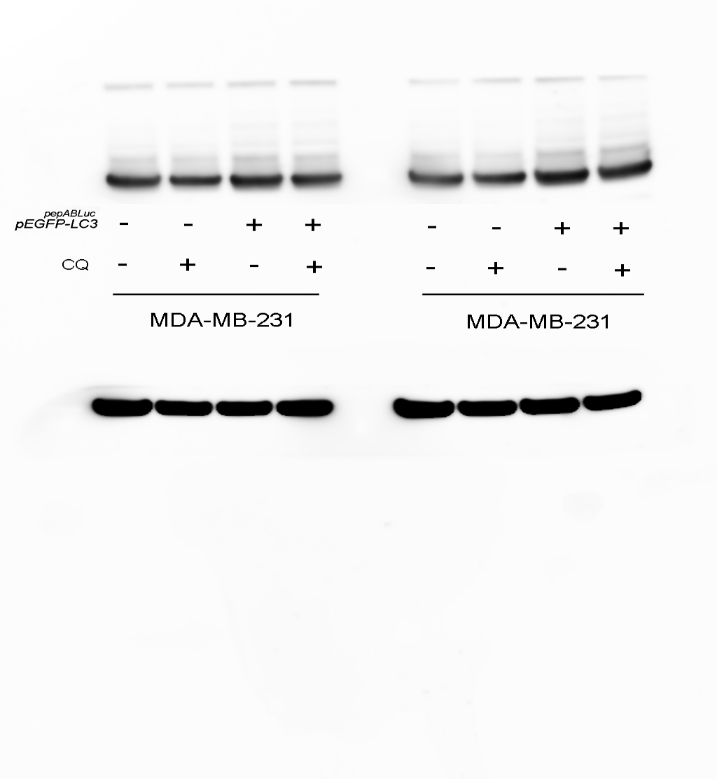


β-actin_(n2)


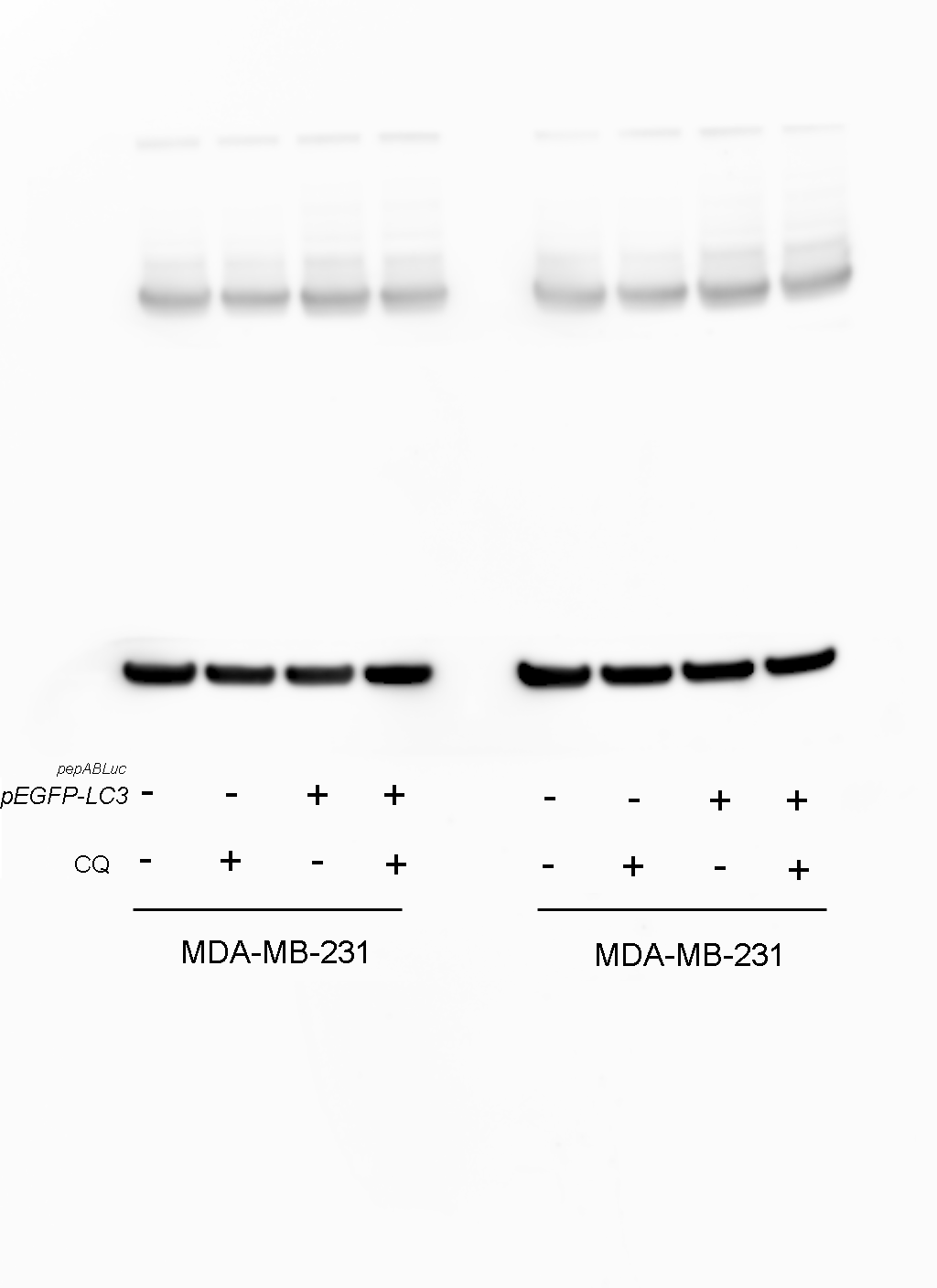


EGFP_(n3)


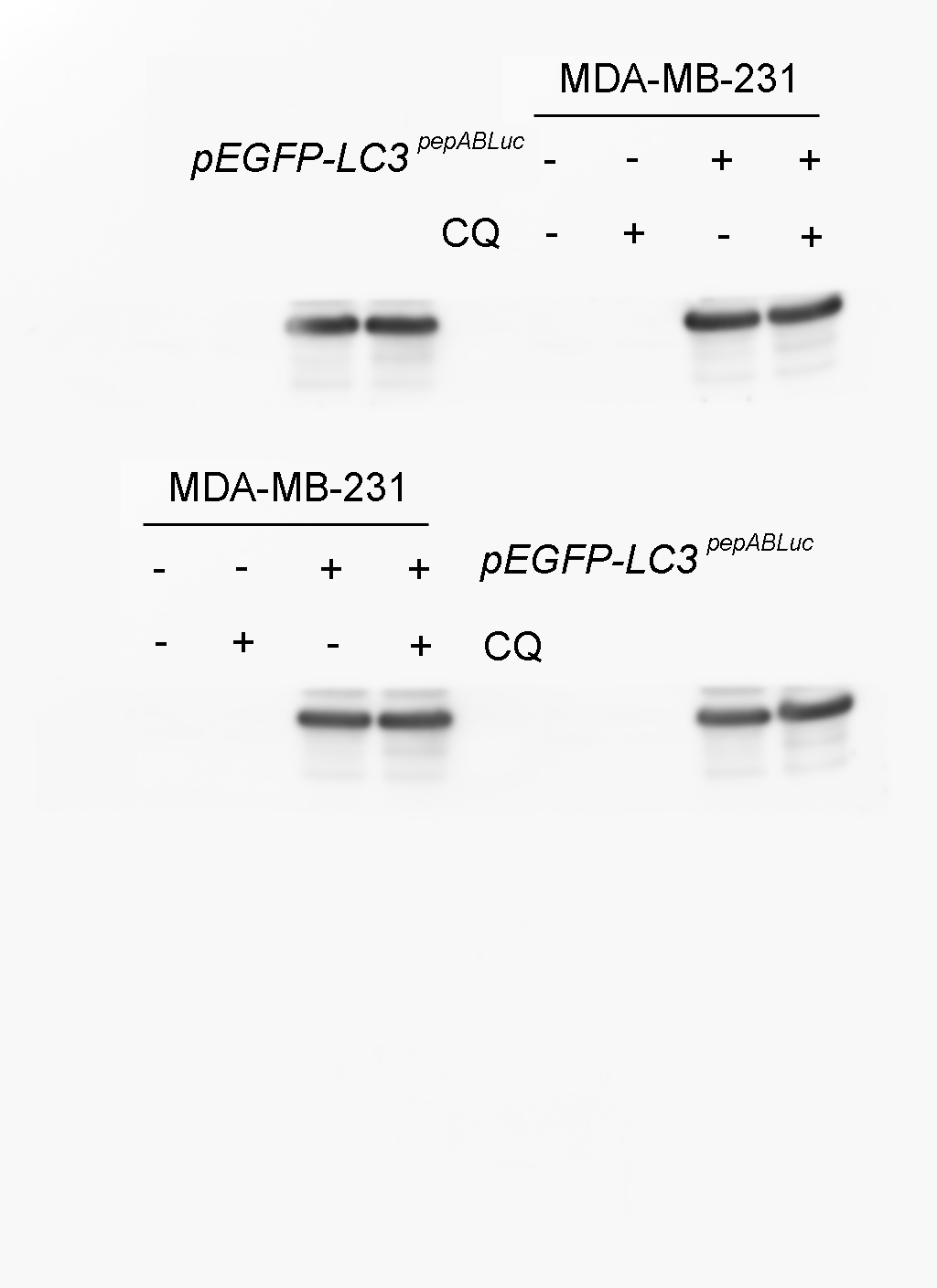


Luciferase_(n3)


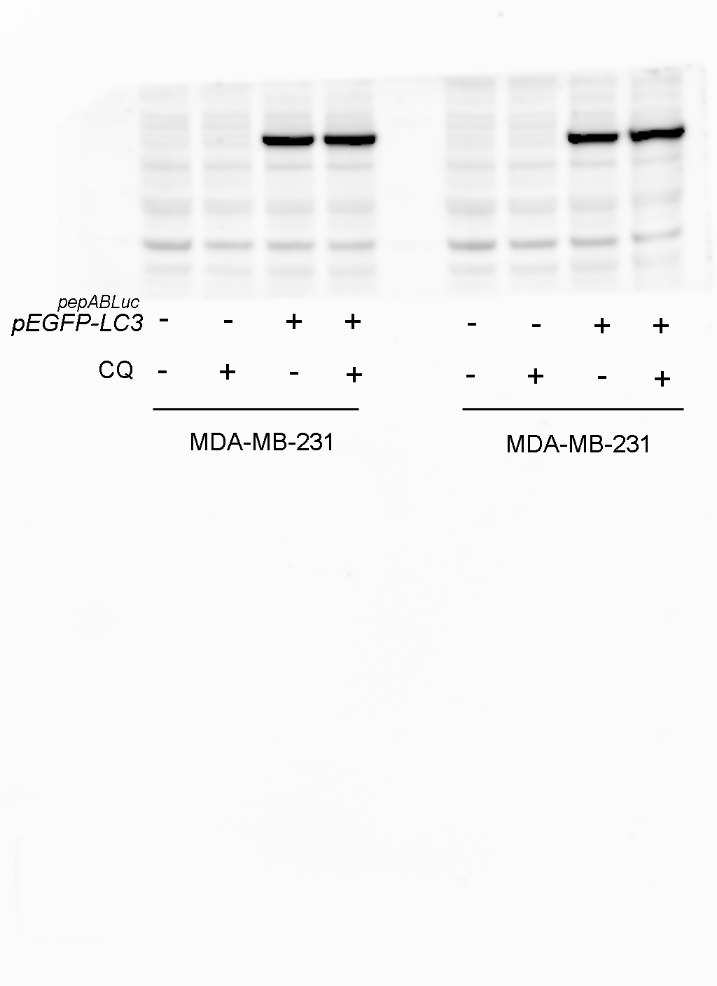


LC3_(n3)


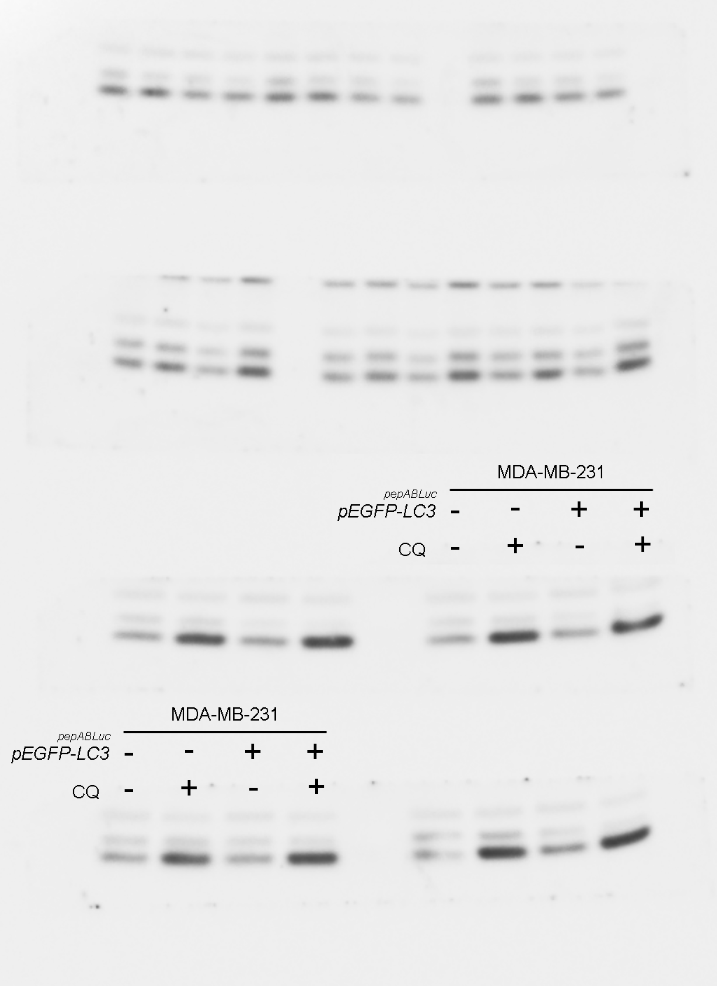


SQSTM1_(n3)


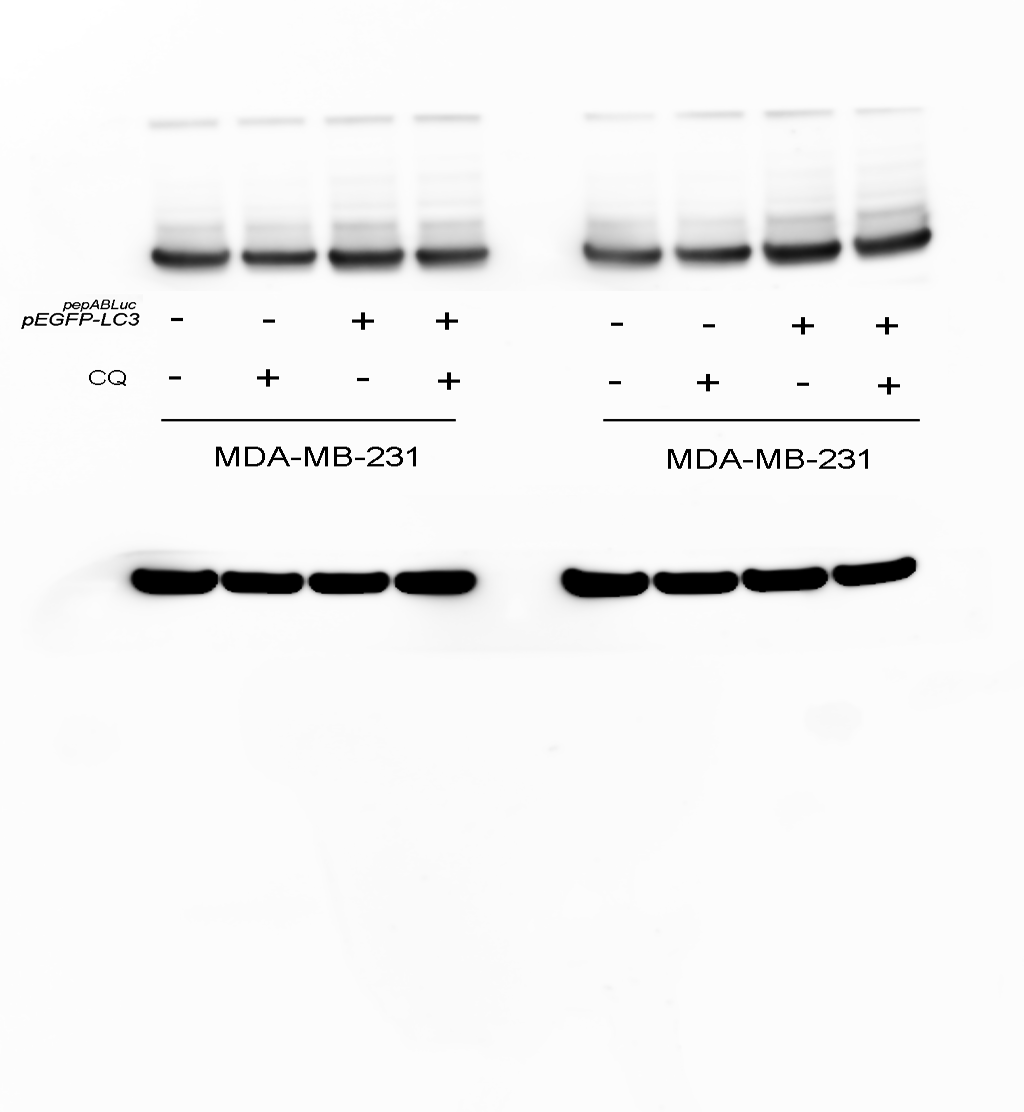


β-actin_(n3)


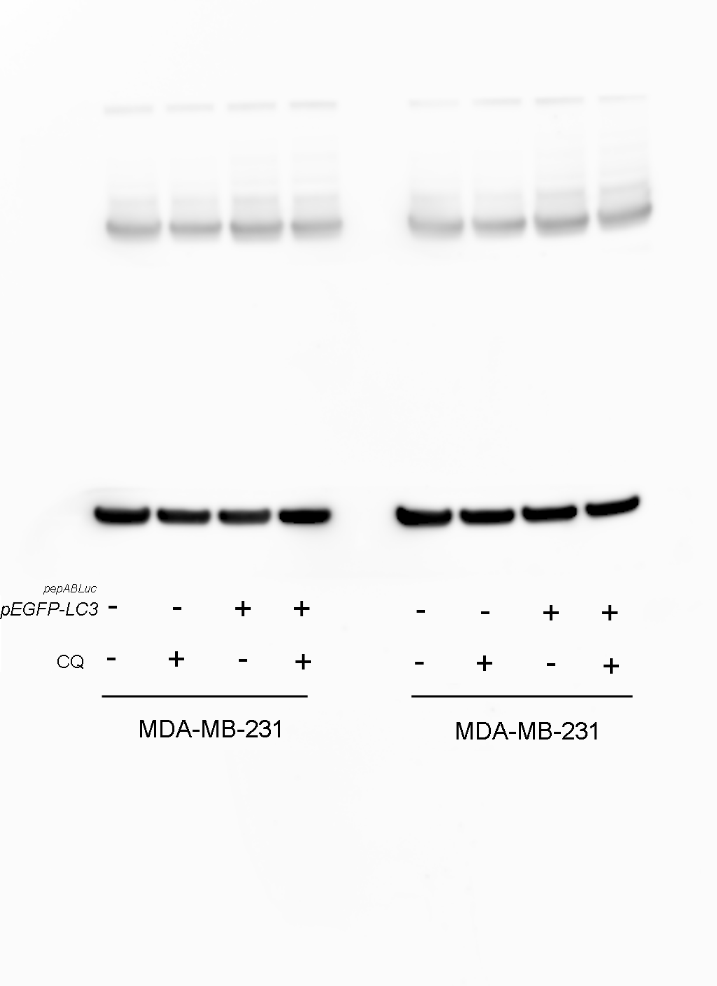


Figure 2A

SQSTM1_(n1)


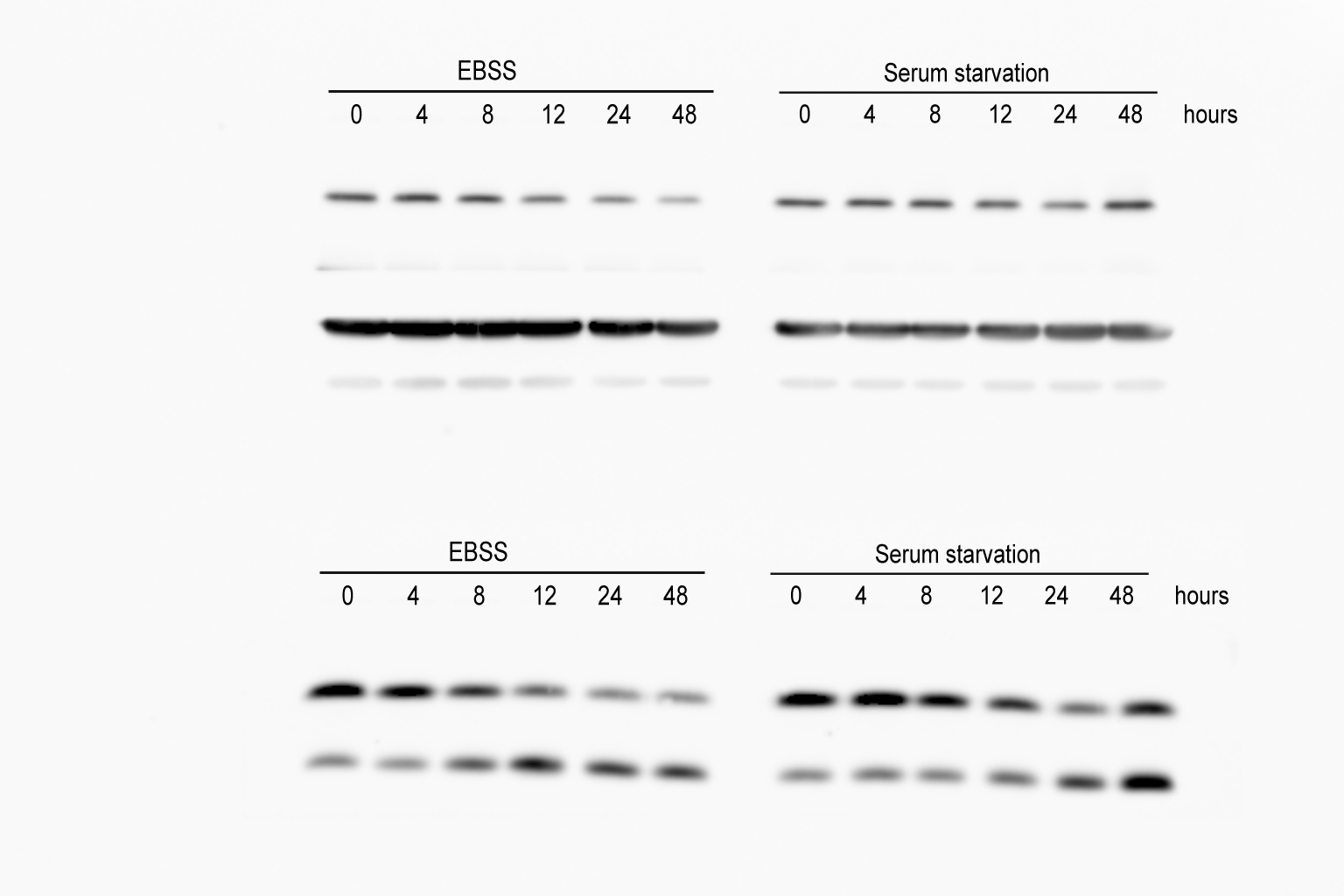


LC3_(n1)


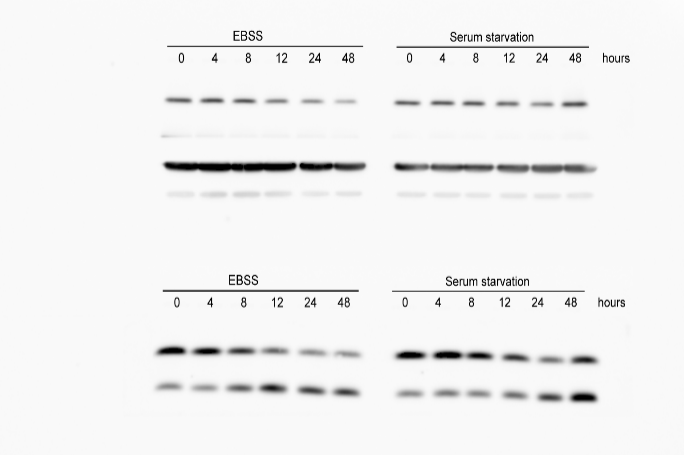


GAPDH_(n1)


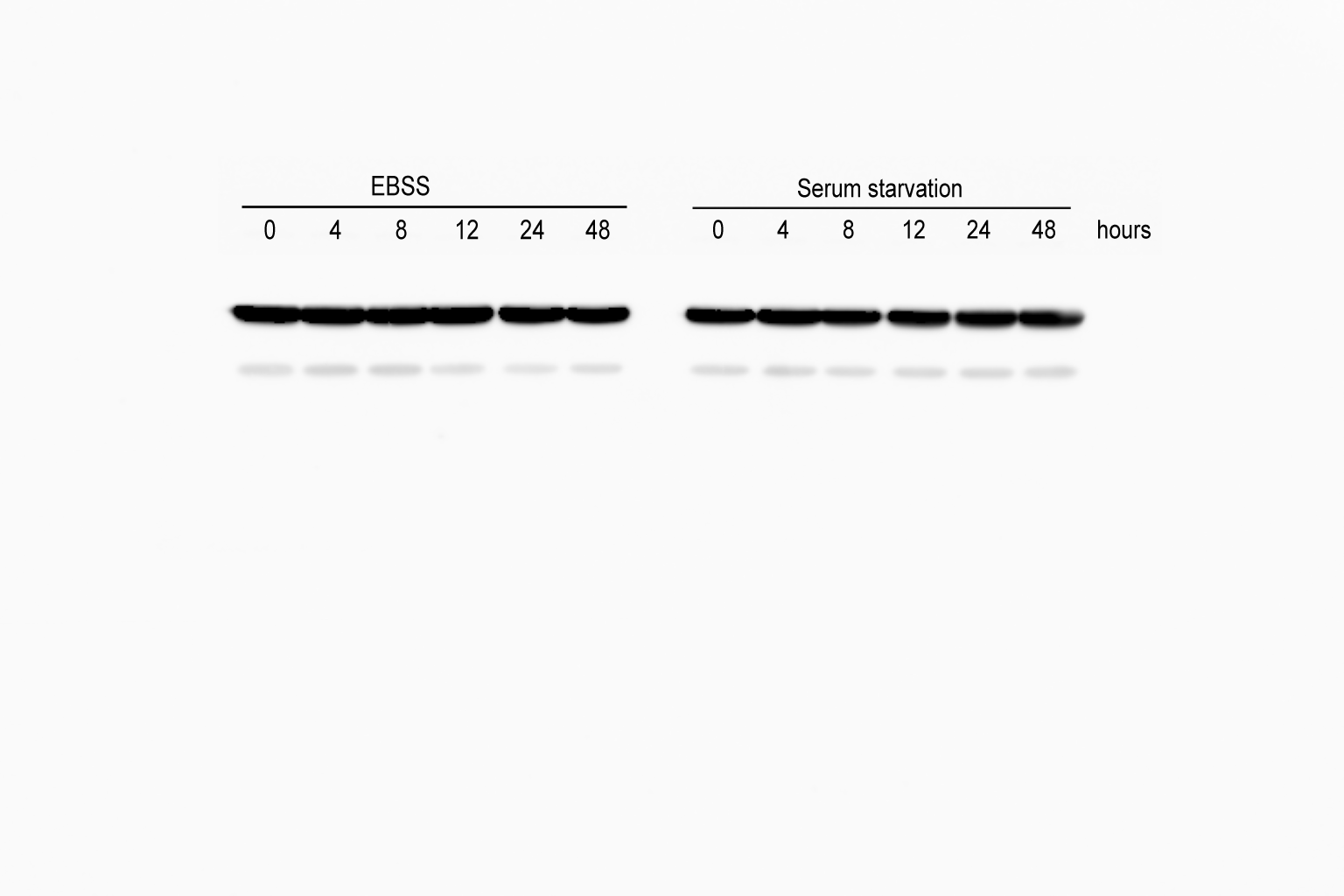


SQSTM1_(n2)


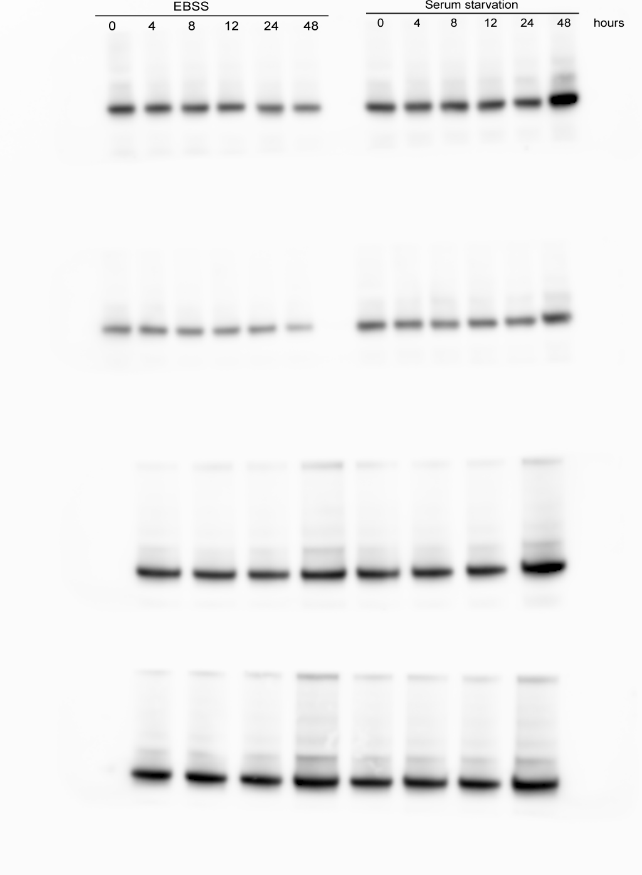


LC3_(n2)


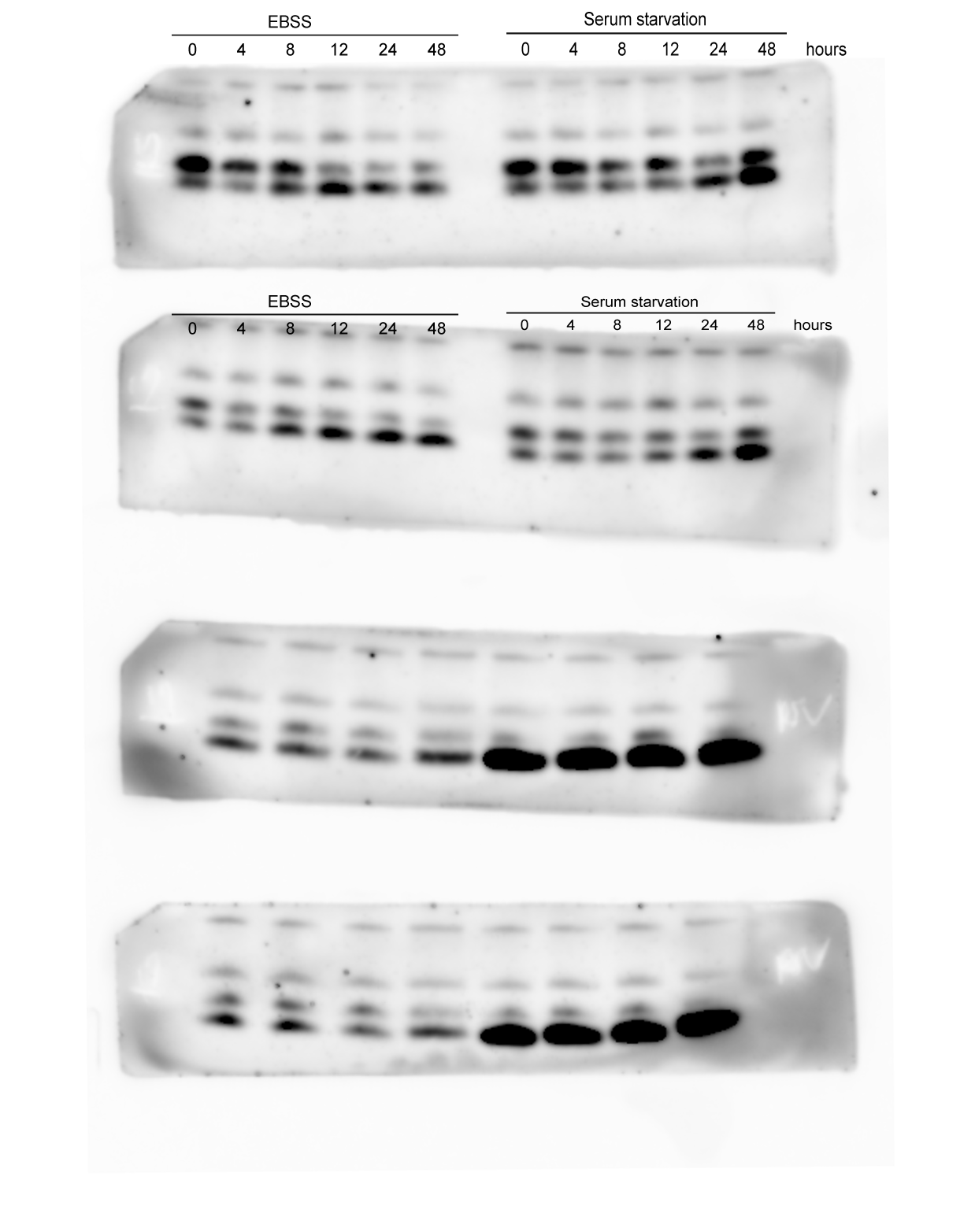


GAPDH_(n2)


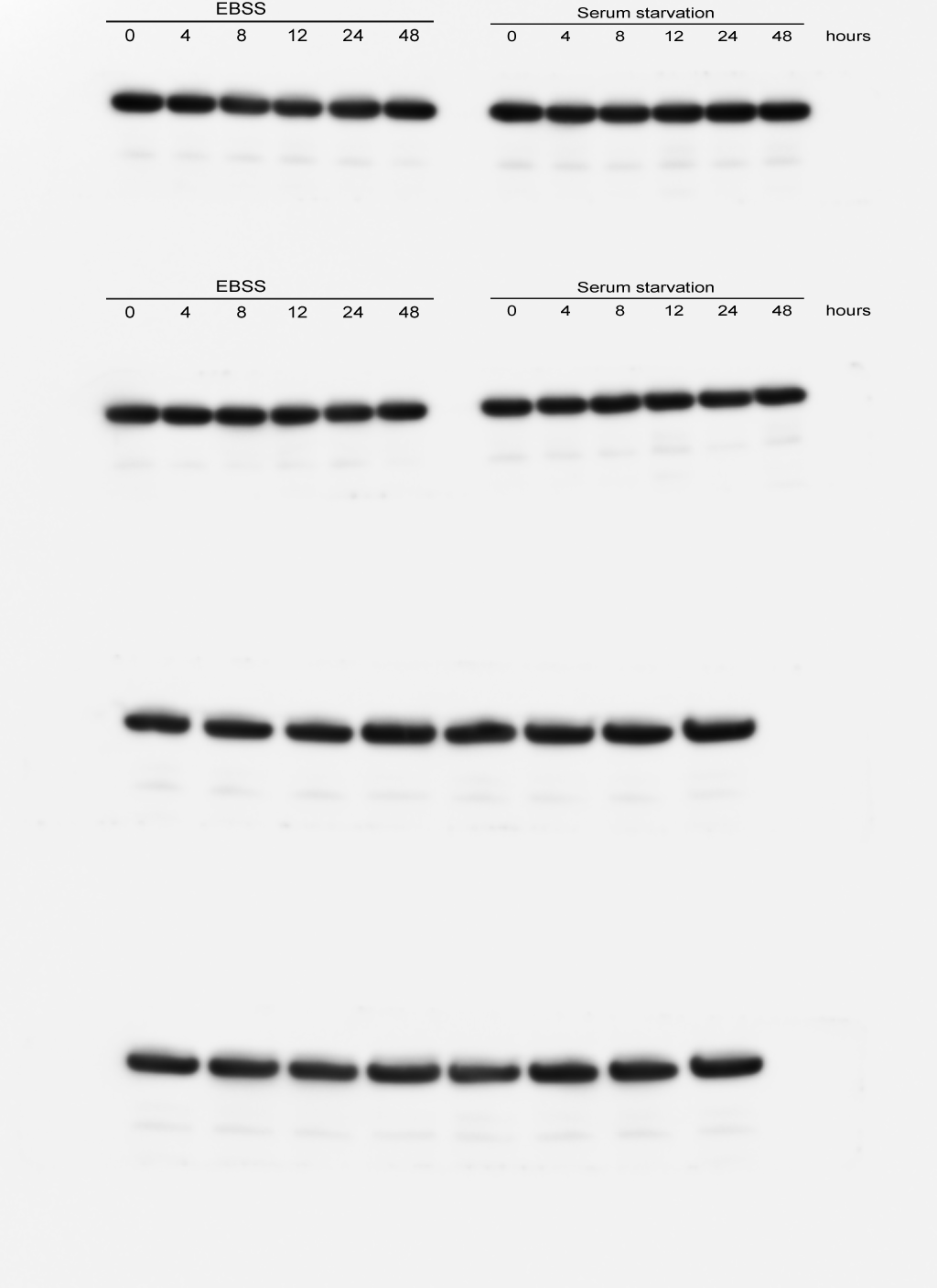


SQSTM1_(n3)


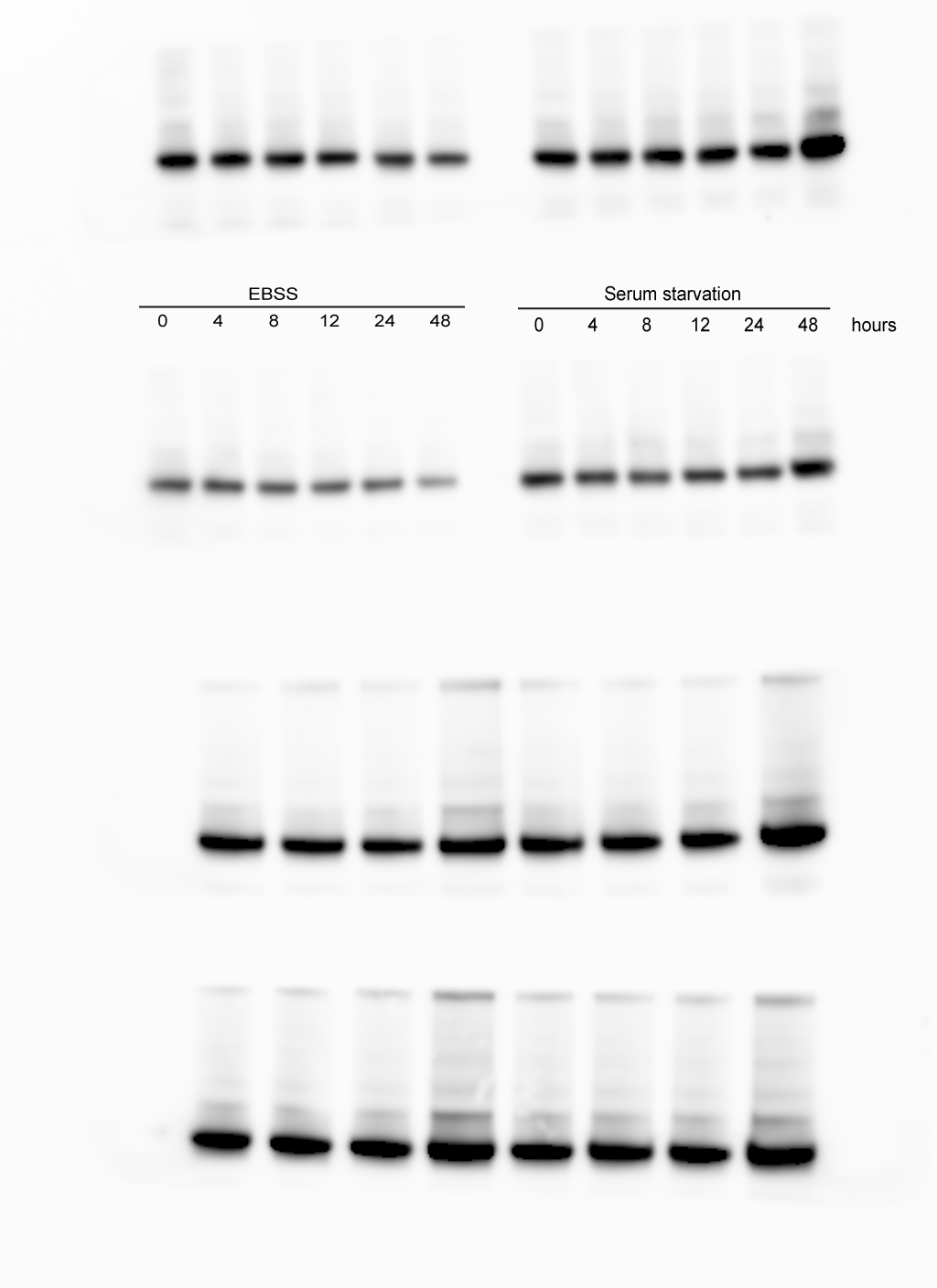


LC3_(n3)


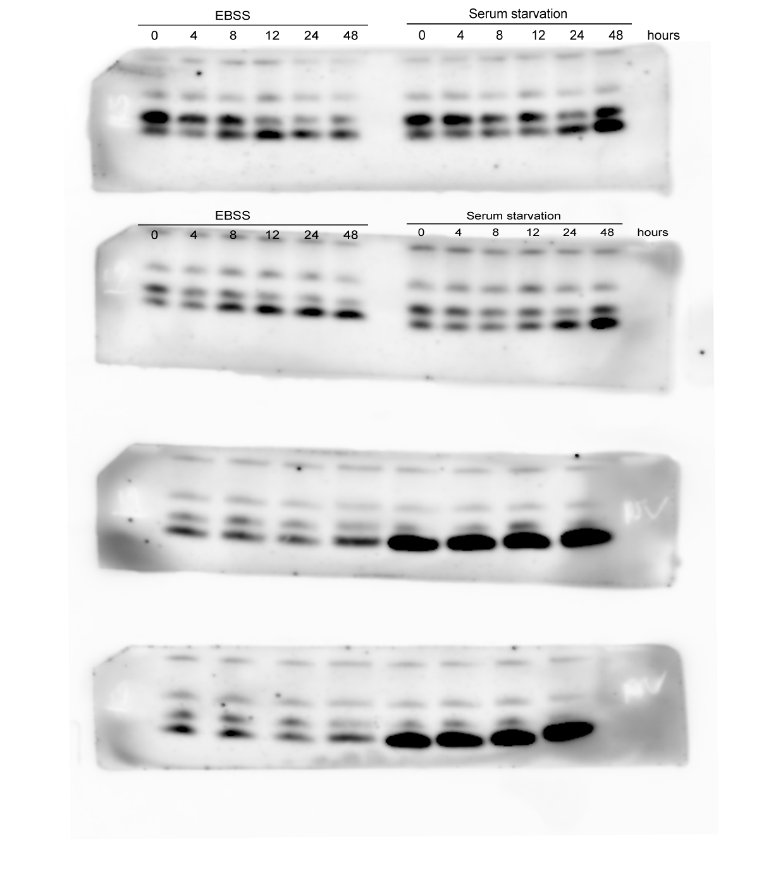


GAPDH_(n3)


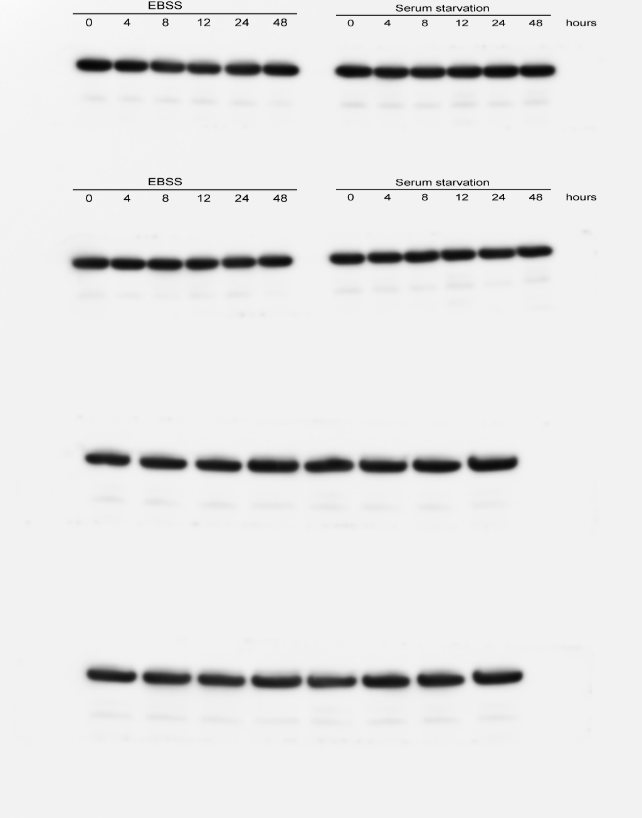


Figure 2B_EBSS

SQSTM1_(n1)


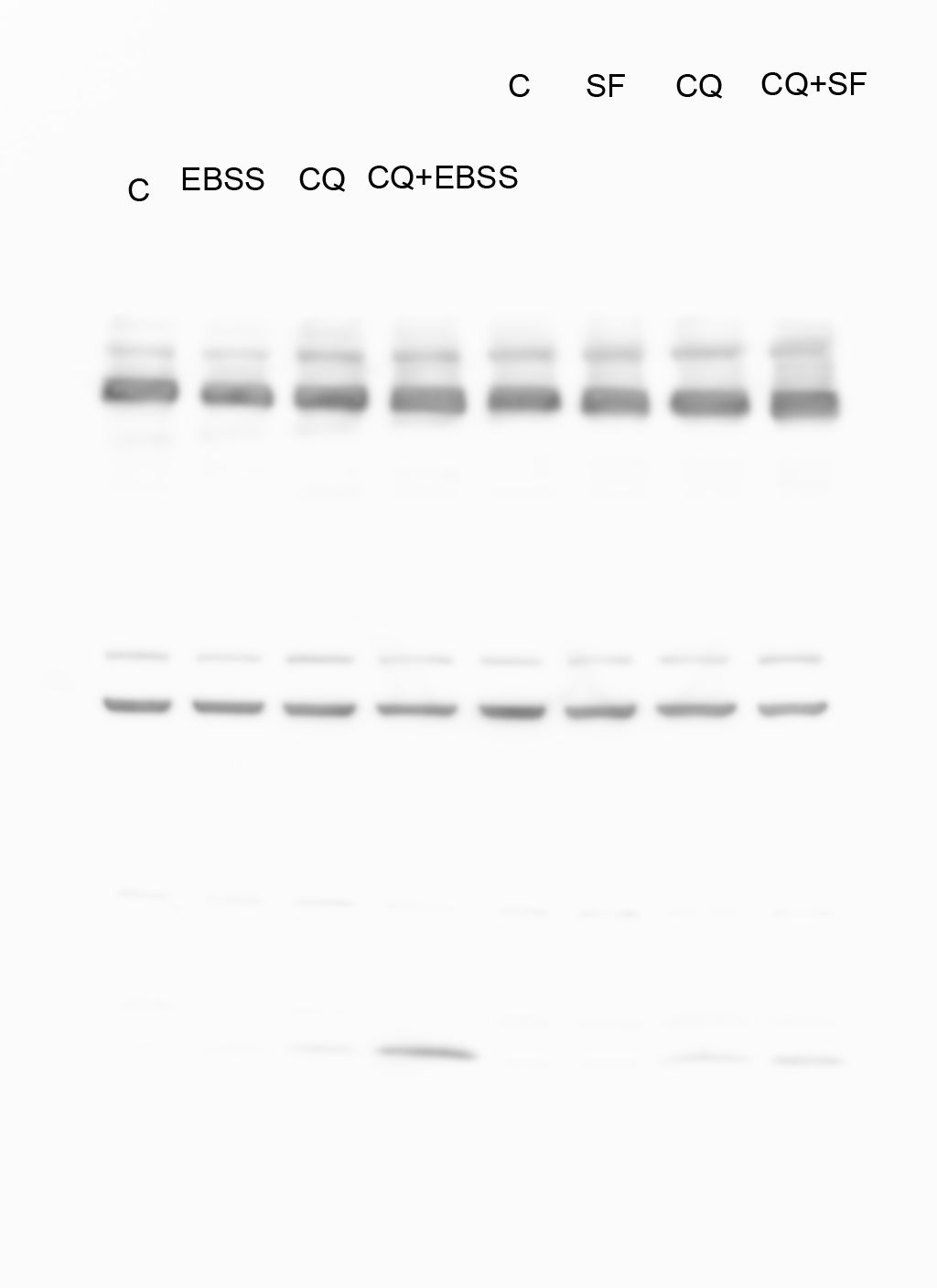


LC3_(n1)


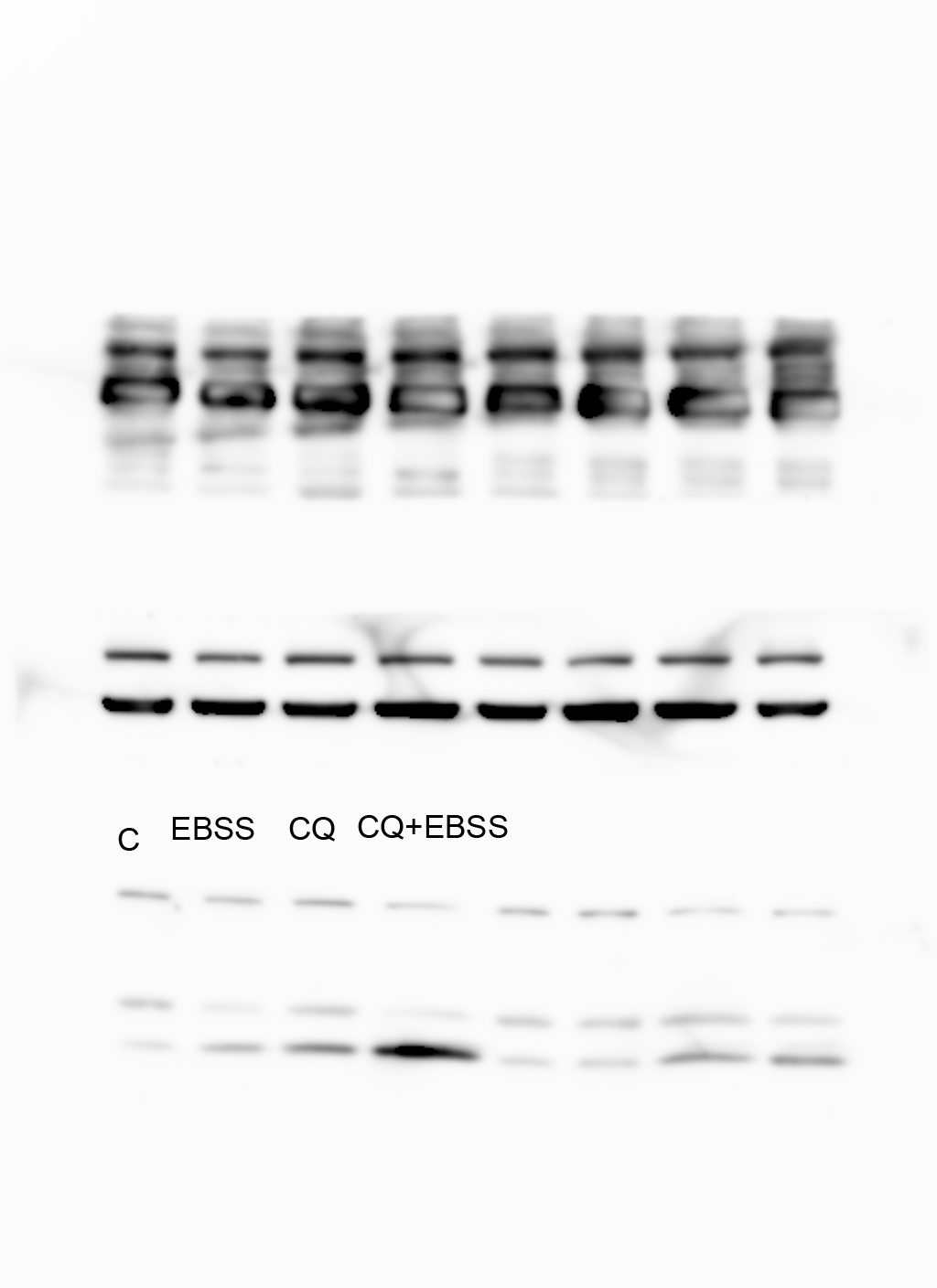


β-actin_(n1)


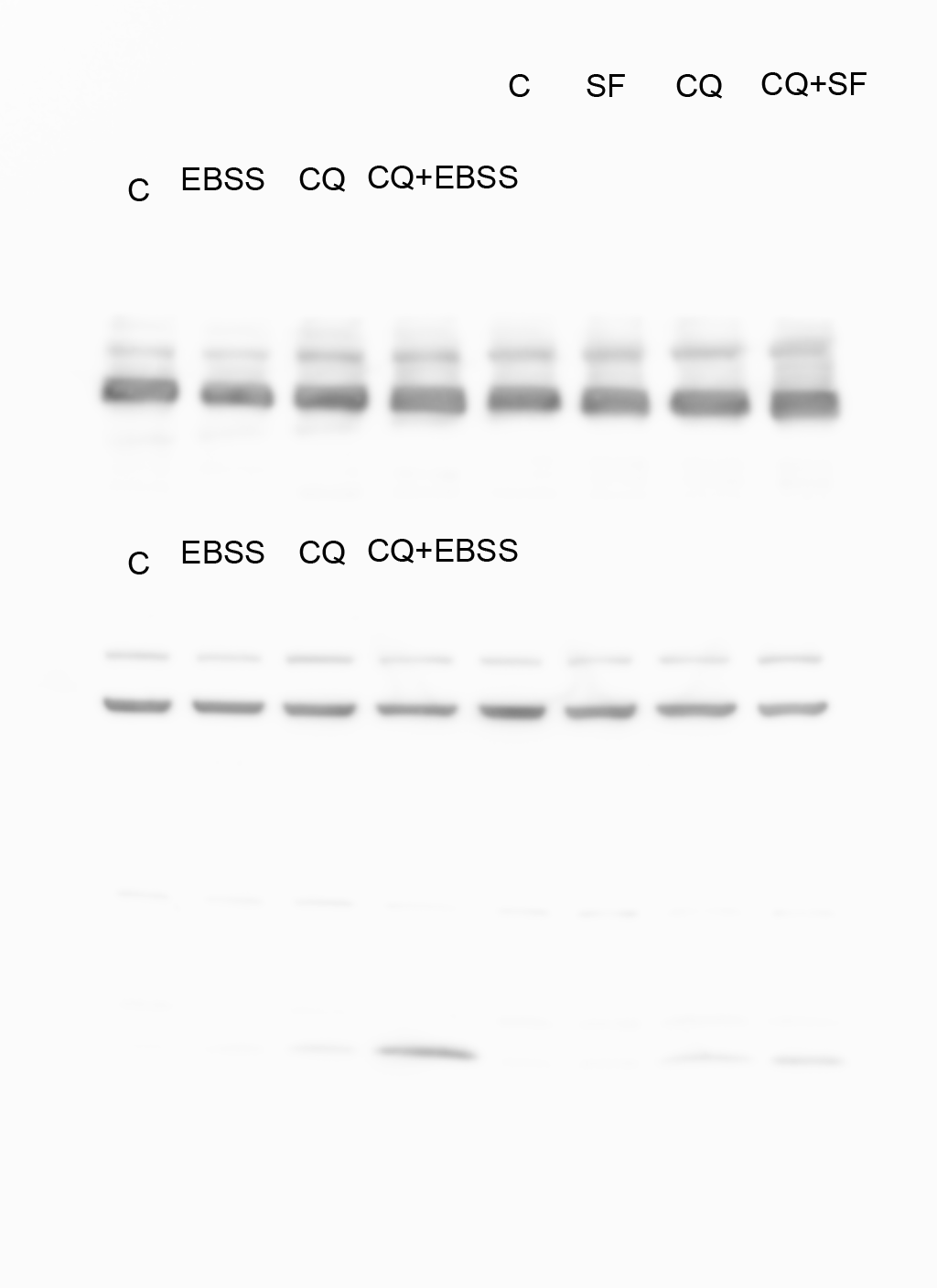


SQSTM1_(n2)


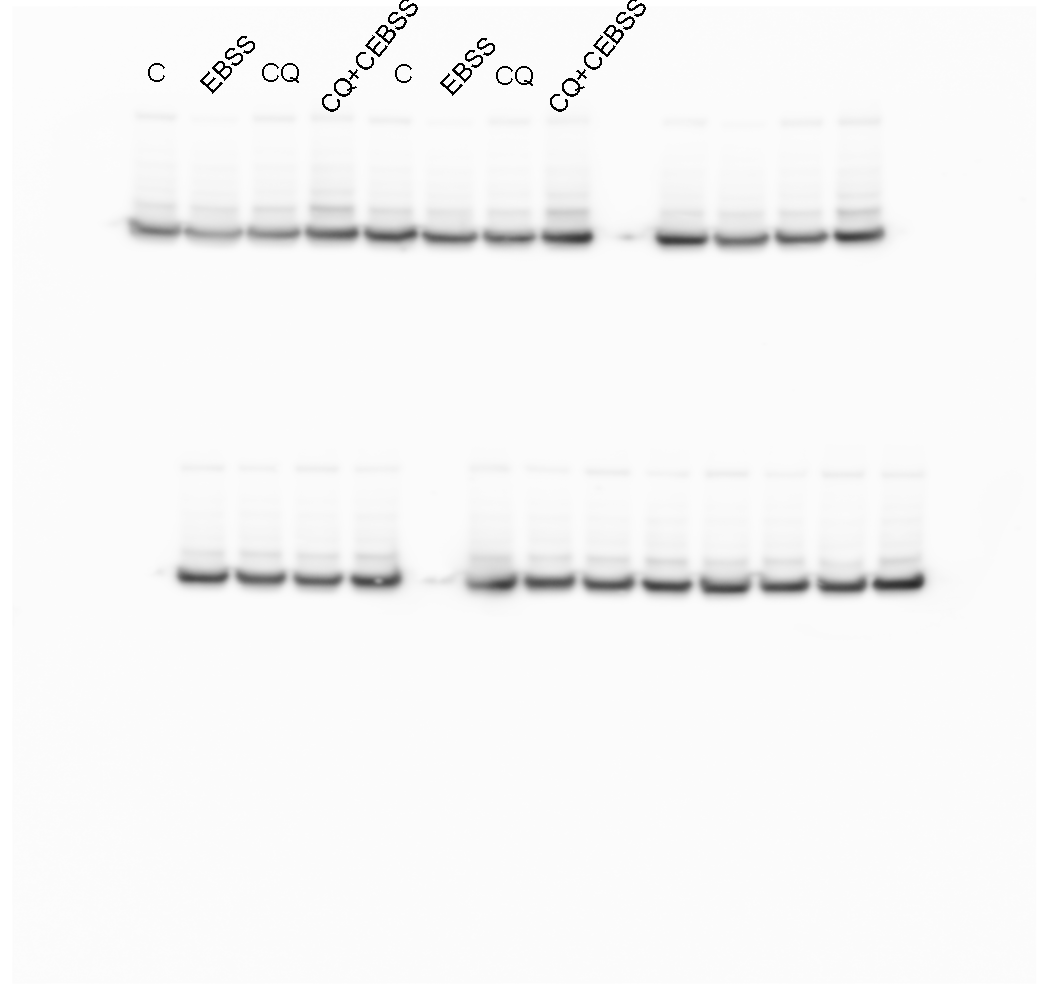


LC3_(n2)


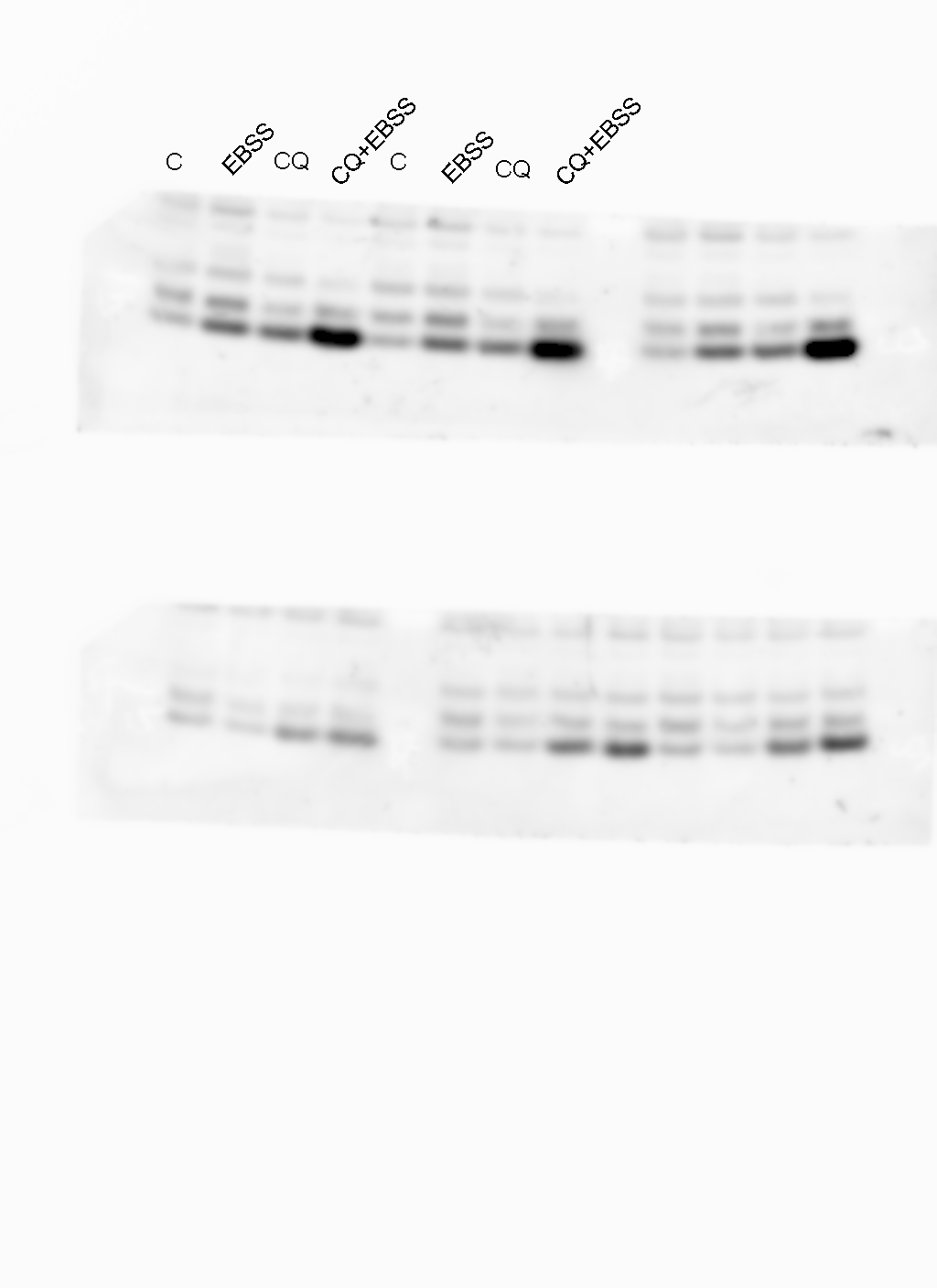


β-actin_(n2)


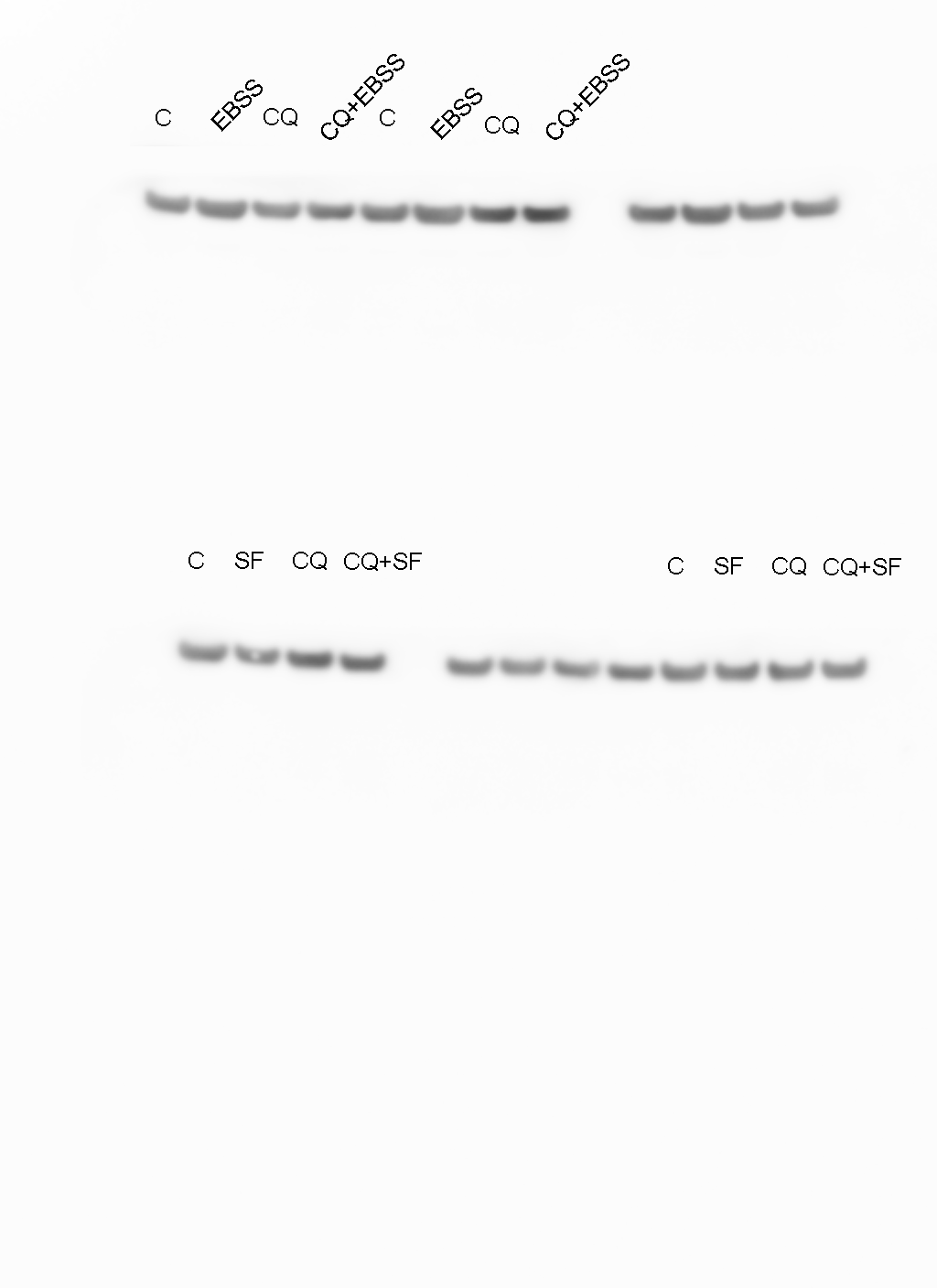


SQSTM1_(n3)


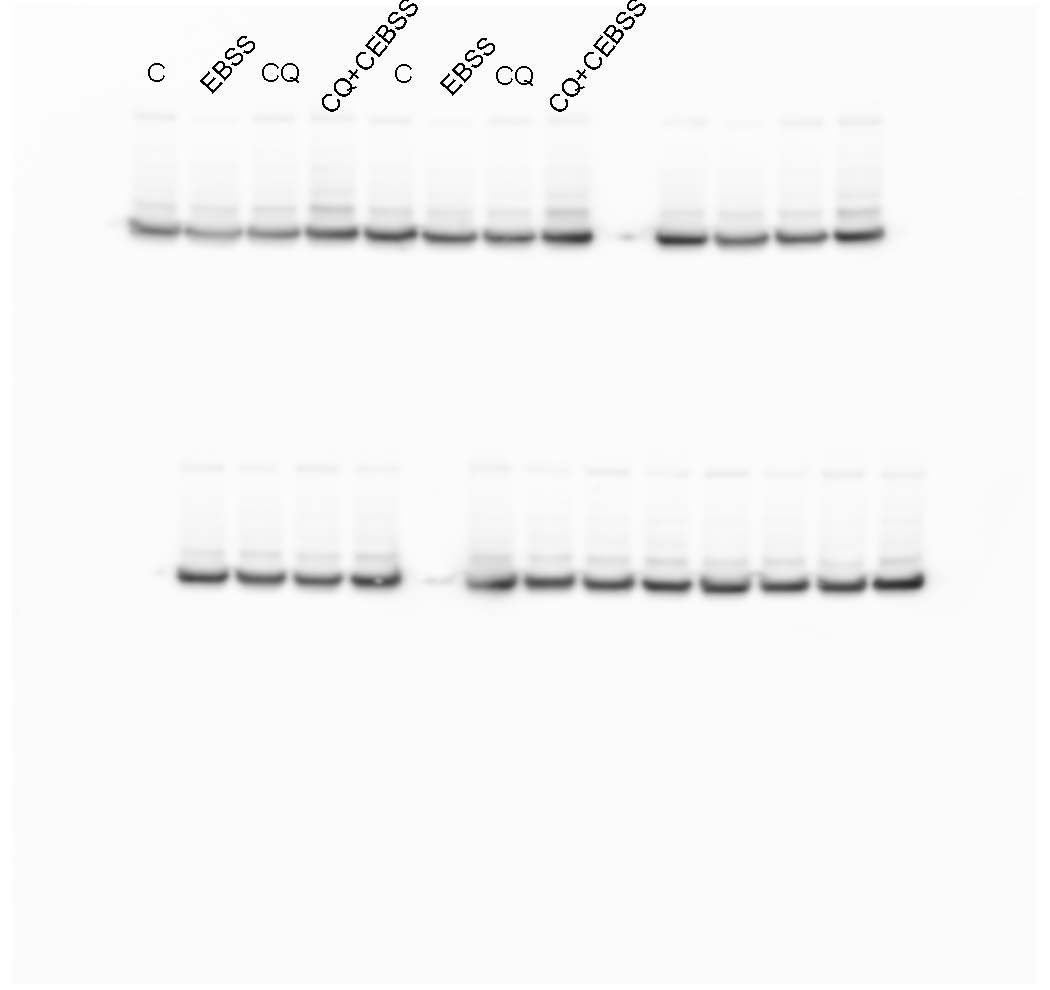


LC3_(n3)


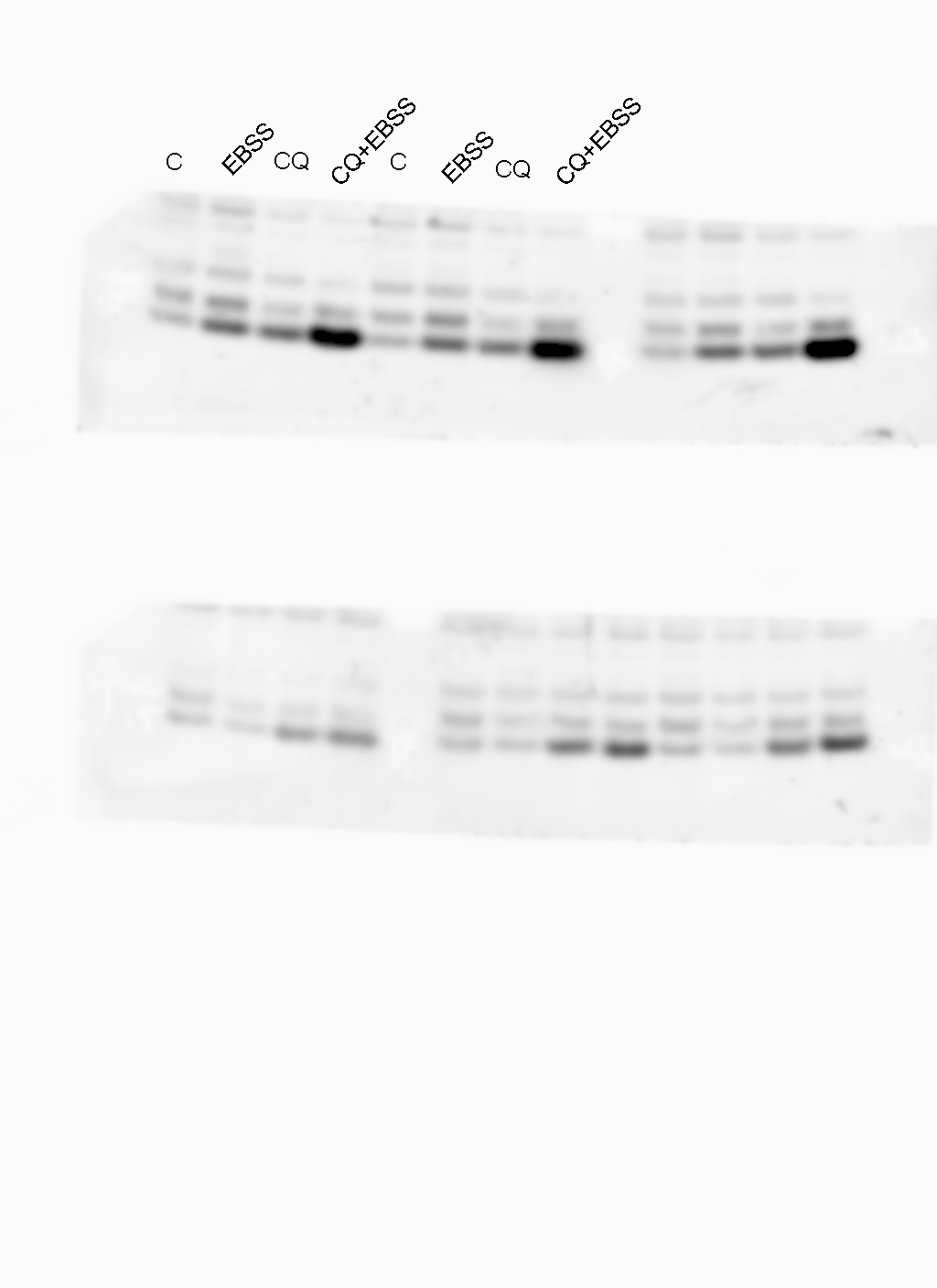


β-actin_(n3)


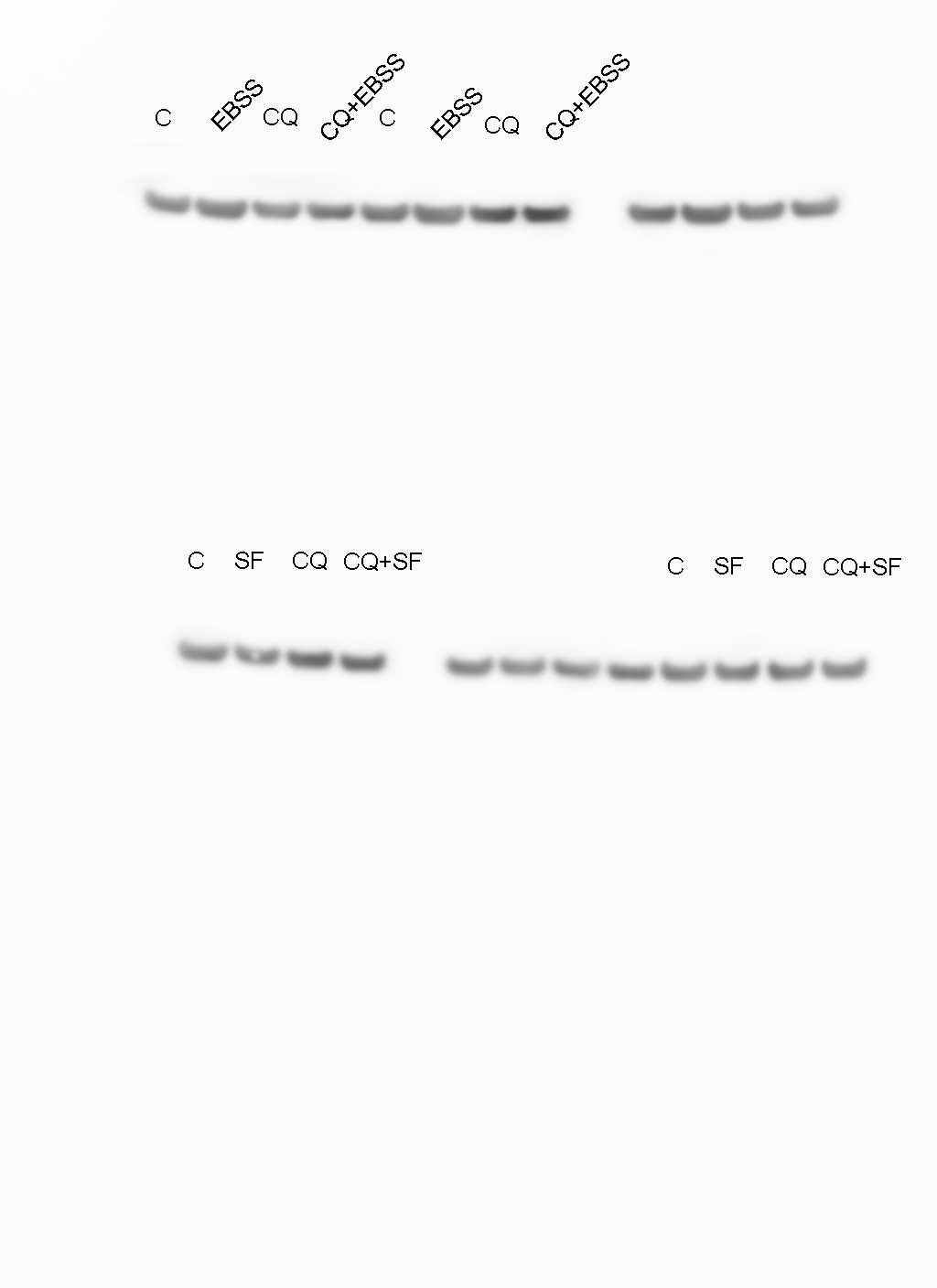


Figure 2B_Serum starvation

SQSTM1_(n1)


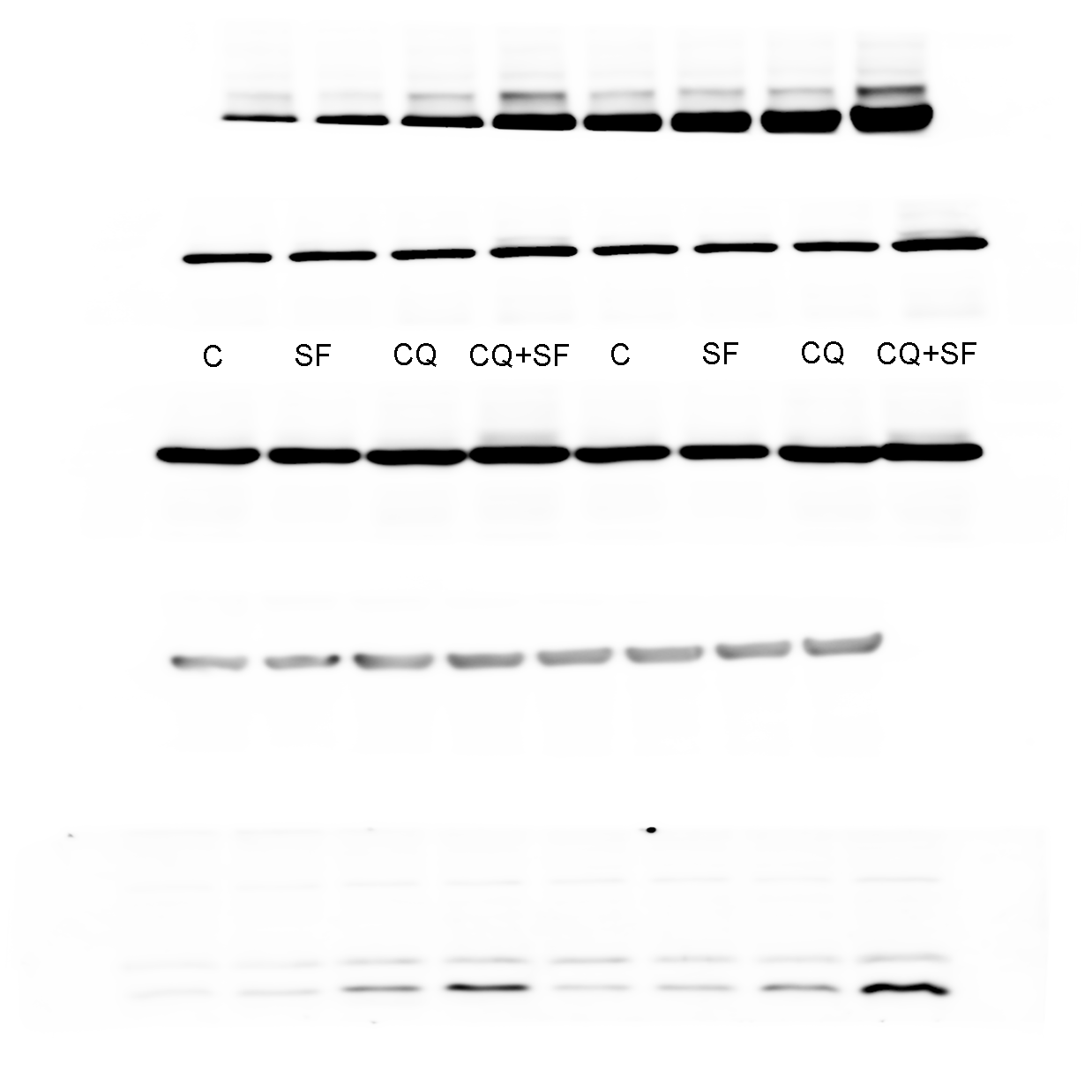


LC3_(n1)


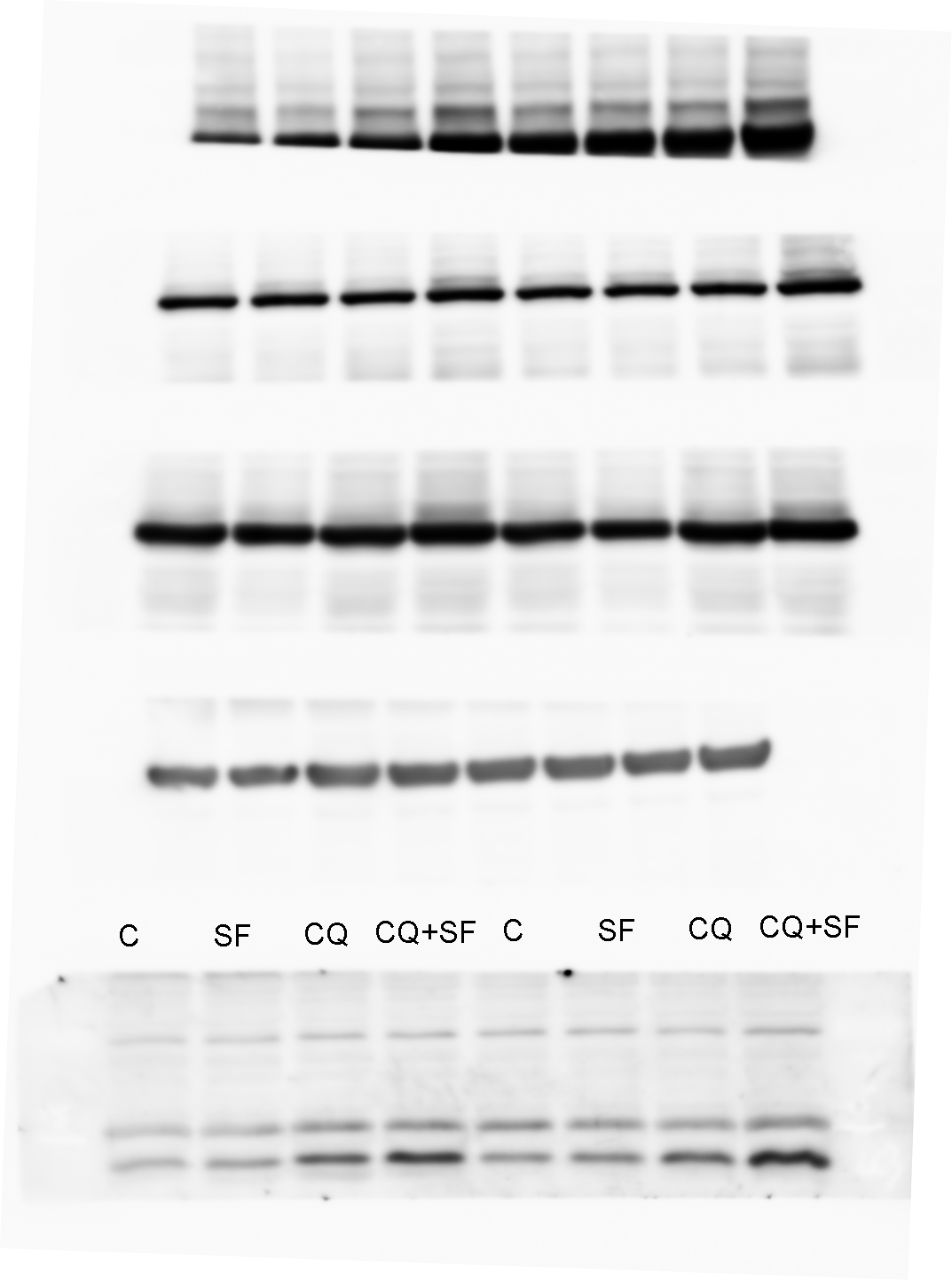


β-actin_(n1)


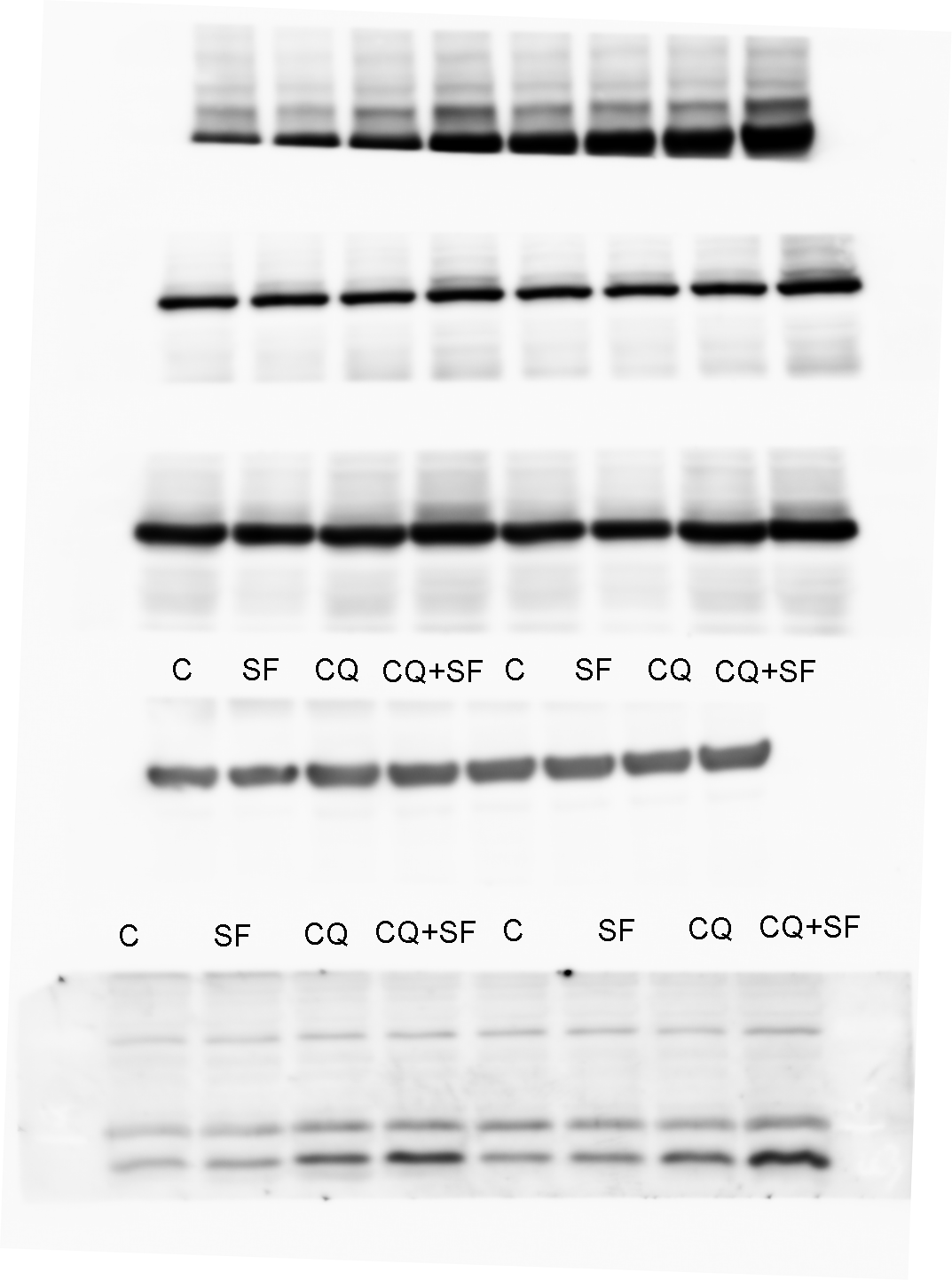


SQSTM1_(n2)


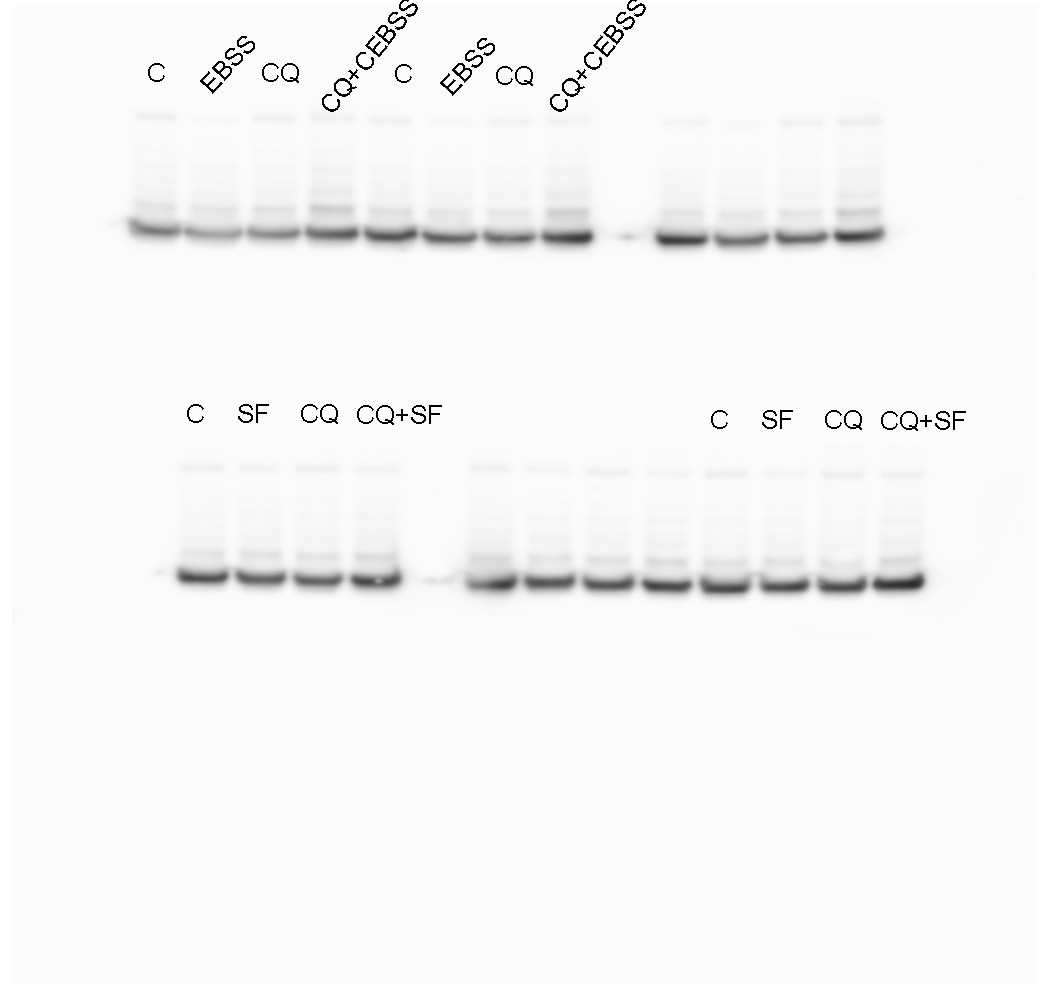


LC3_(n2)


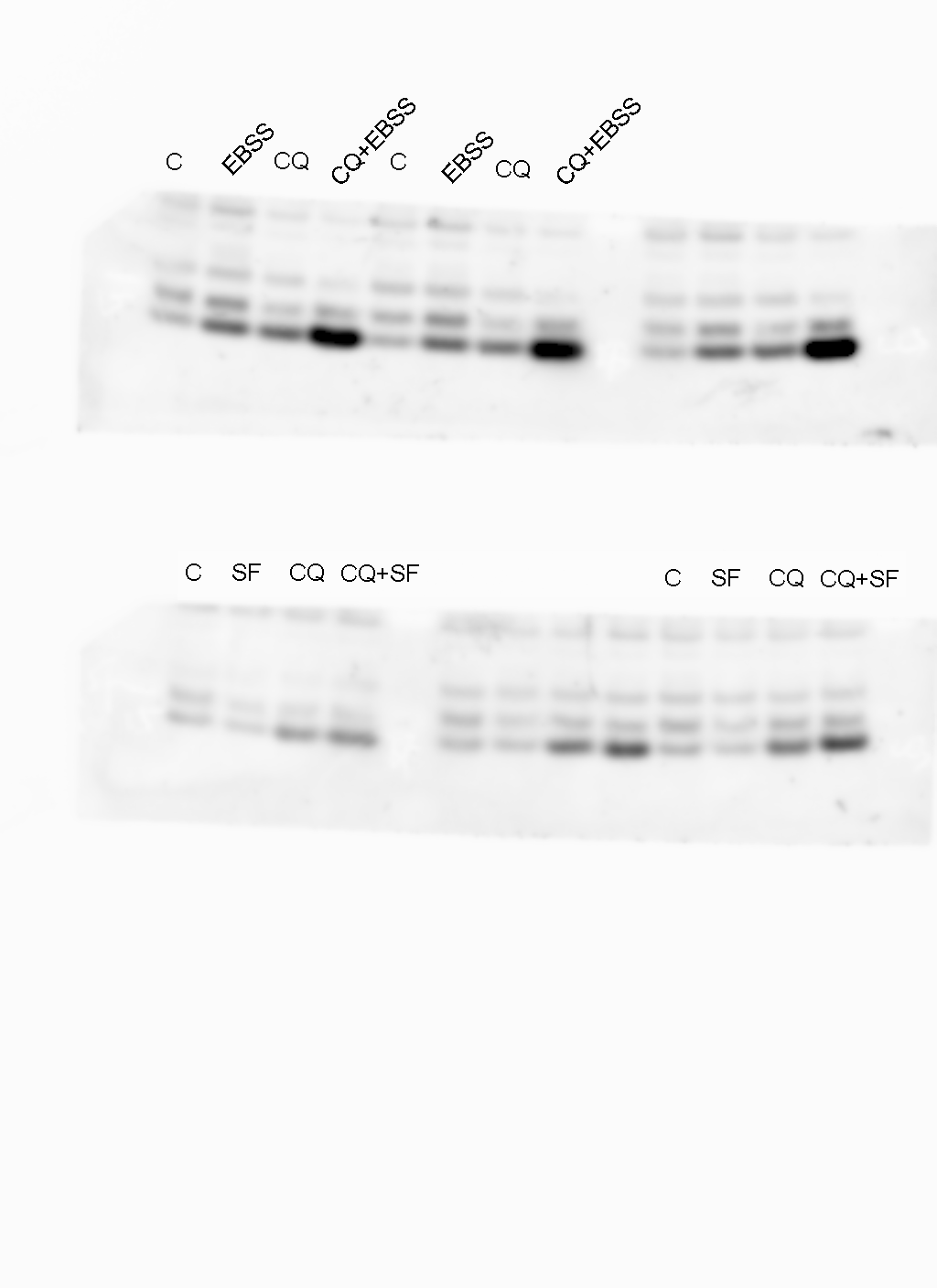


β-actin_(n2)


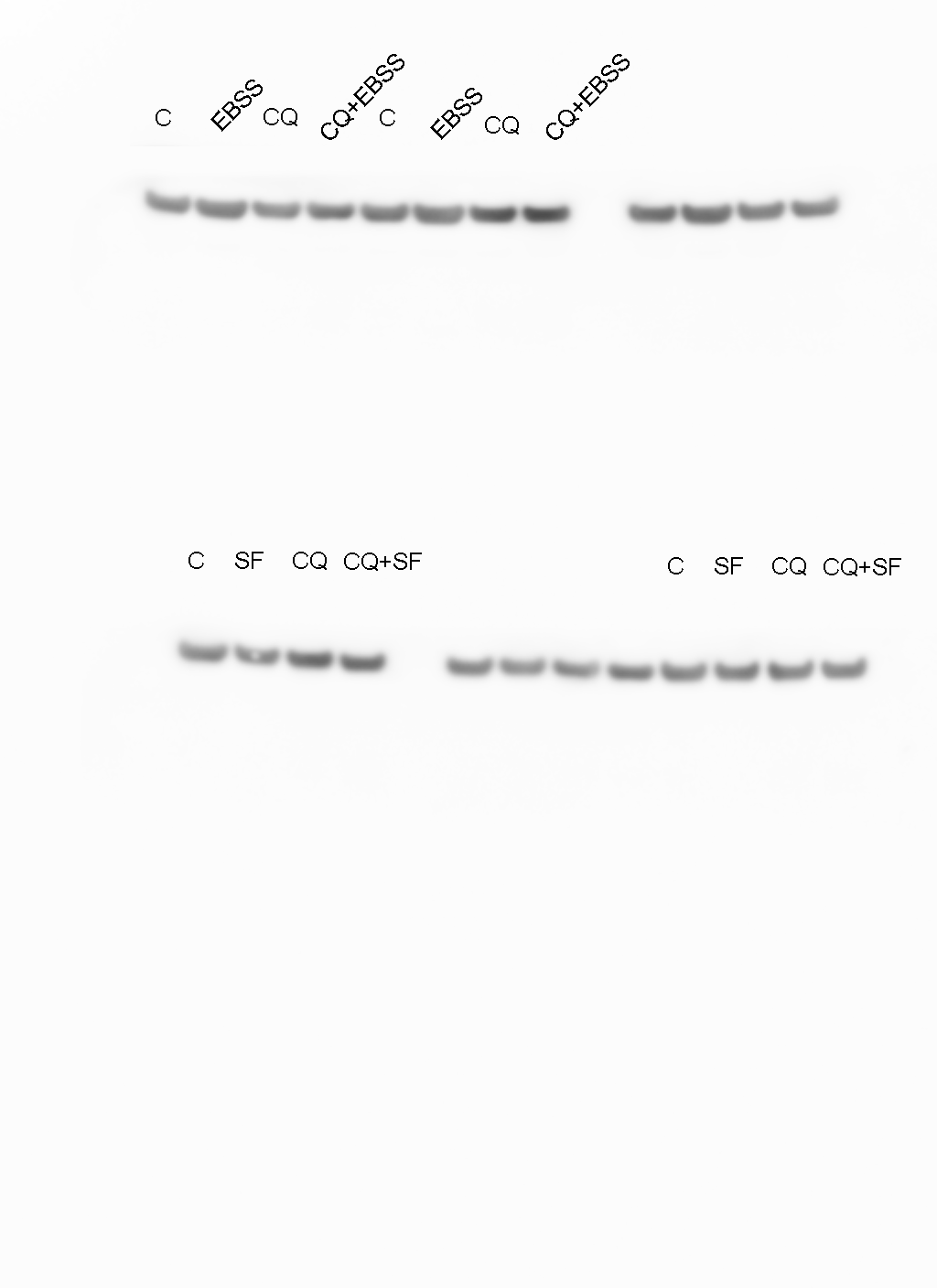


SQSTM1_(n3)


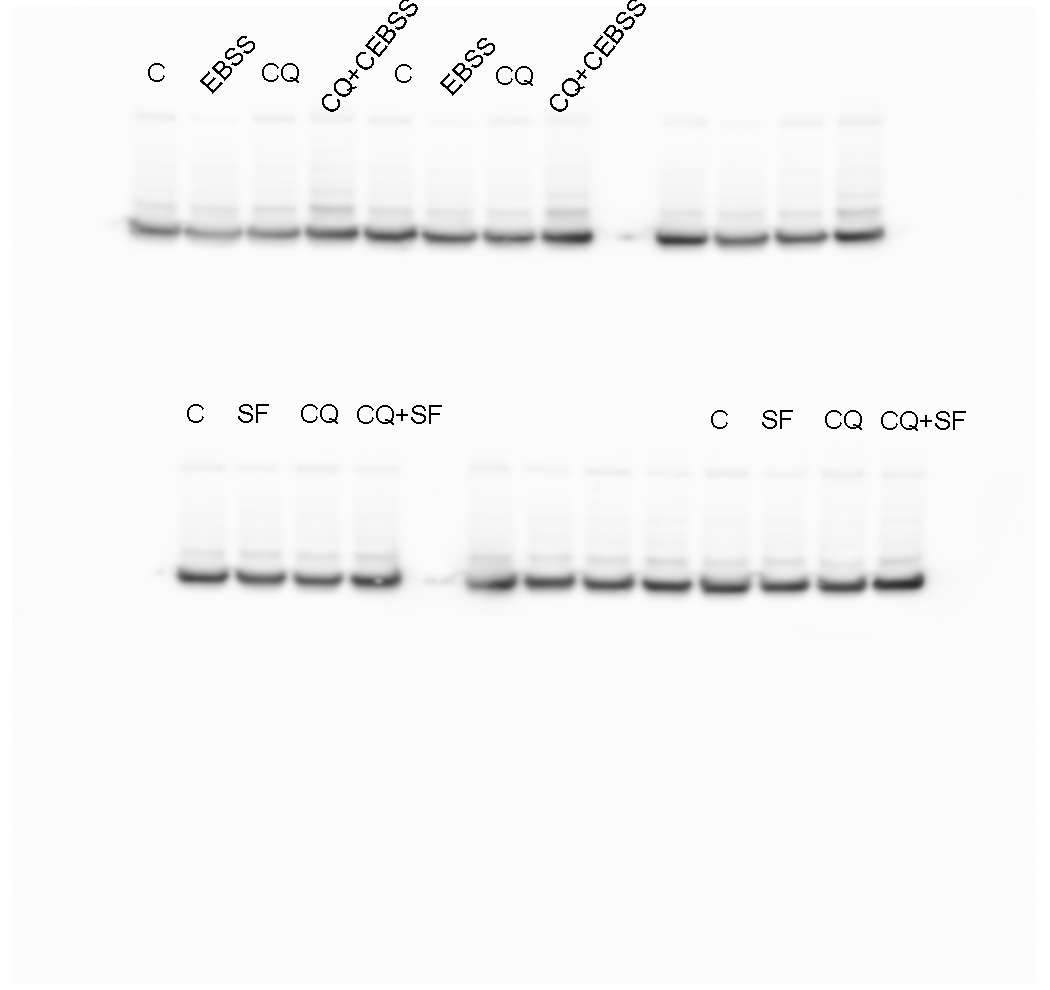


LC3_(n3)


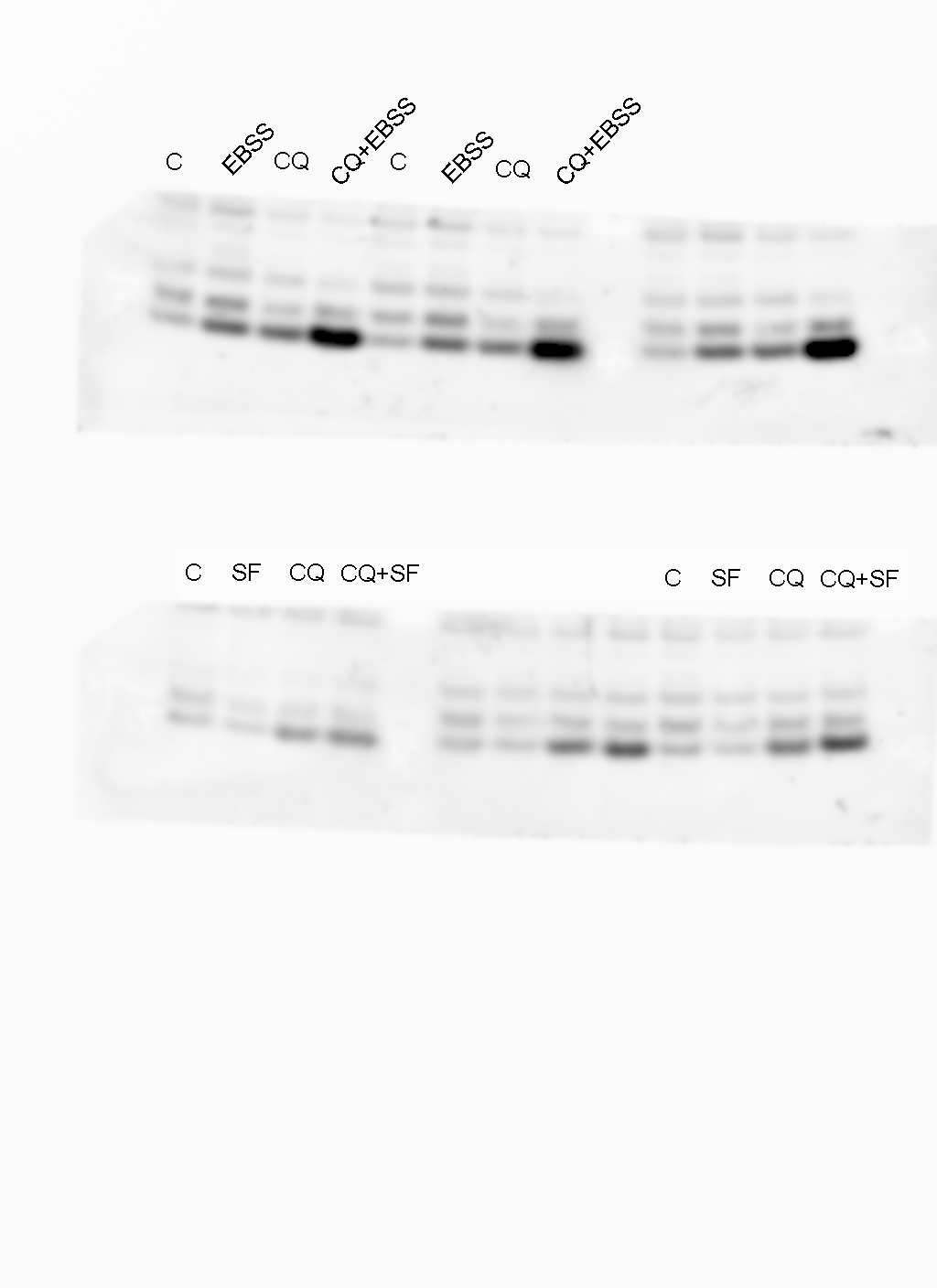


β-actin_(n3)


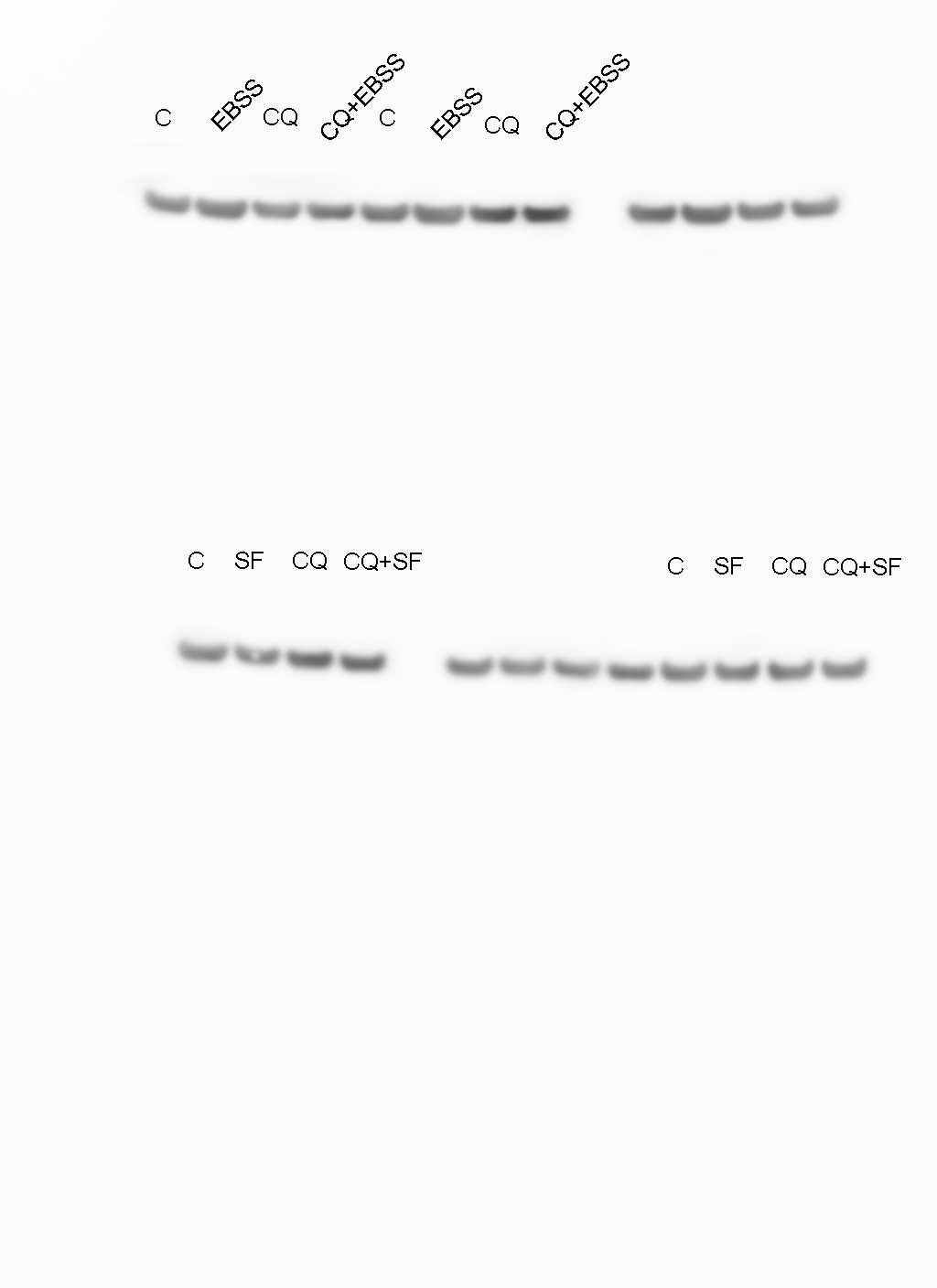


Figure 3F

Luciferase-Full length_(n1)


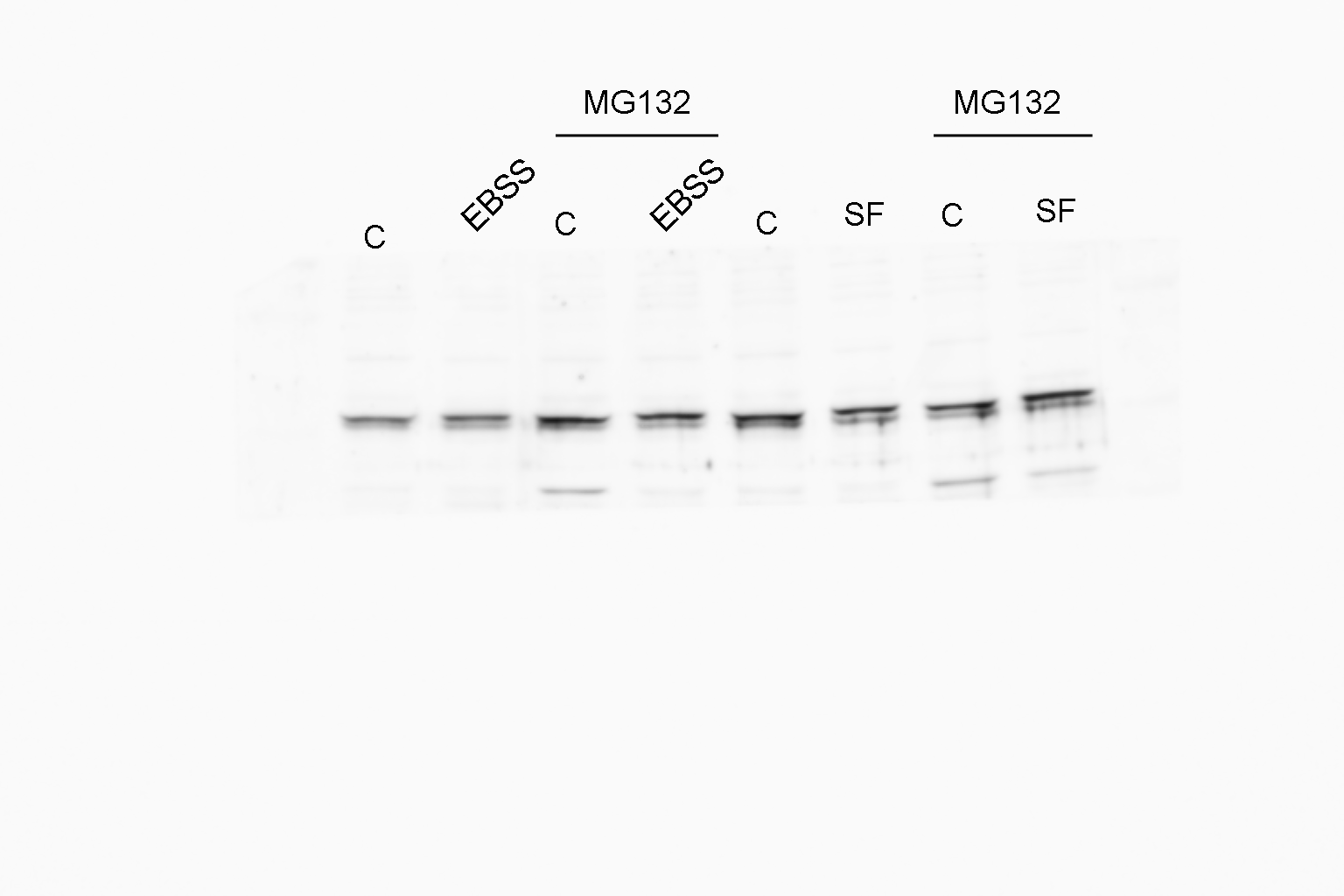


Luciferase-cleavage_(n1)


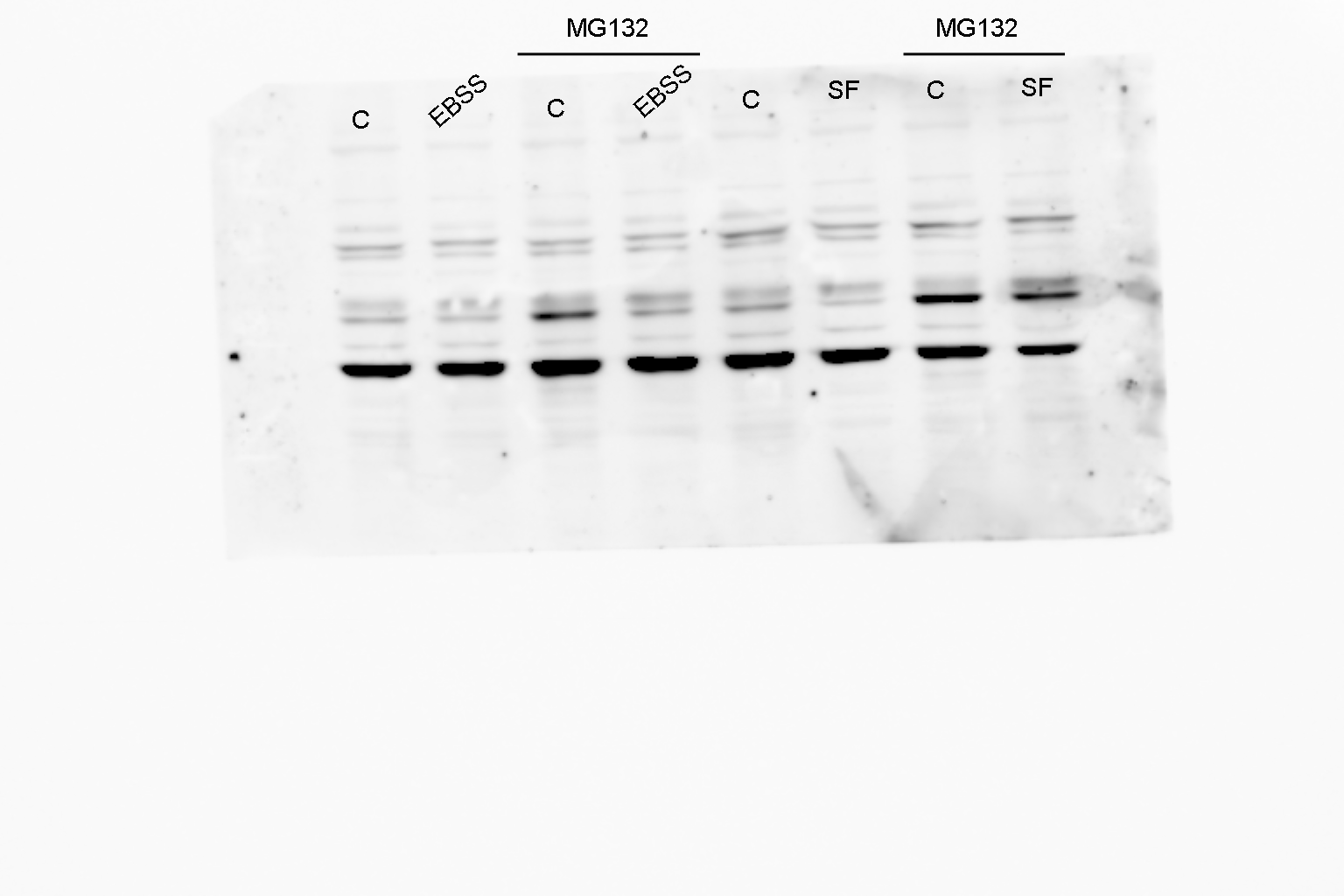


GAPDH_(n1)


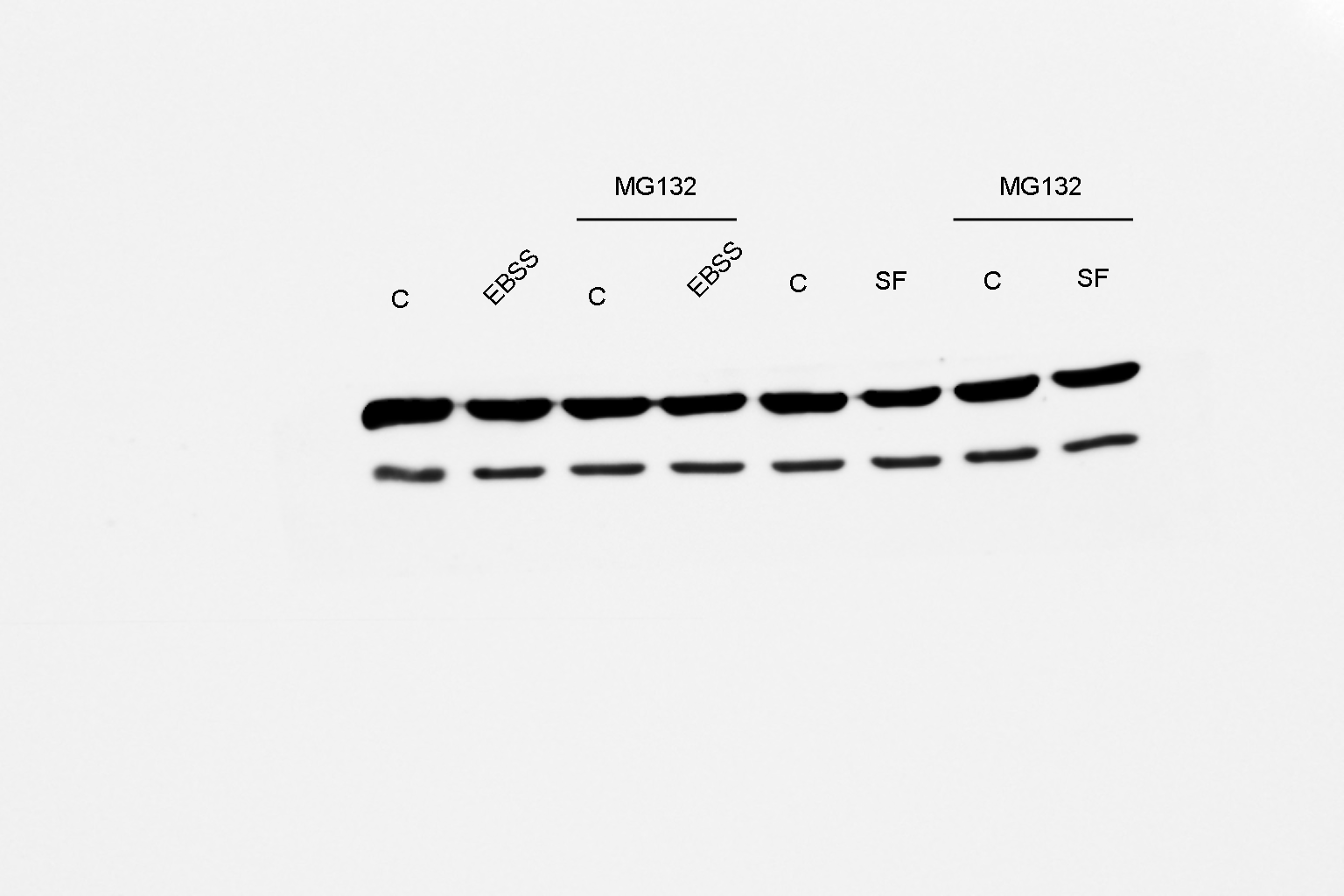


Luciferase-Full length_(n2)


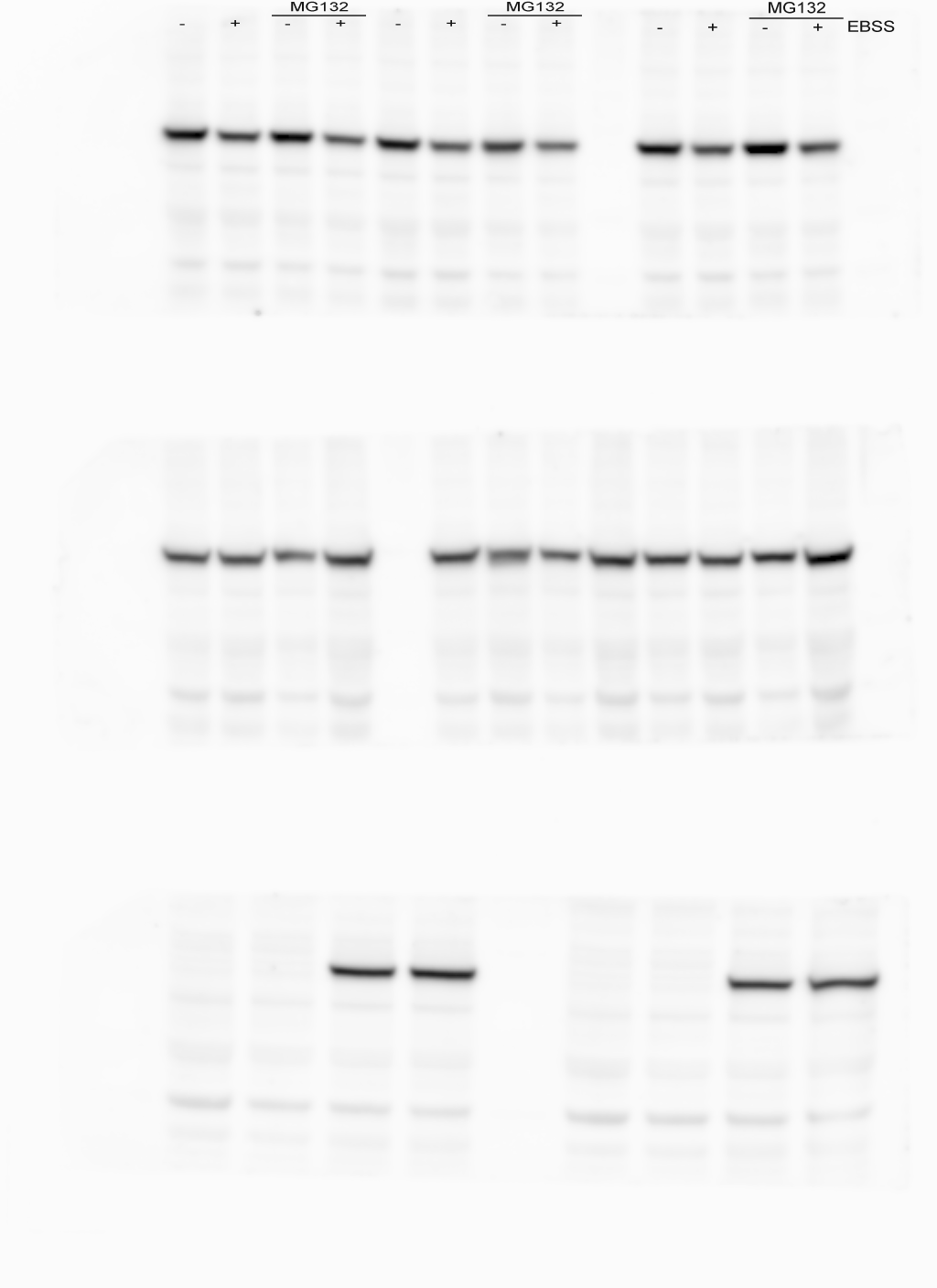


Luciferase-cleavage_(n2)


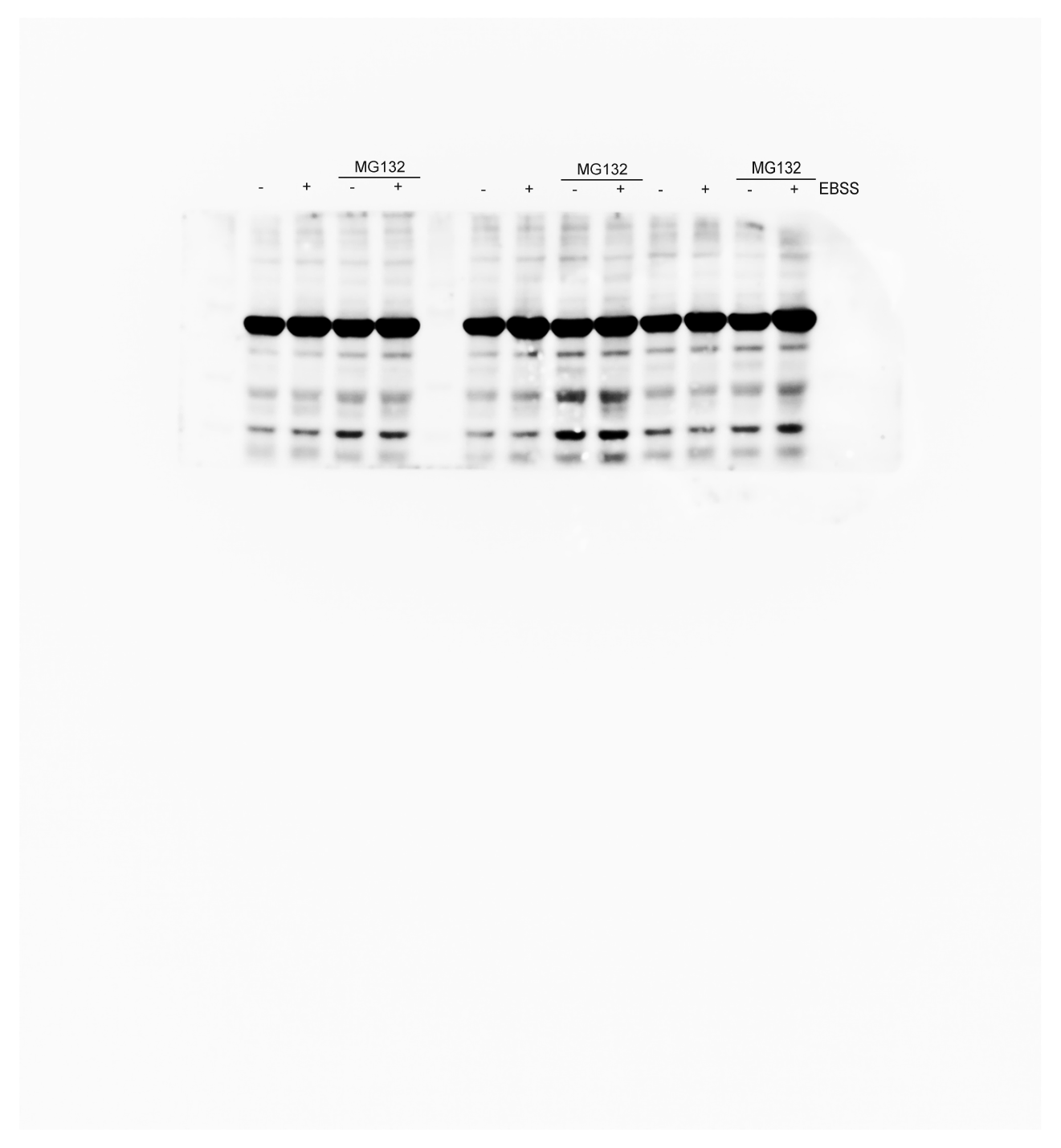


GAPDH_(n2)


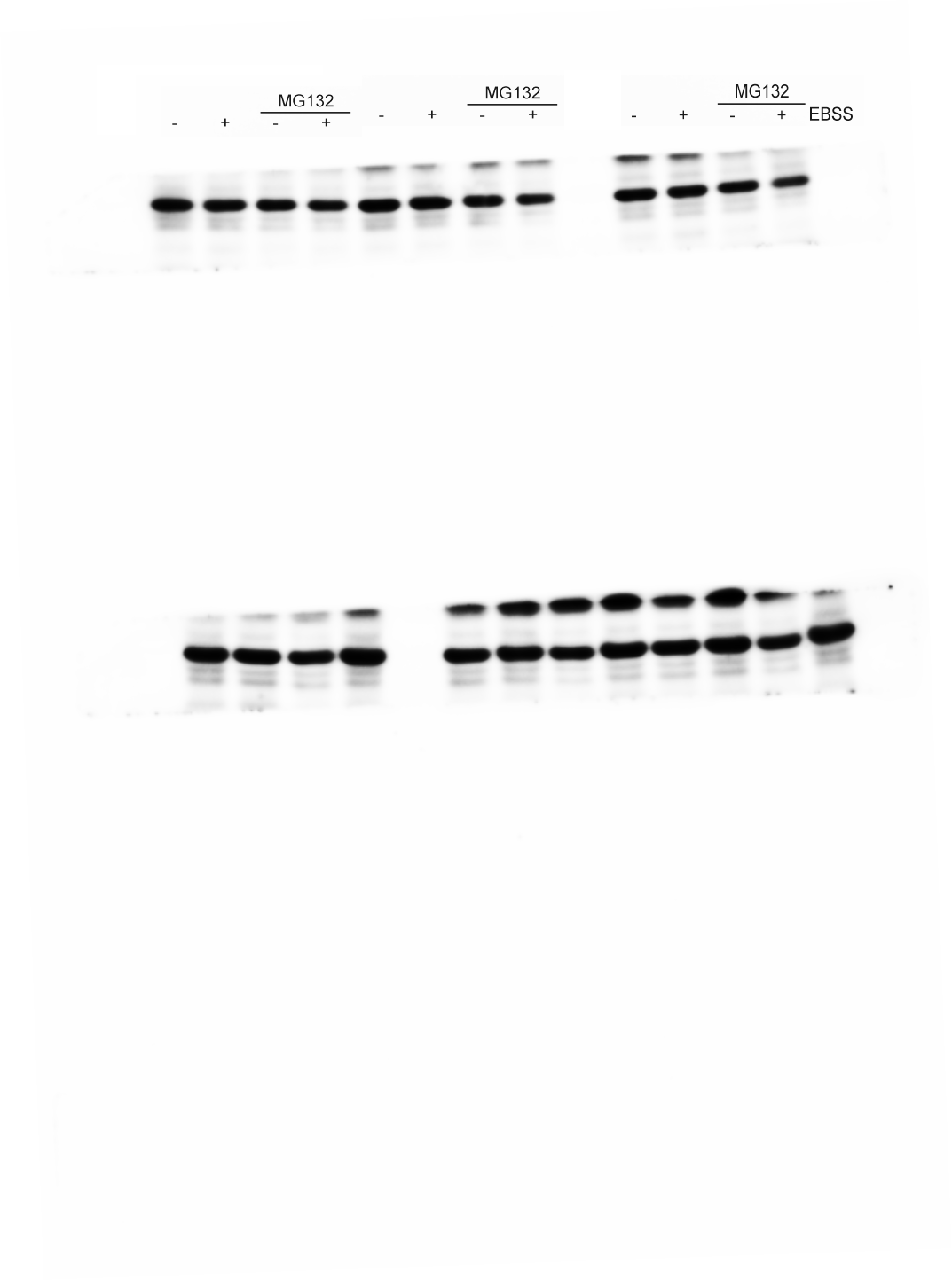


Luciferase-Full length_(n3)


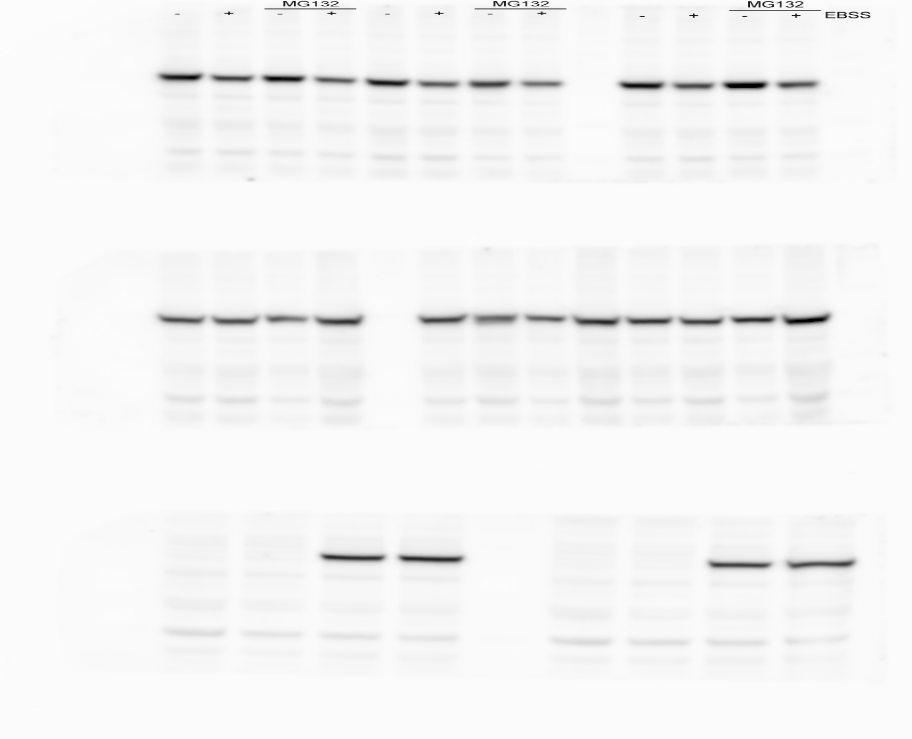


Luciferase-cleavage_(n3)





GAPDH_(n3)


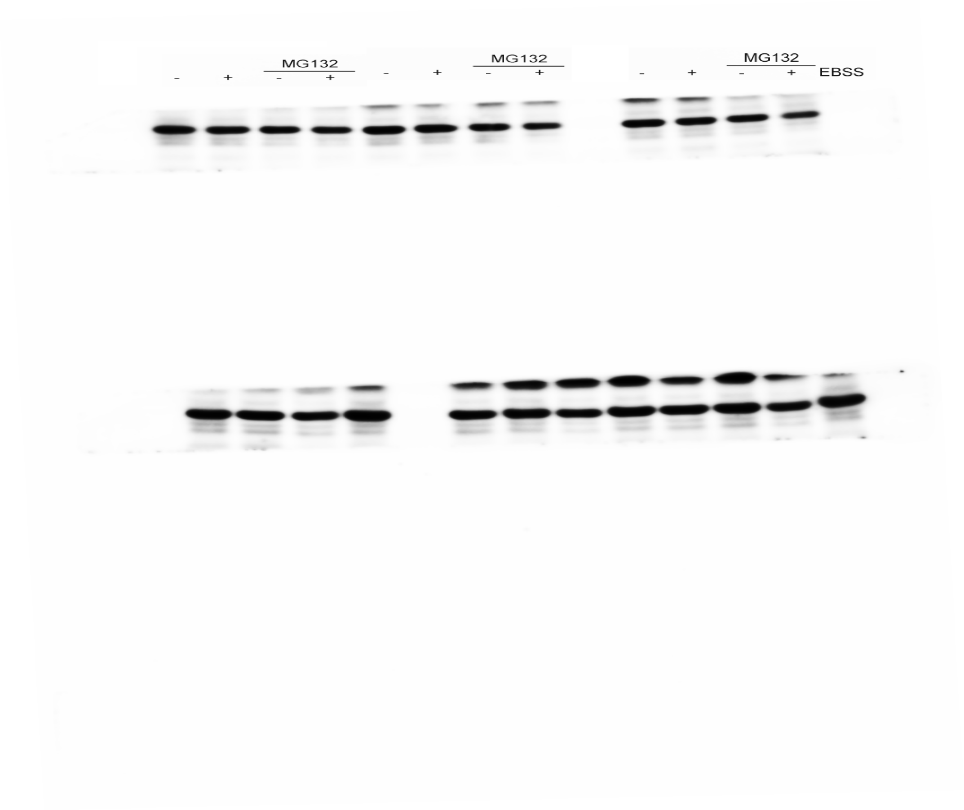


Figure 3G

Luciferase-Full length_(n1)


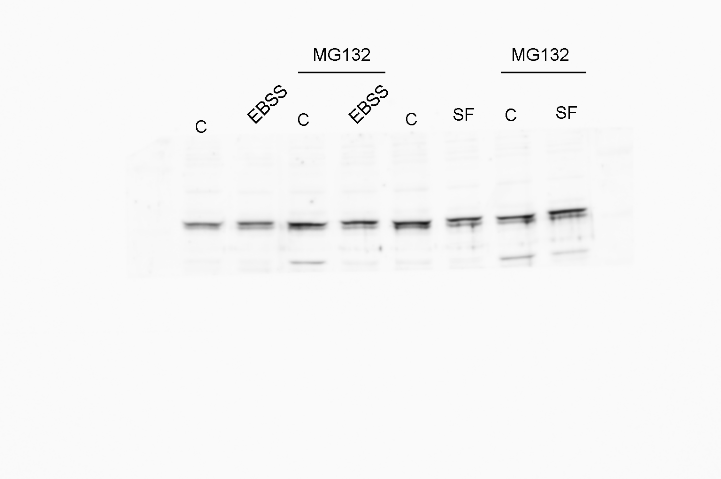


Luciferase-Cleavage_(n1)


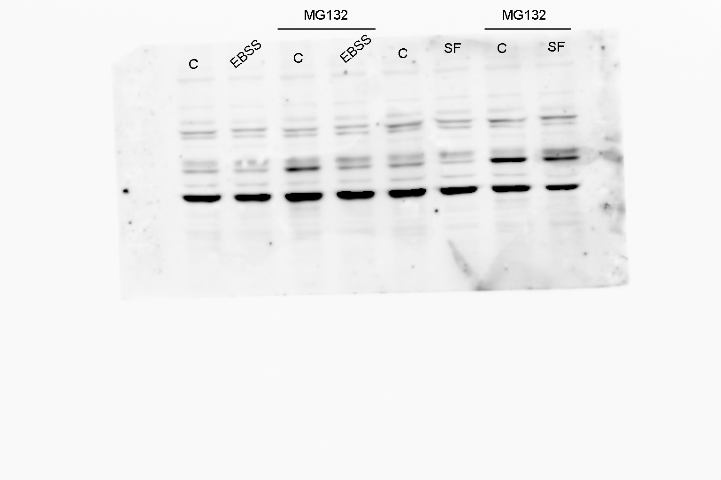


GAPDH_(n1)


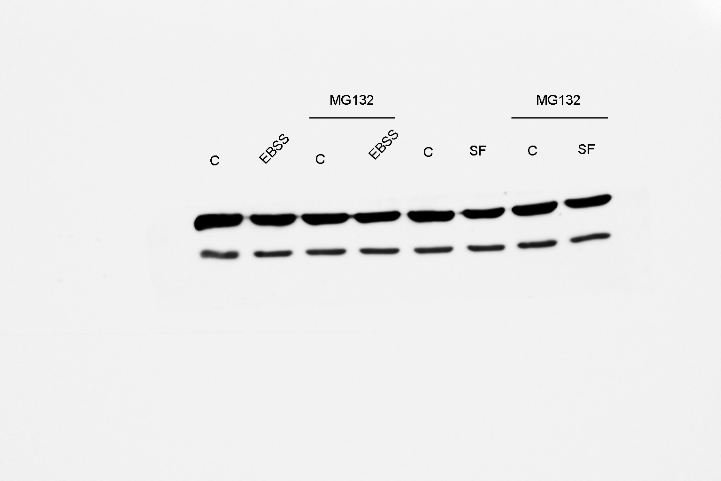


Luciferase-Full length_(n2)


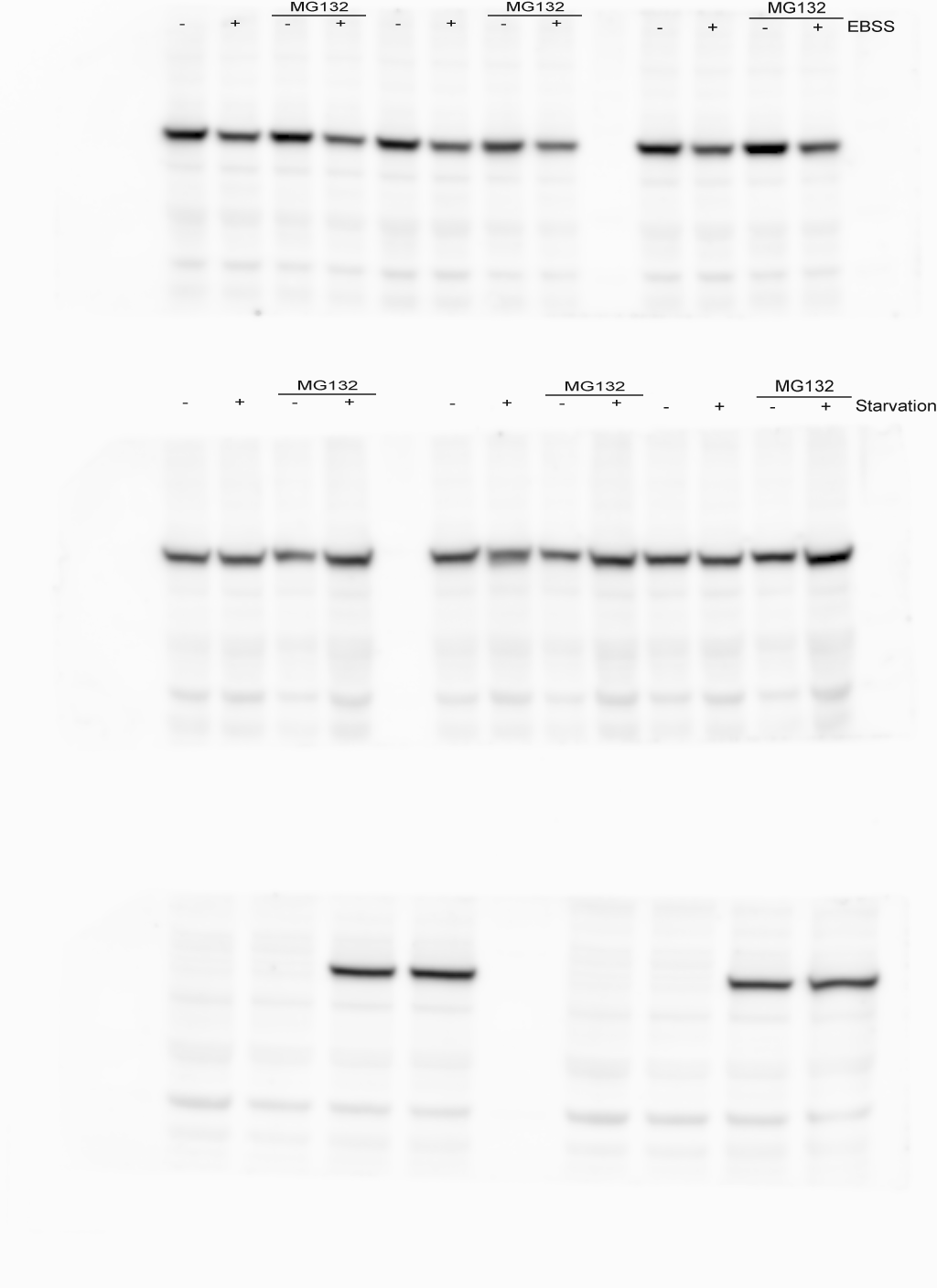


Luciferase-Cleavage_(n2)


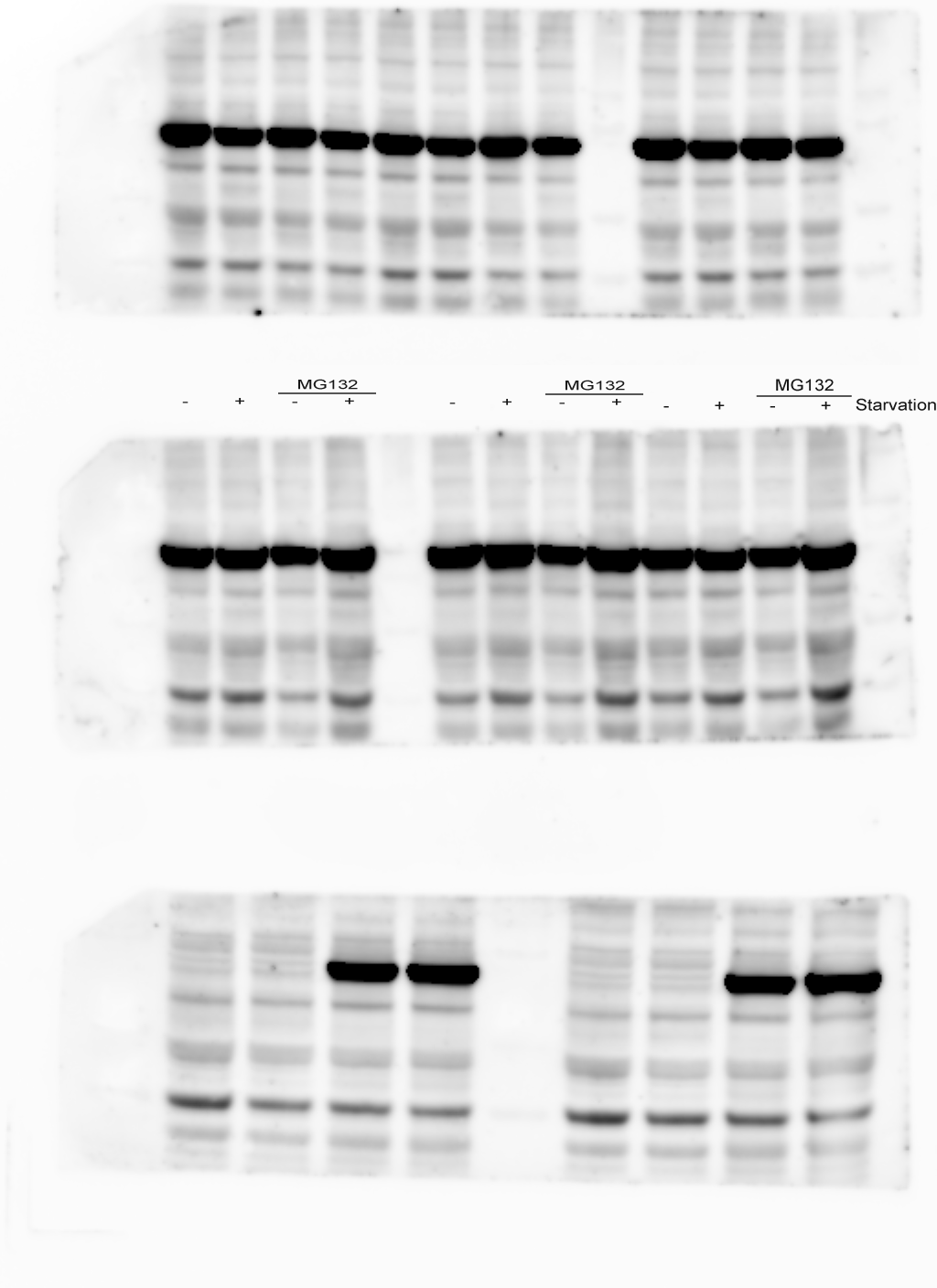


GAPDH_(n2)


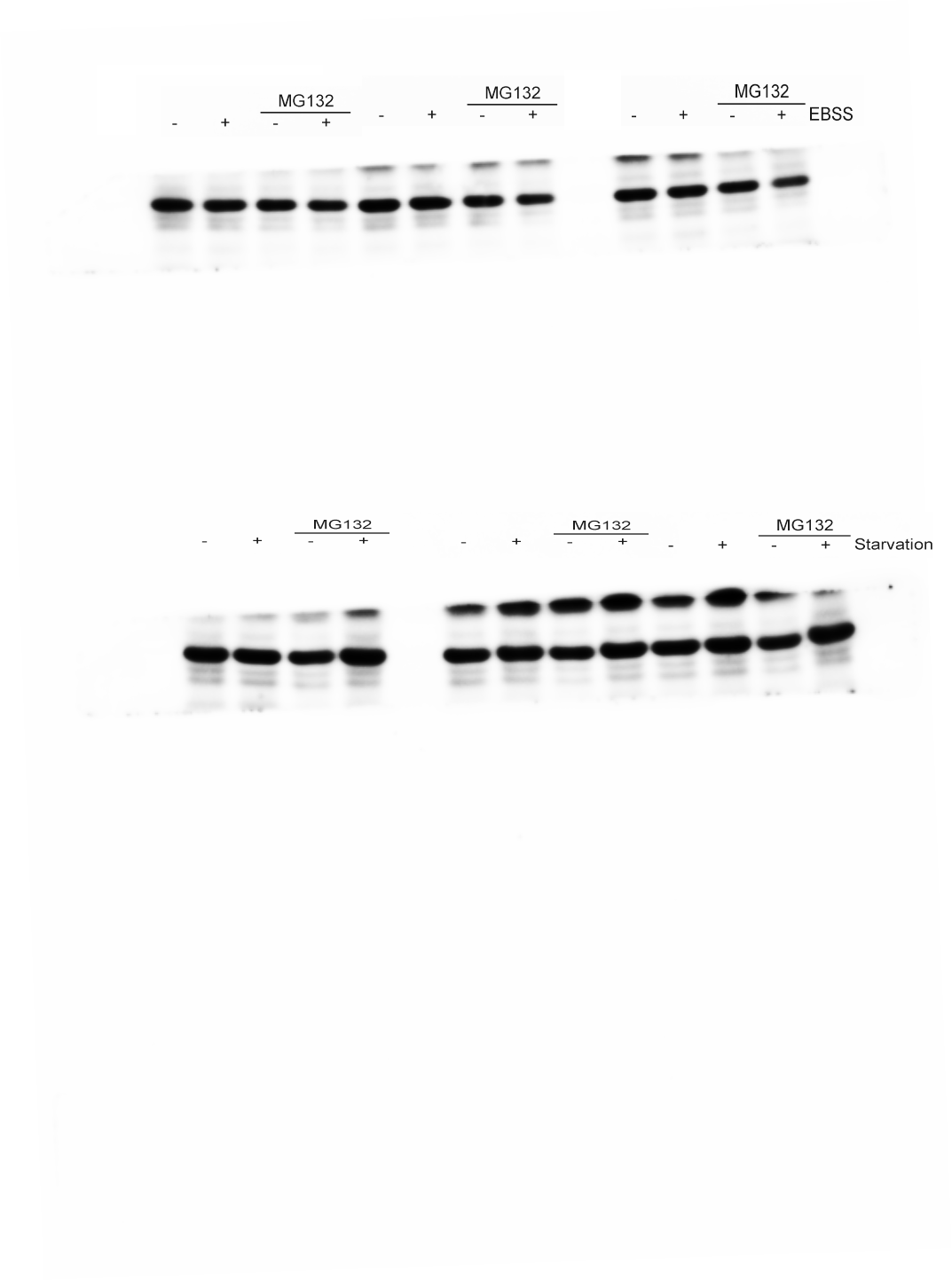


Luciferase-Full length_(n3)


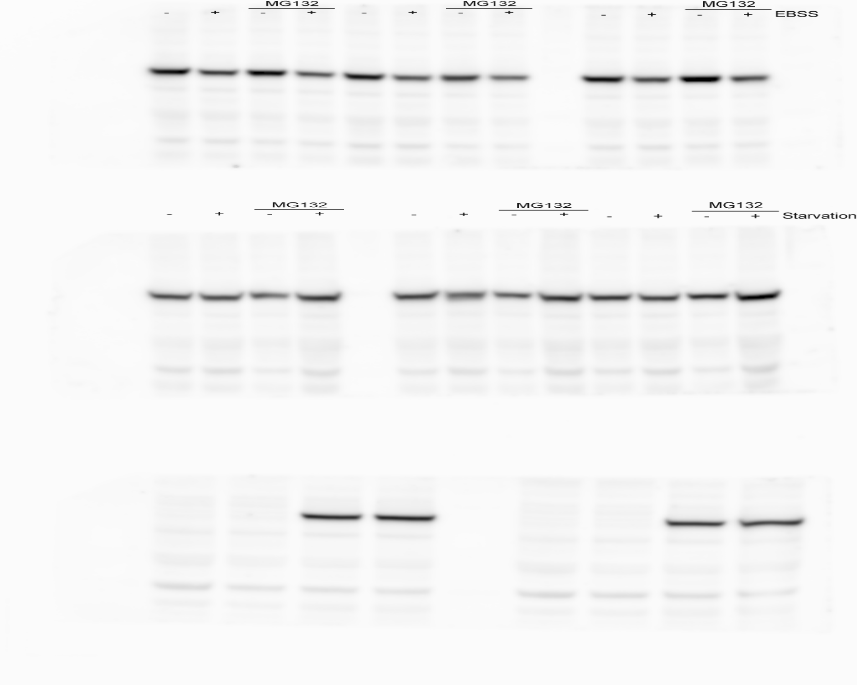


Luciferase-Cleavage_(n3)


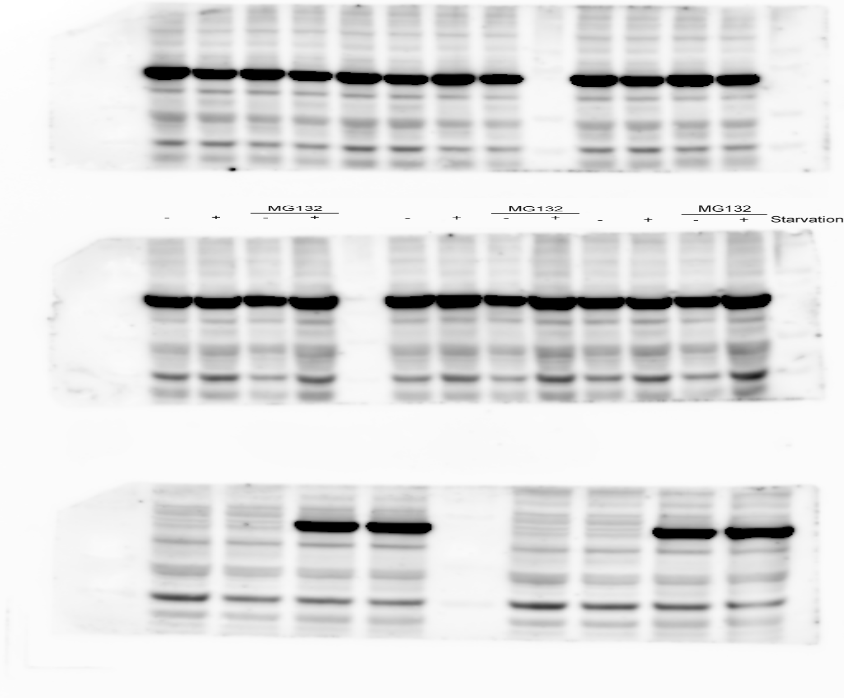


GAPDH_(n3)


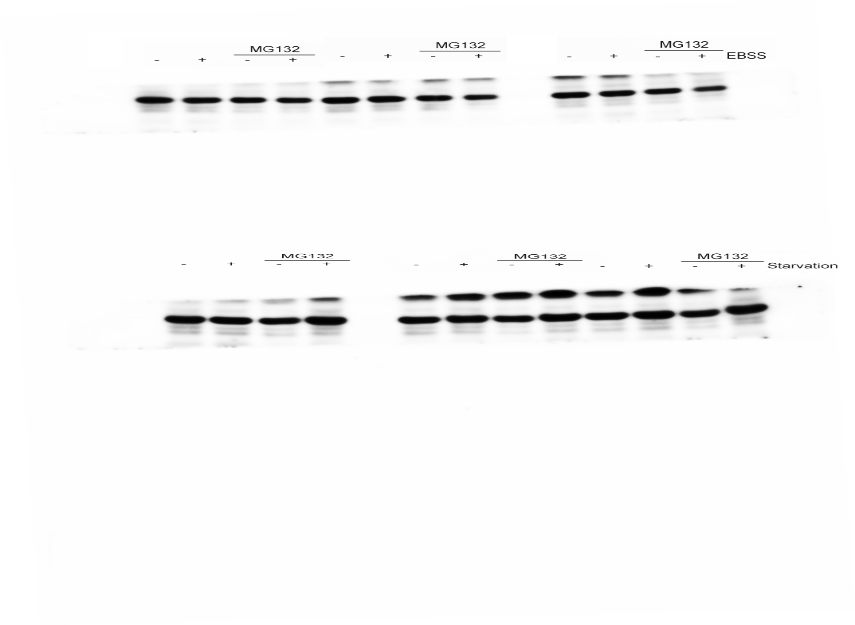


Figure 3H

Luciferase-Full length_(n1)


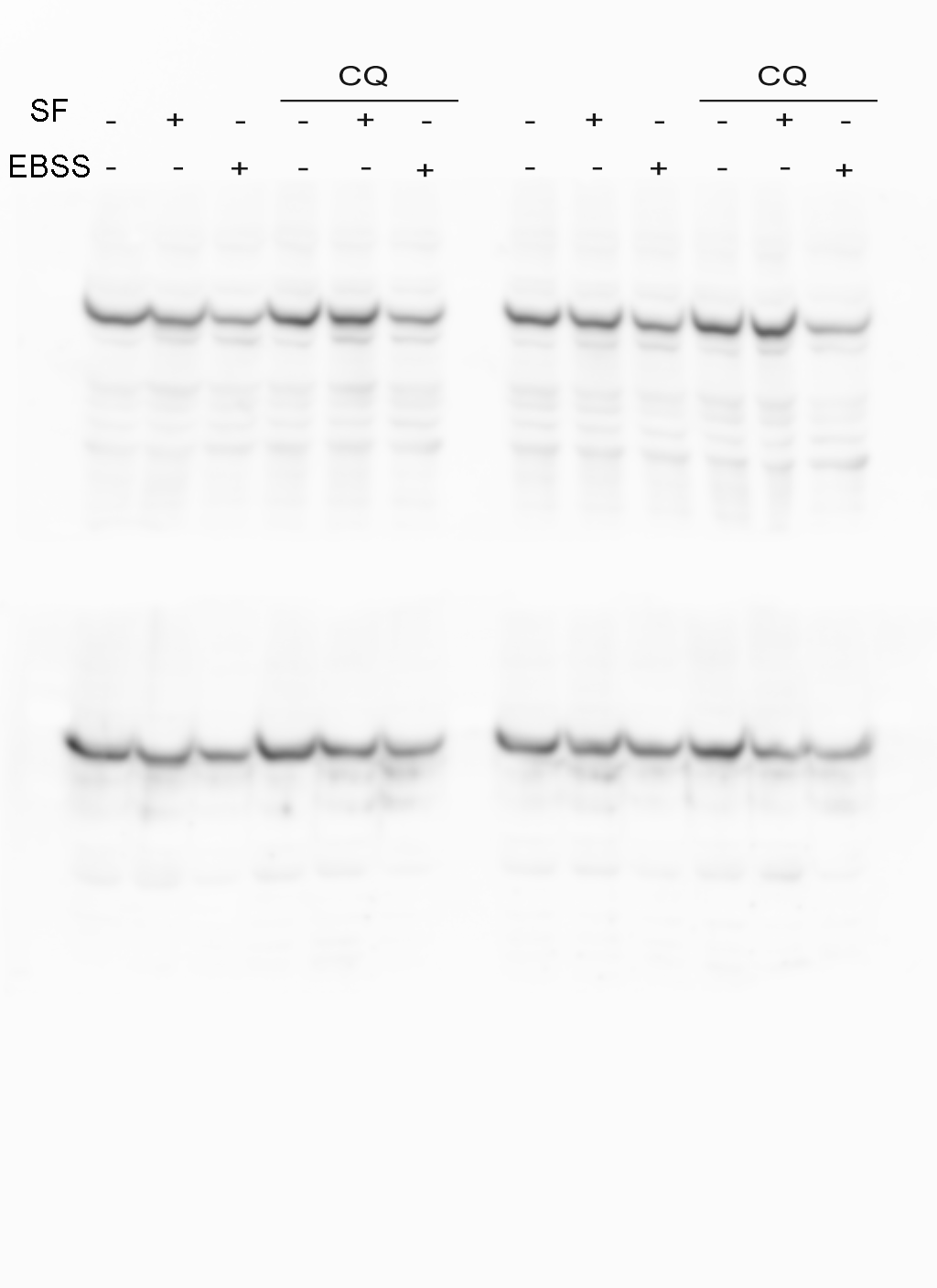


Luciferase-Cleavage_(n1)


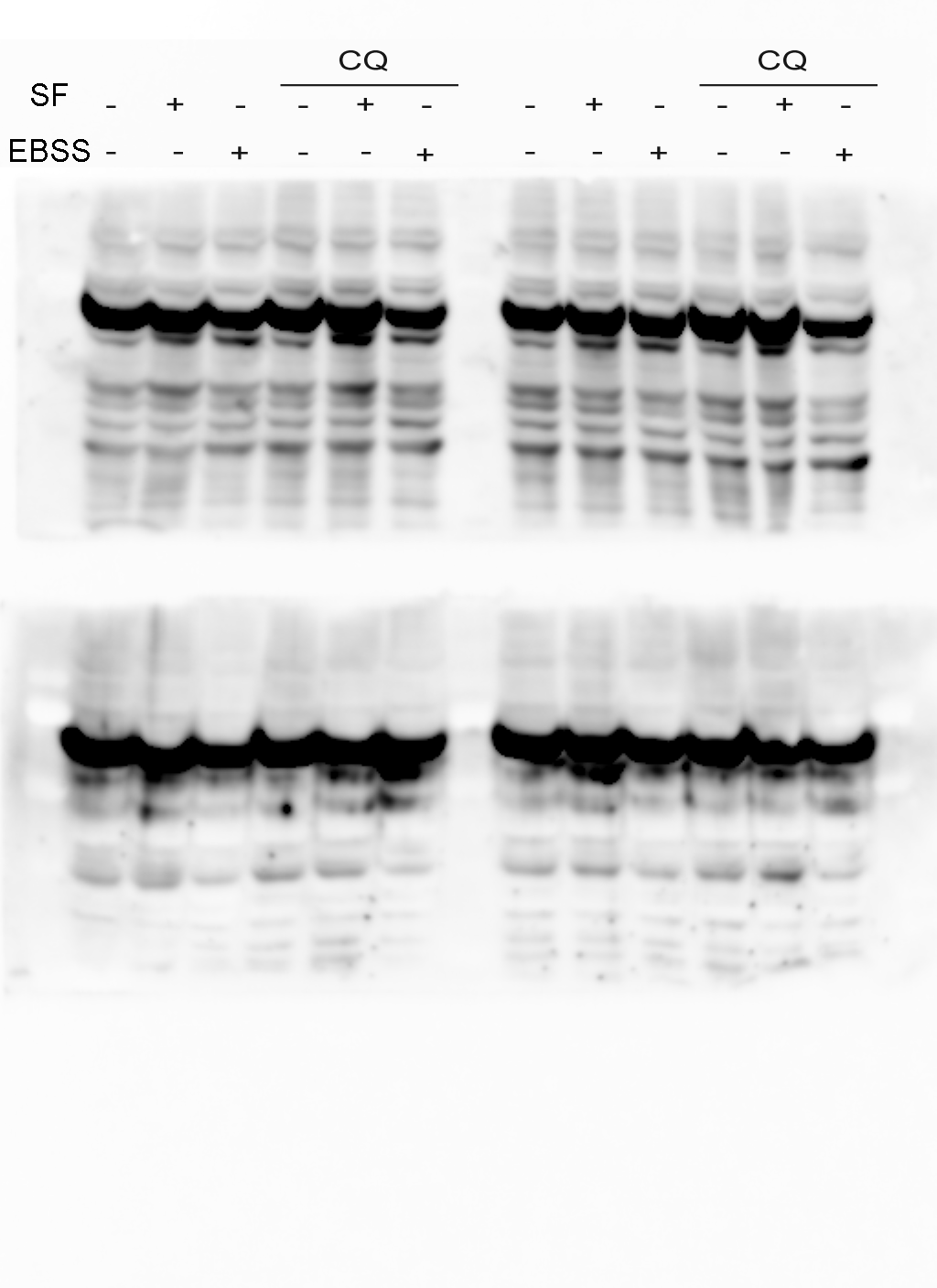


LC3_(n1)


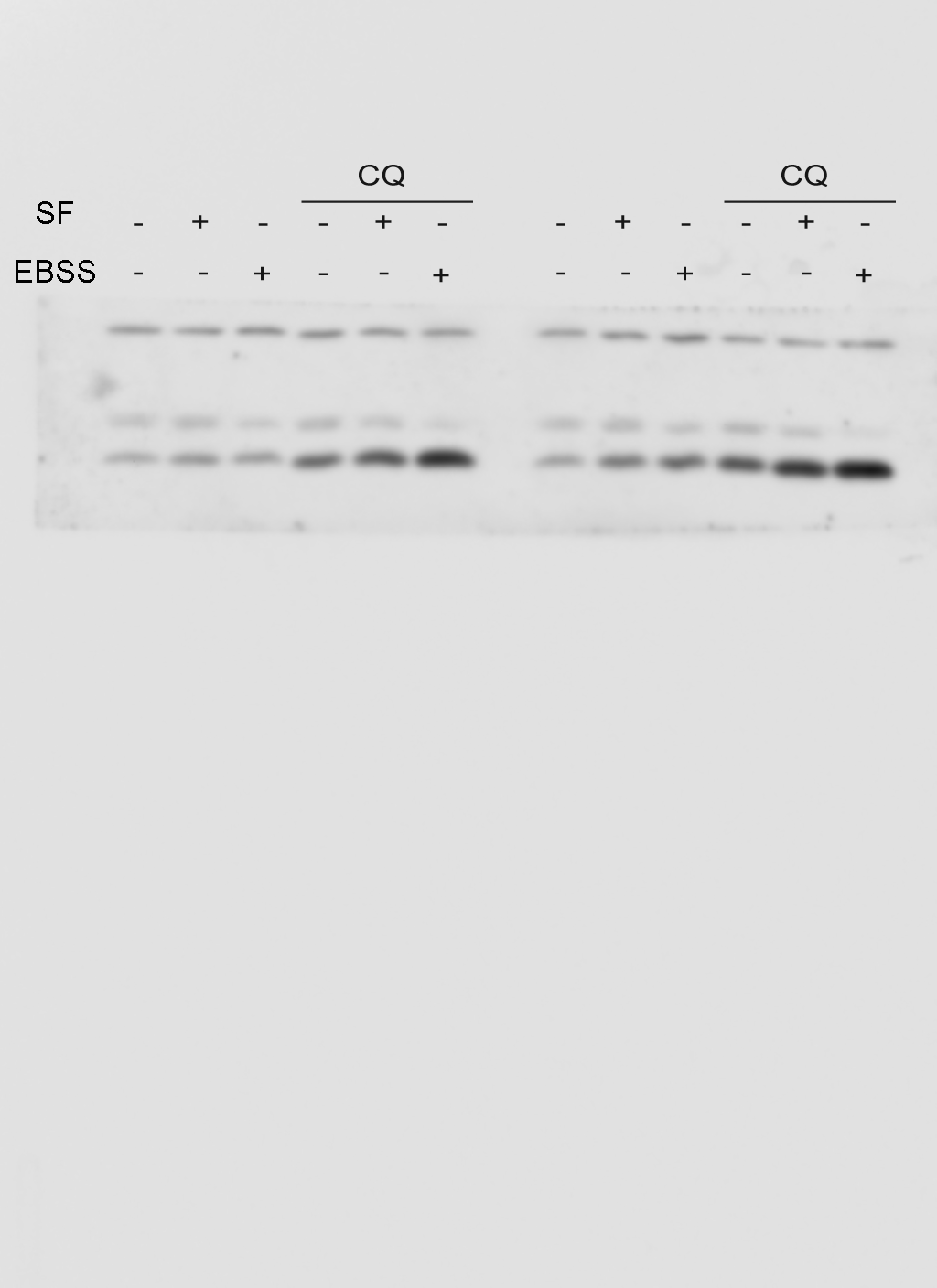


GAPDH_(n1)


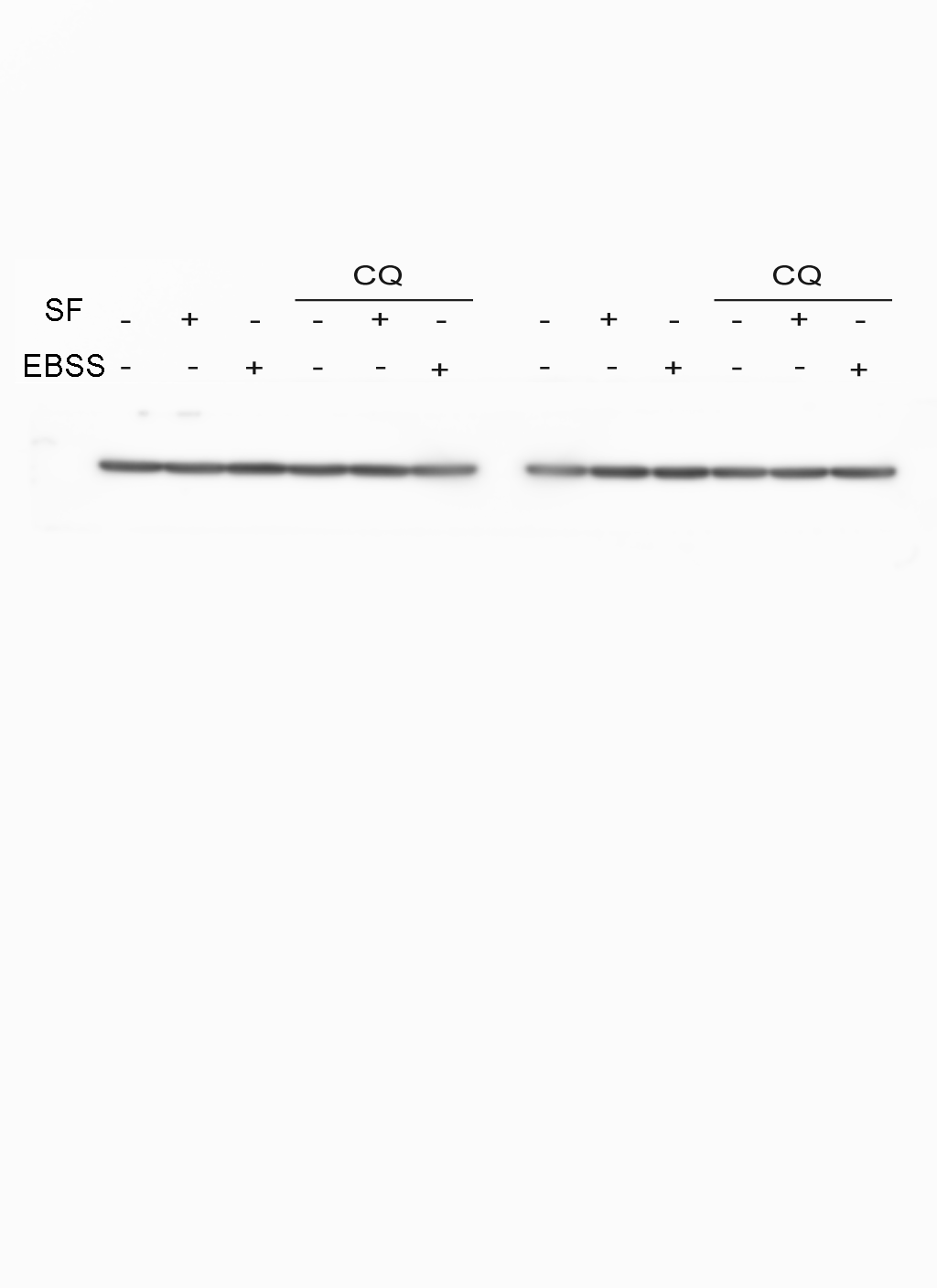


Luciferase-Full length_(n2)


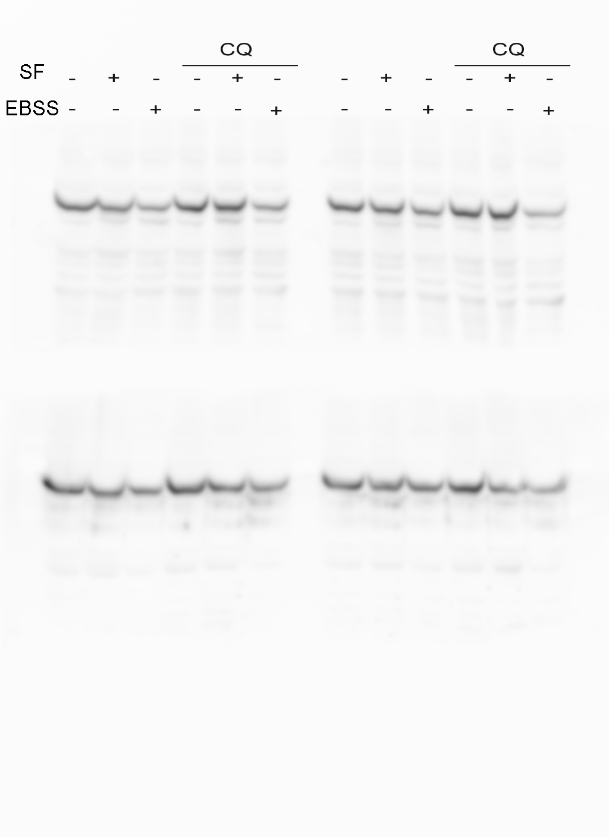


Luciferase-Cleavage_(n2)


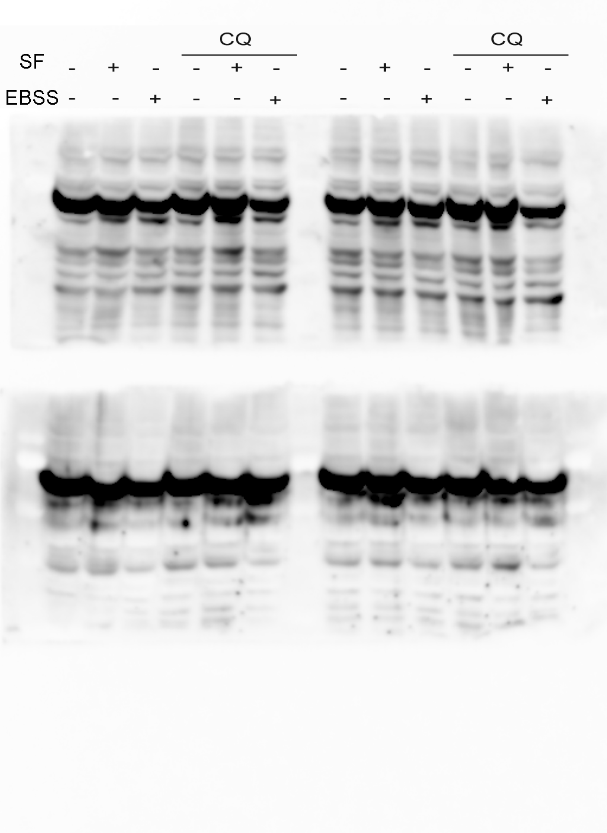


LC3_(n2)


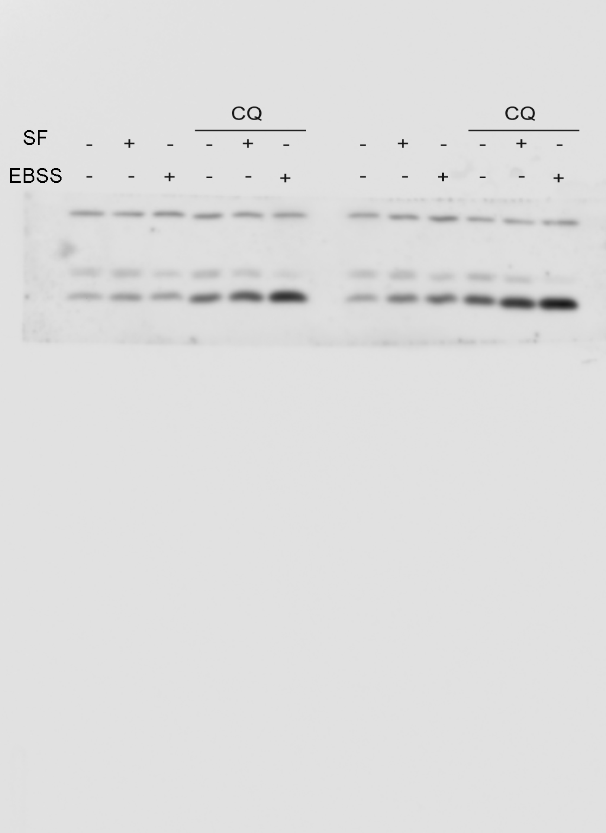


GAPDH_(n2)


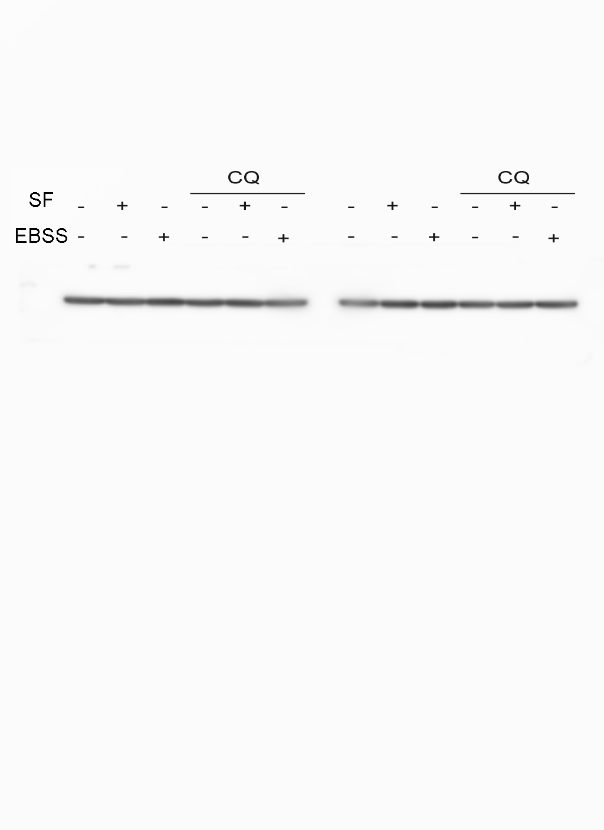


Luciferase-Full length_(n3)


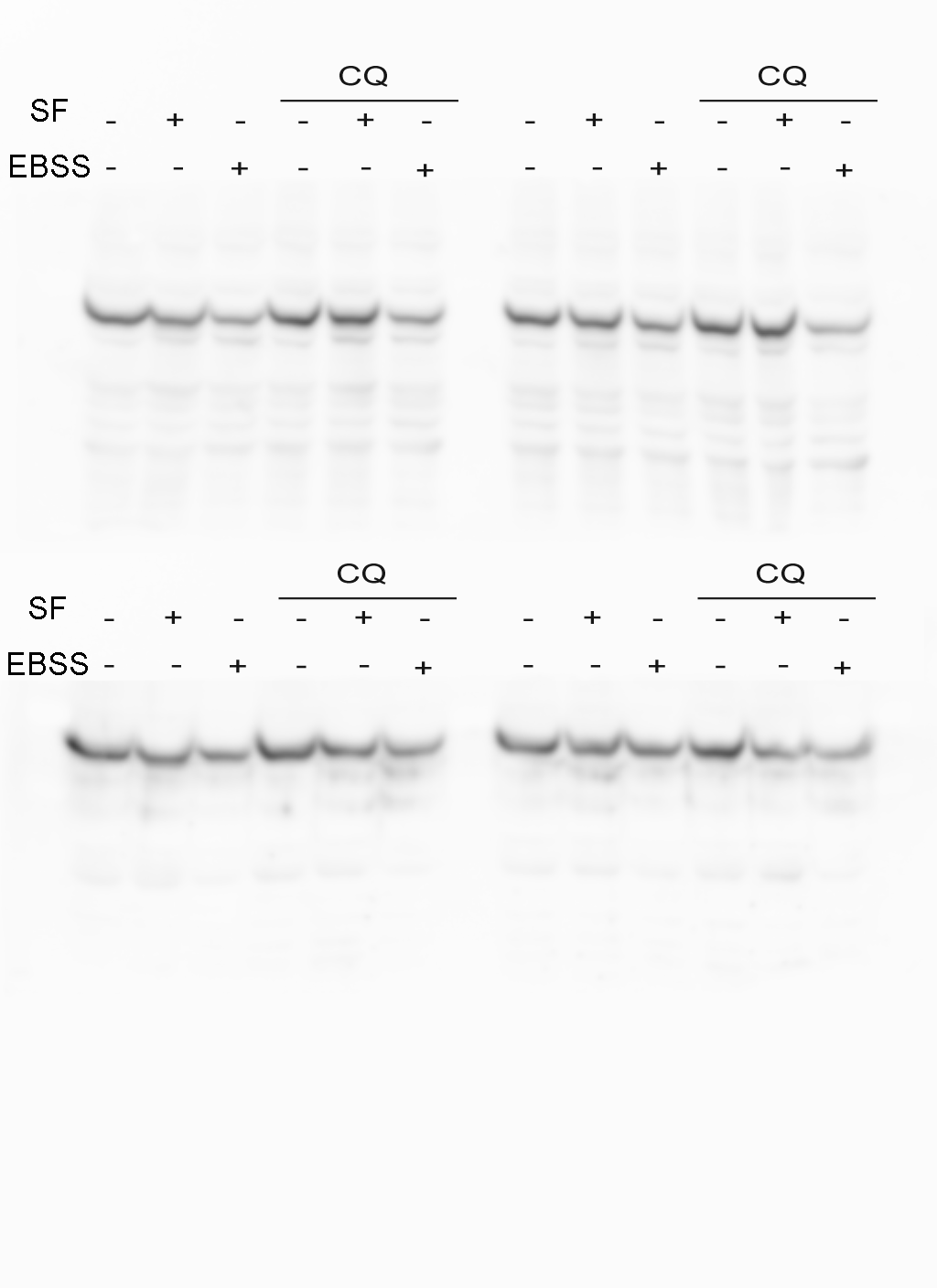


Luciferase-Cleavage_(n3)


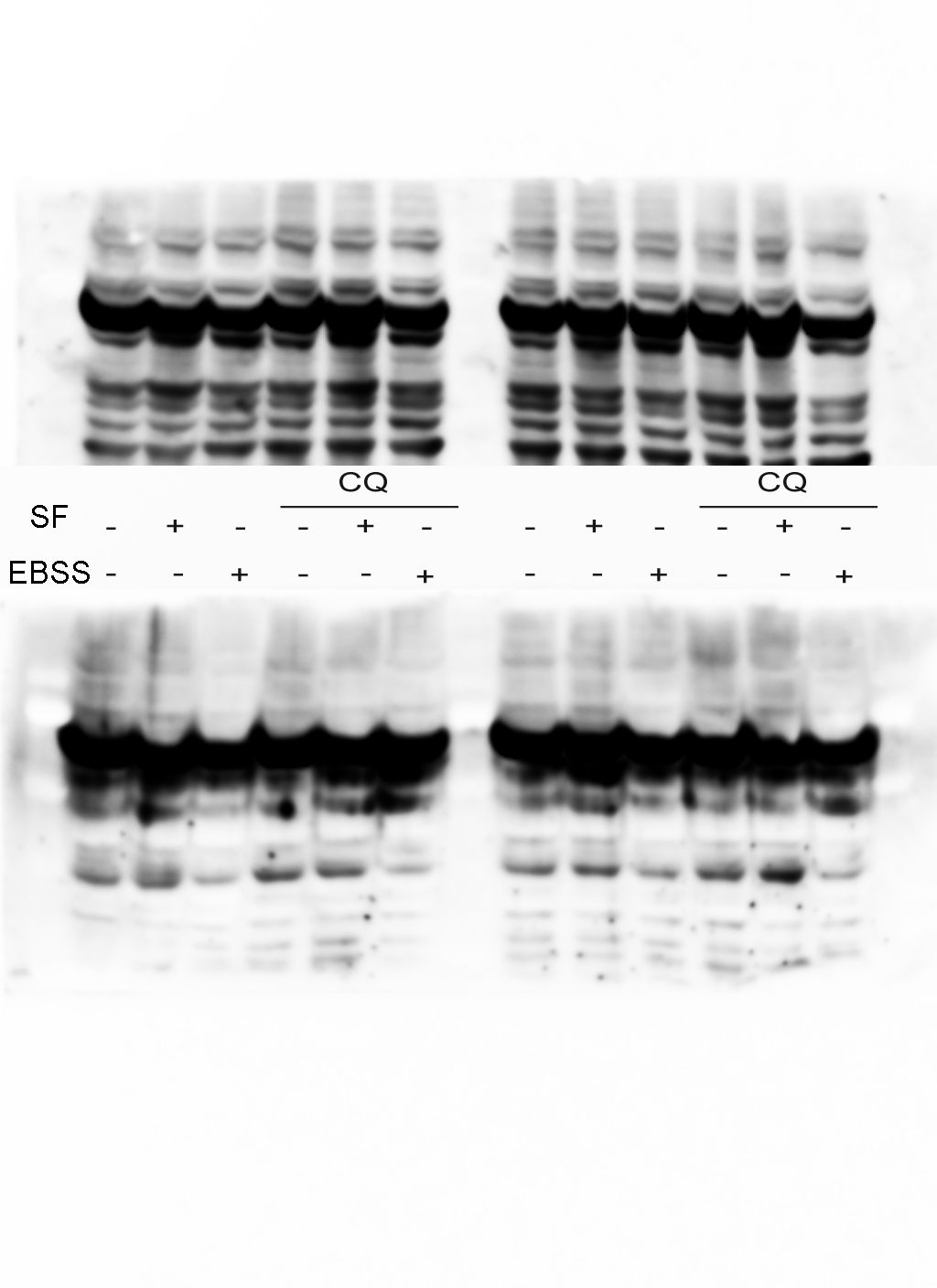


LC3_(n3)


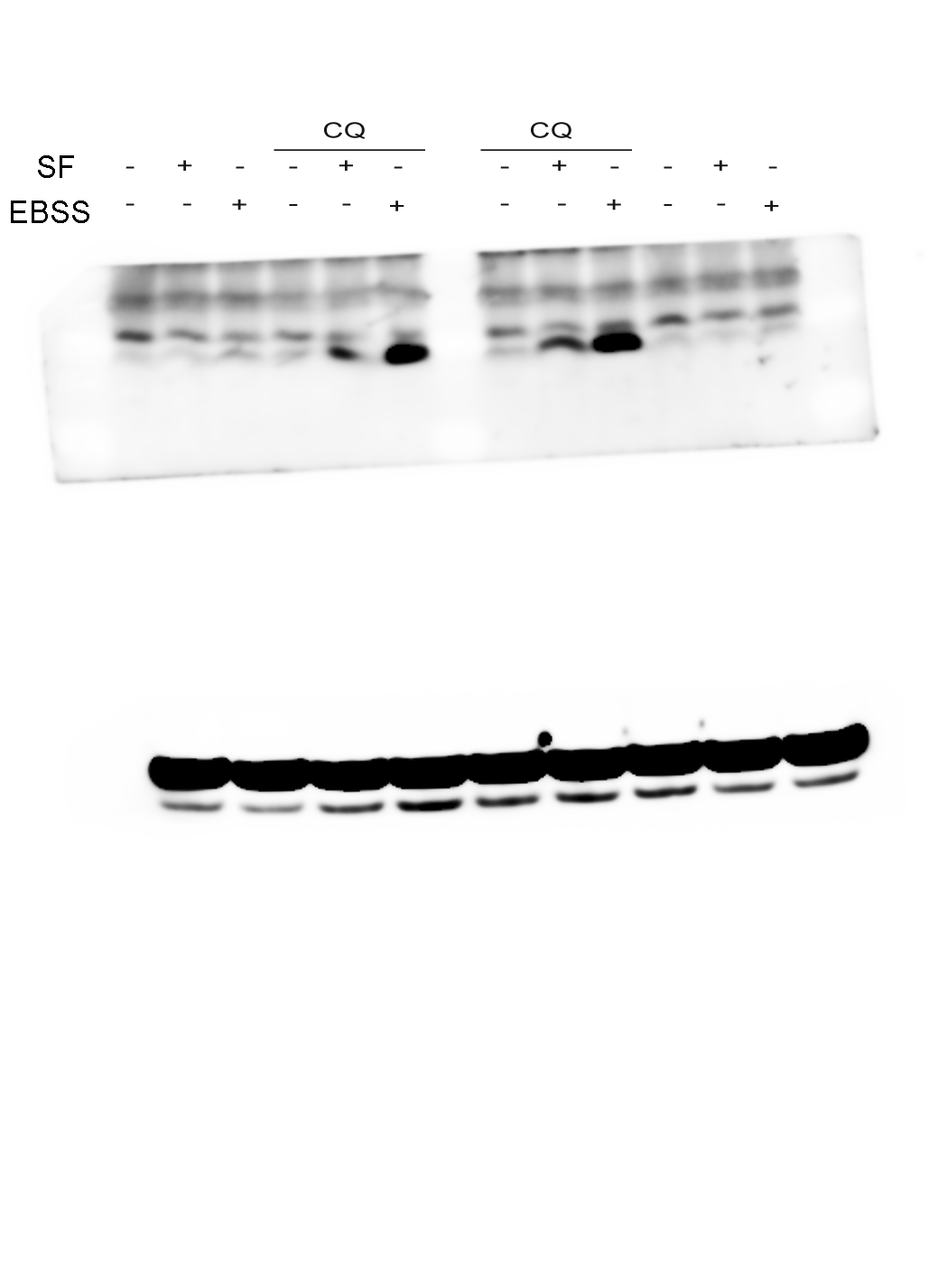


GAPDH_(n3)


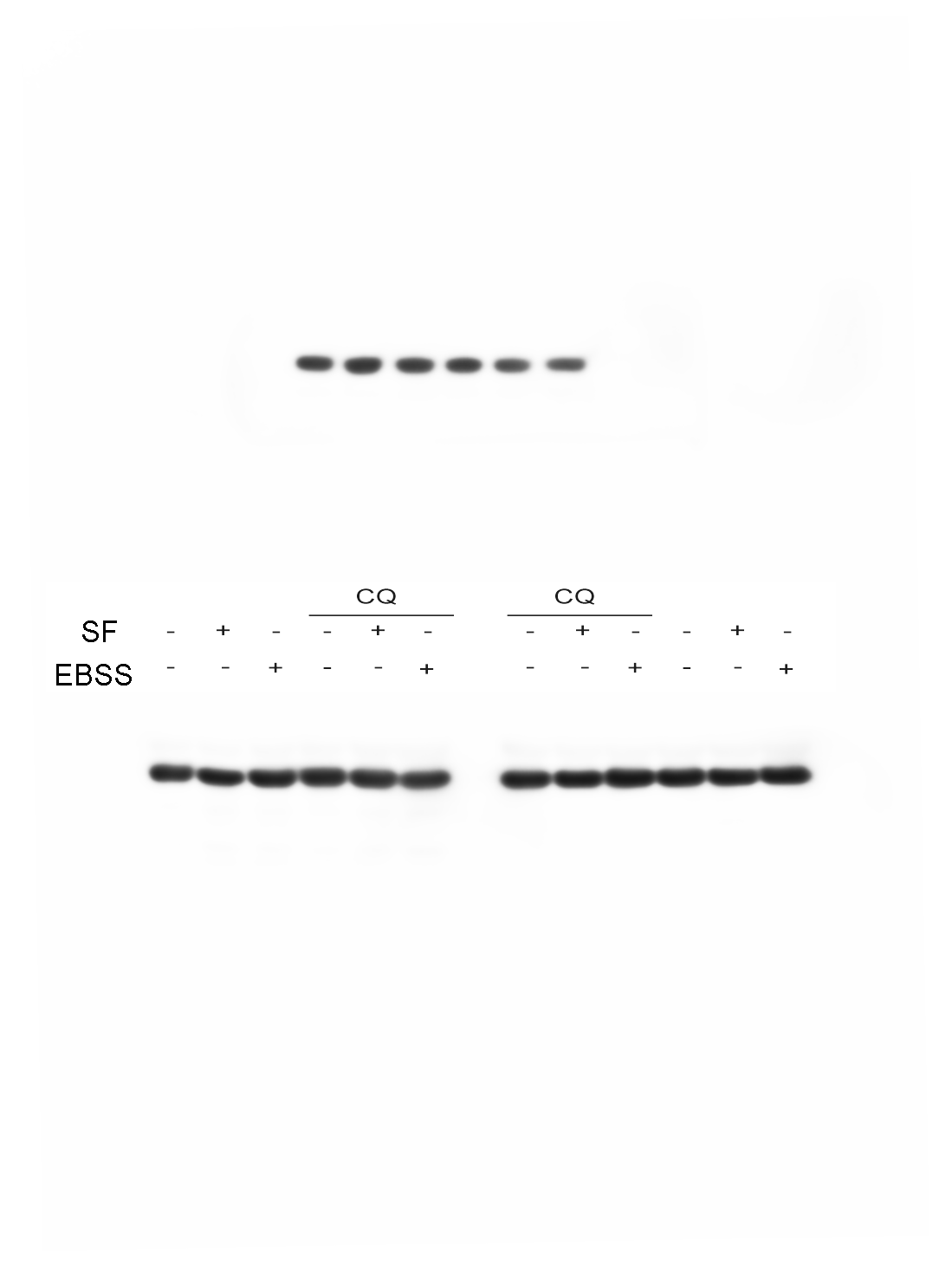


Figure 4D

ATG4B


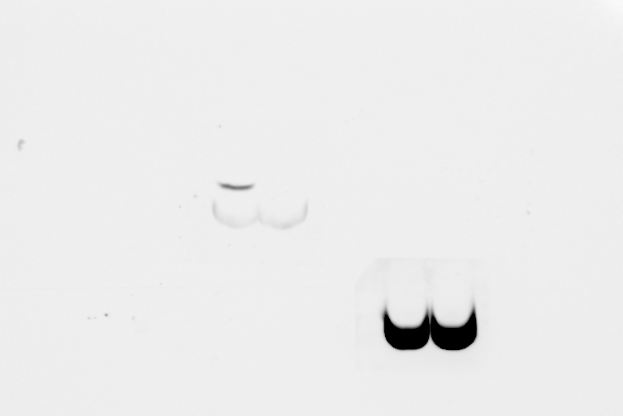


- +
- +

ATG4B sgRNA

Screamble

β-actin


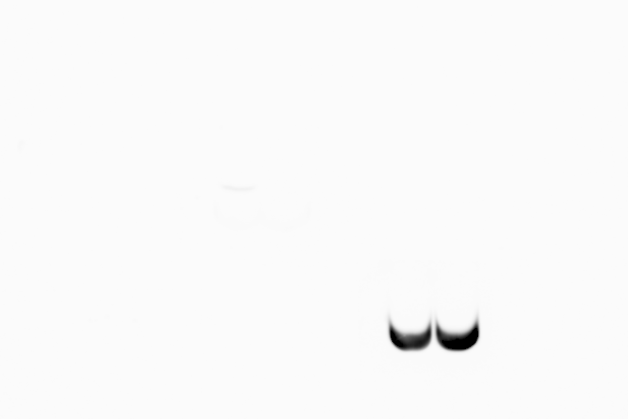


ATG4B sgRNA

Screamble

- +
- +

Figure 4H

SQSTM1_(n1)


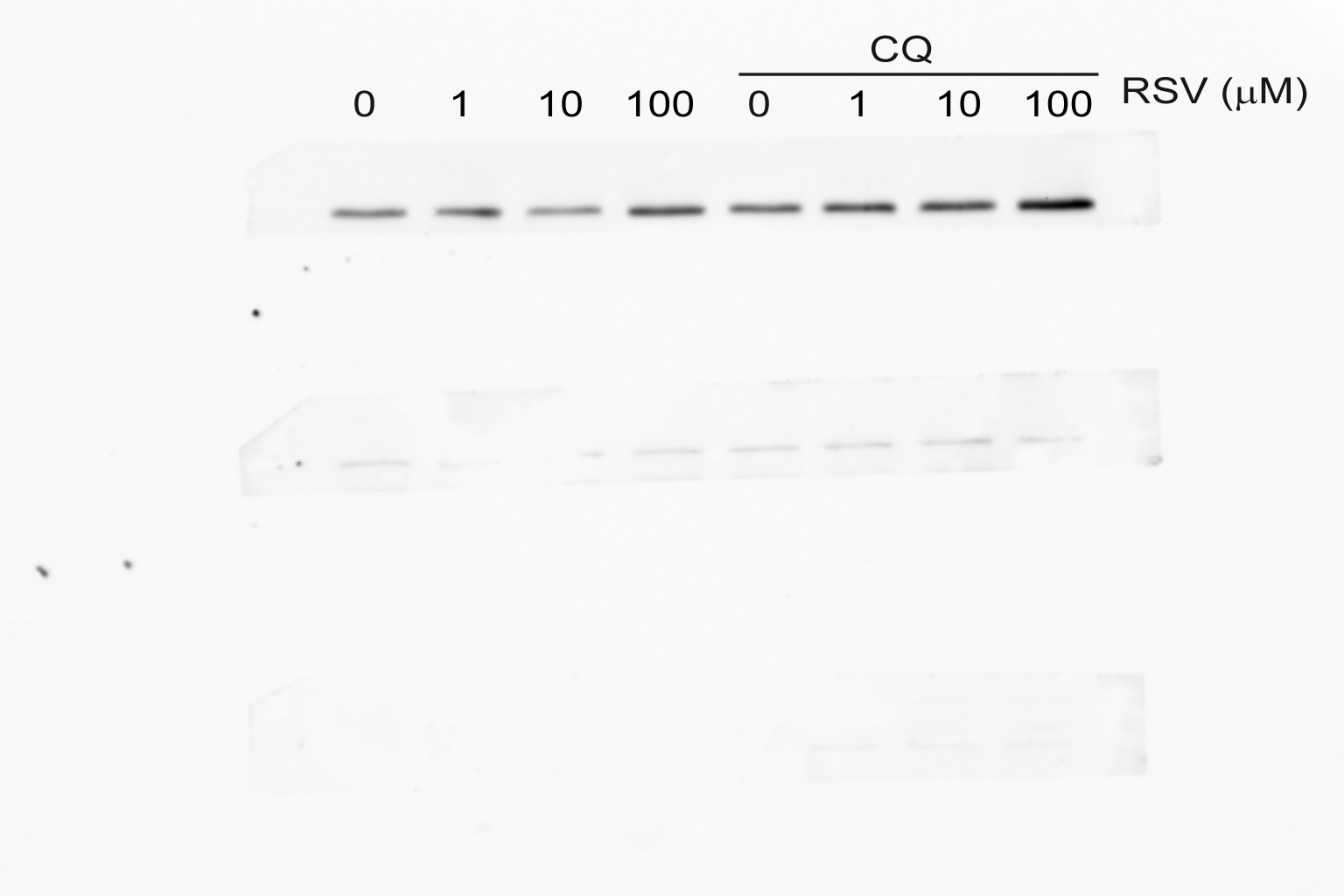


LC3_(n1)


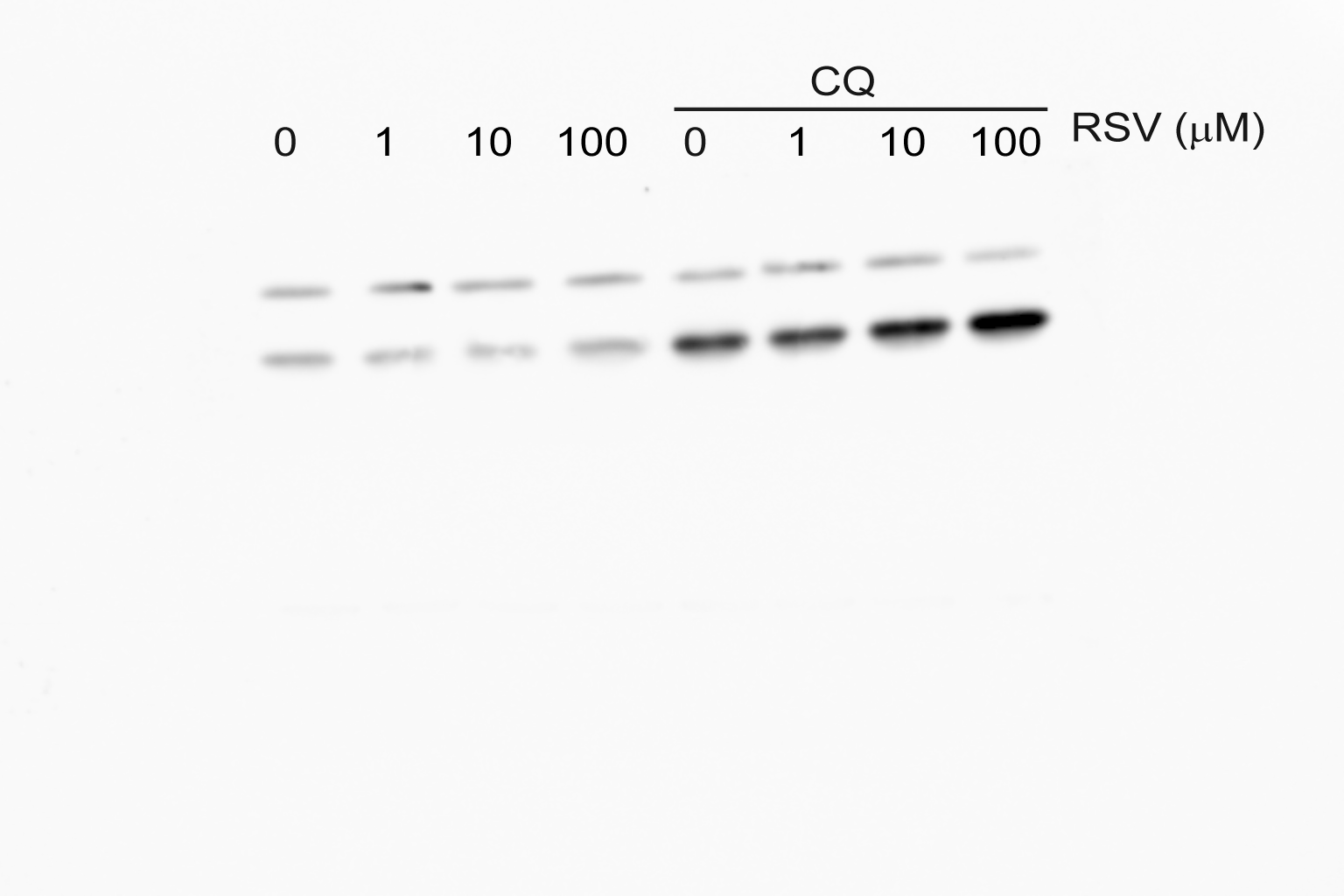


β-actin_(n1)


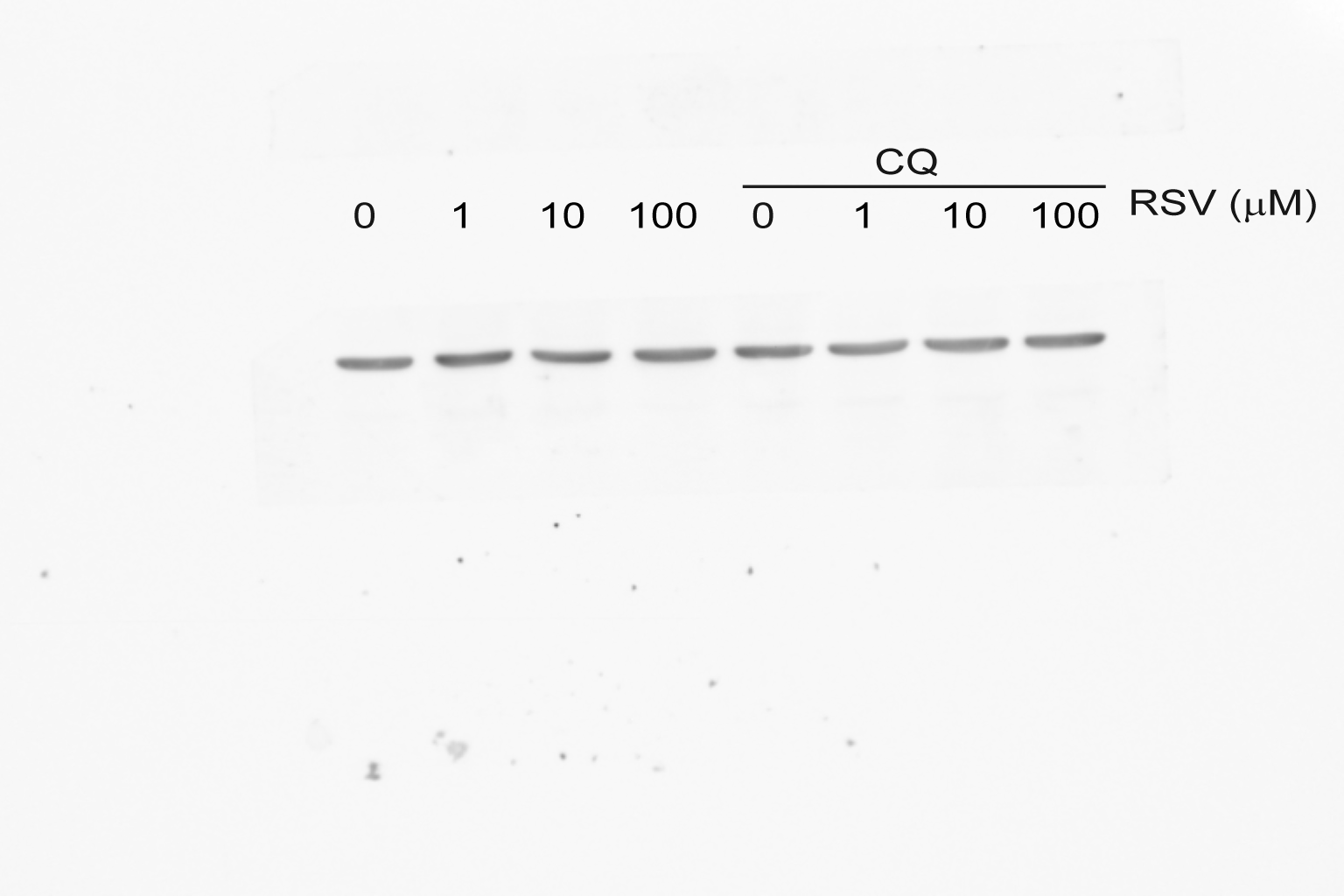


SQSTM1_(n2)


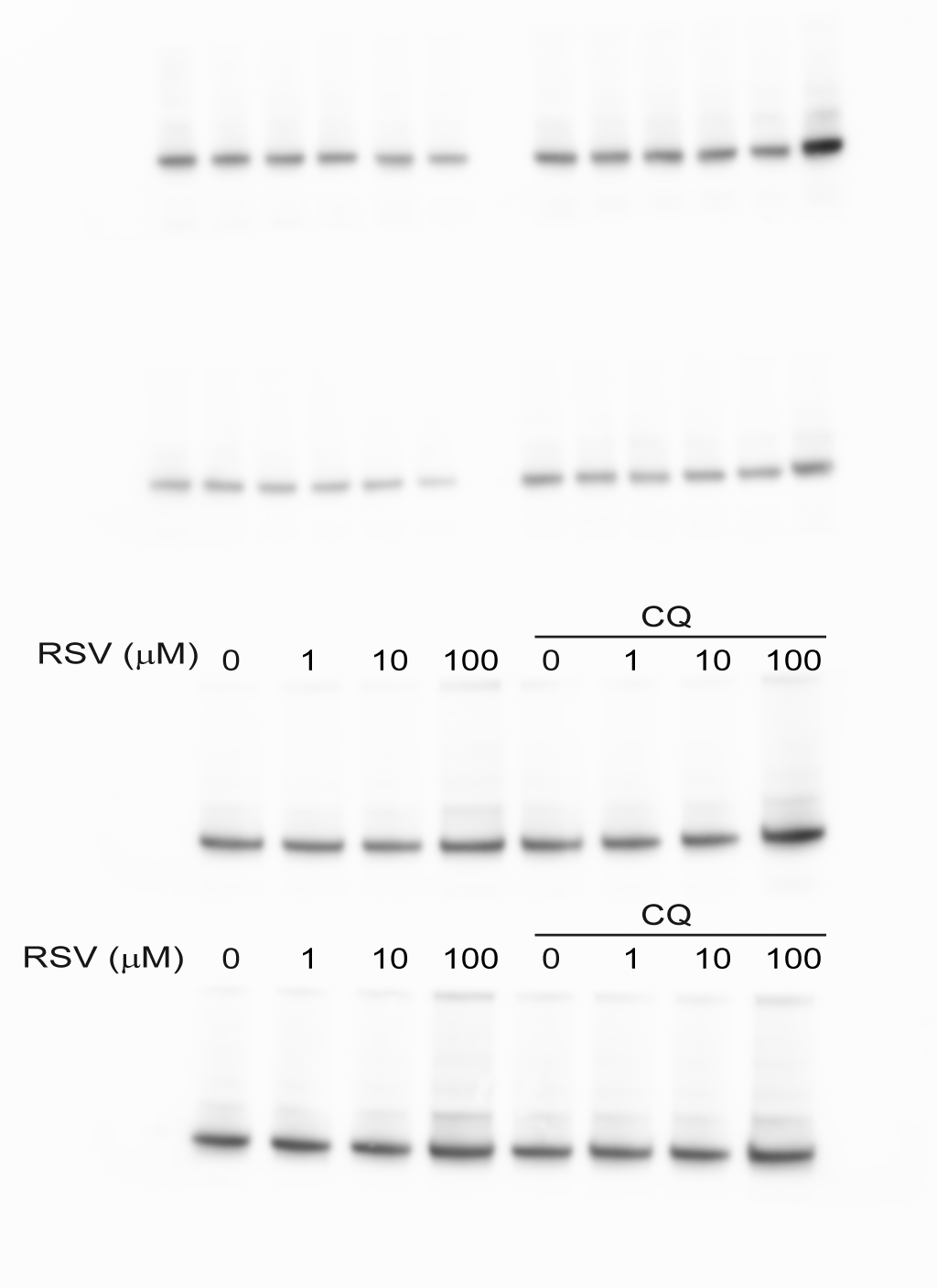


LC3_(n2)


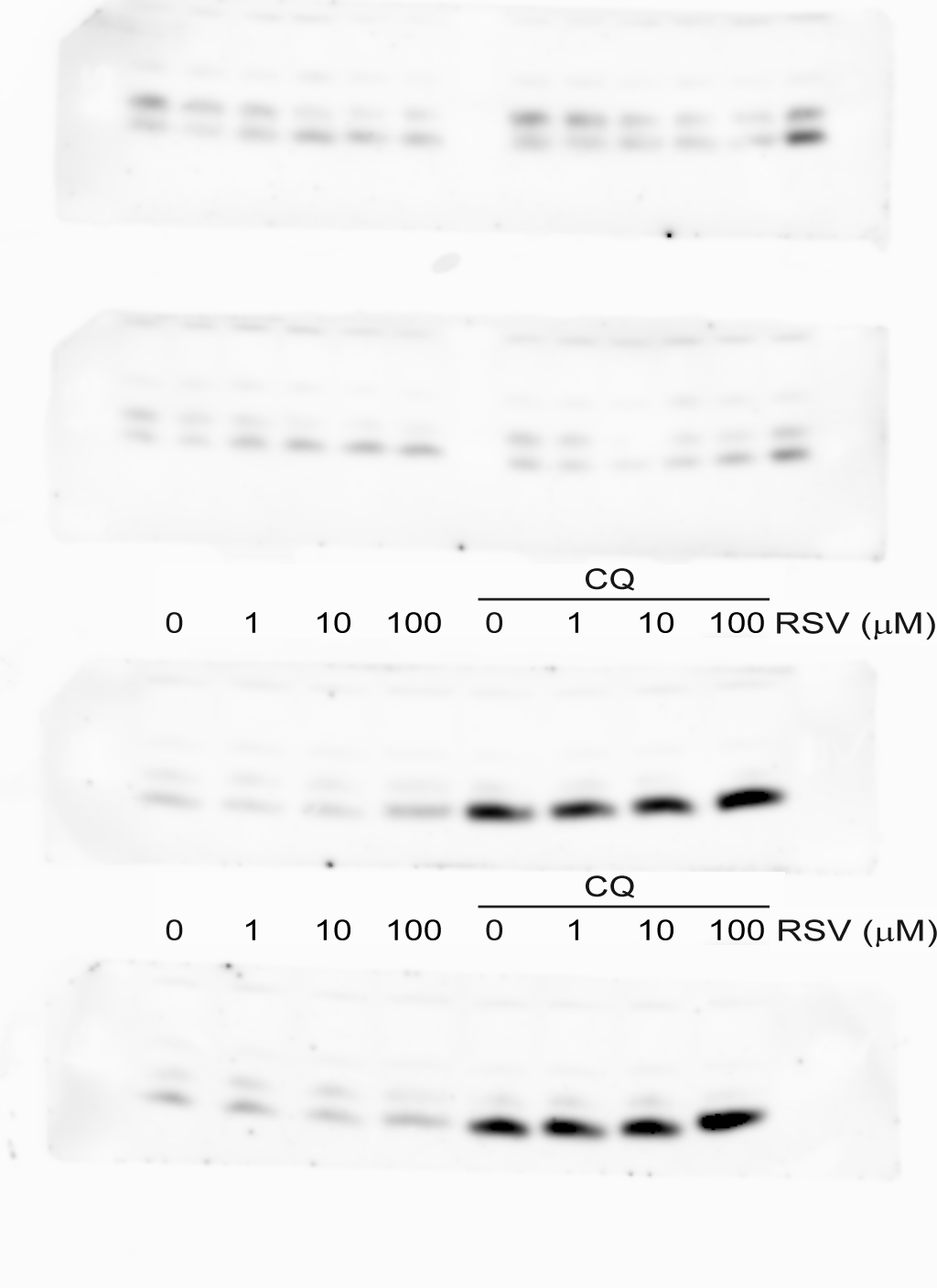


β-actin_(n2)


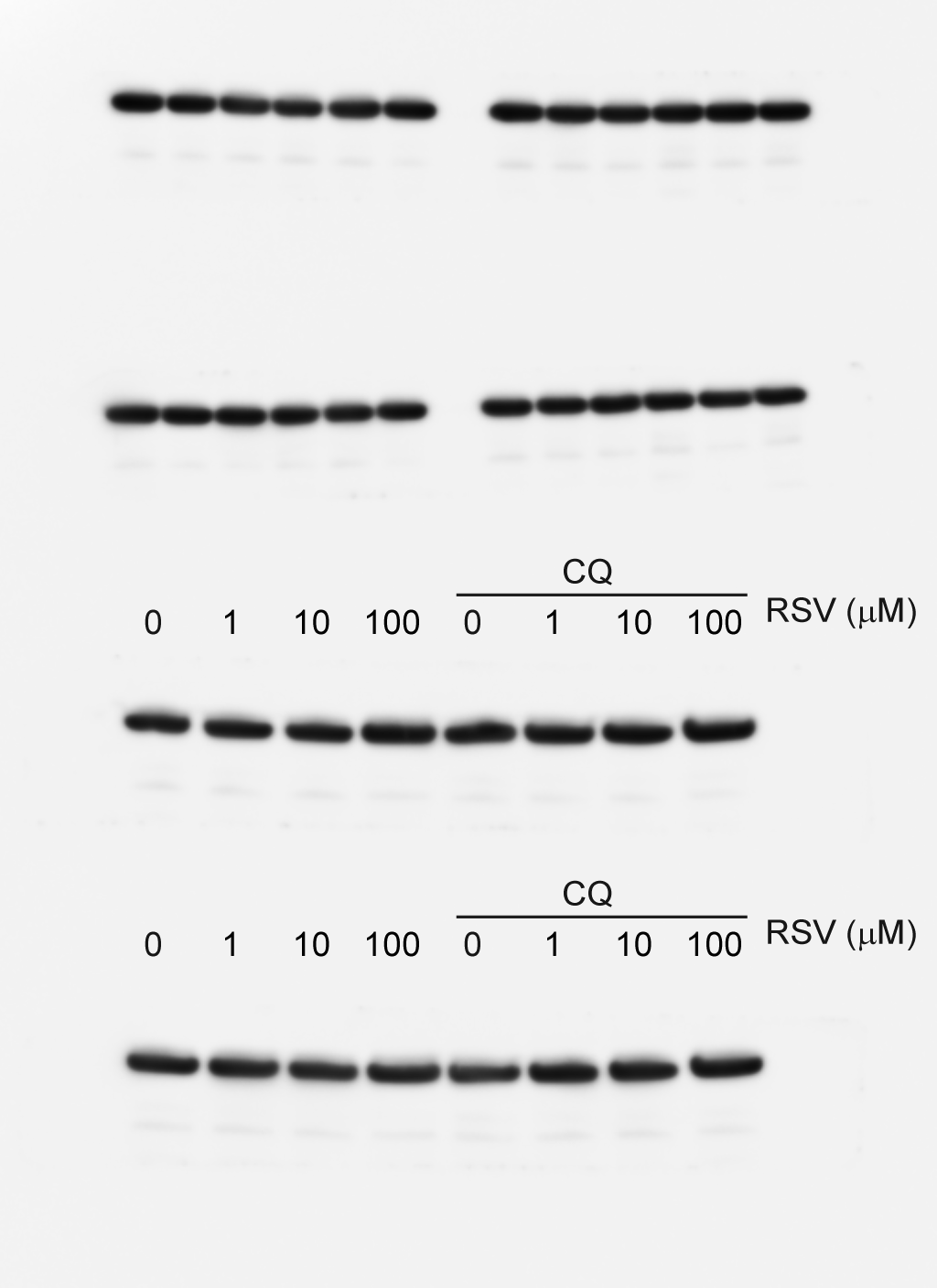


SQSTM1_(n3)


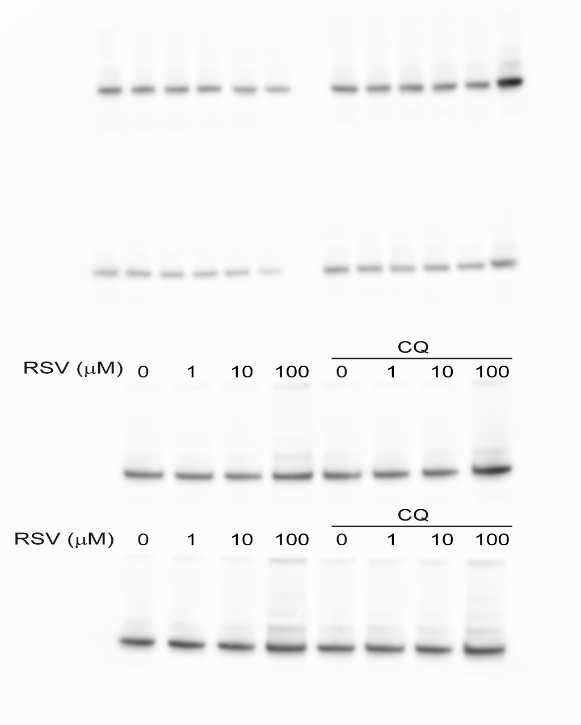


LC3_(n3)


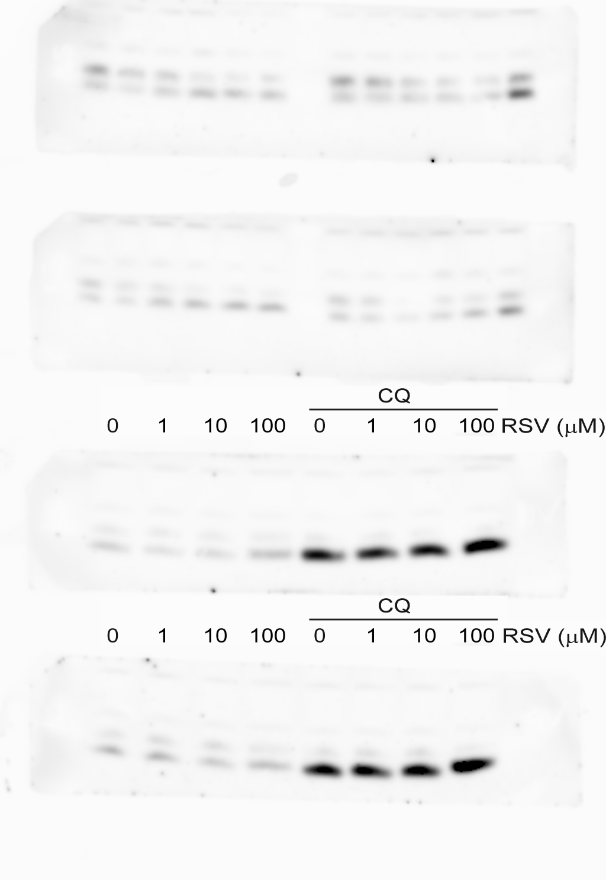


β-actin_(n3)


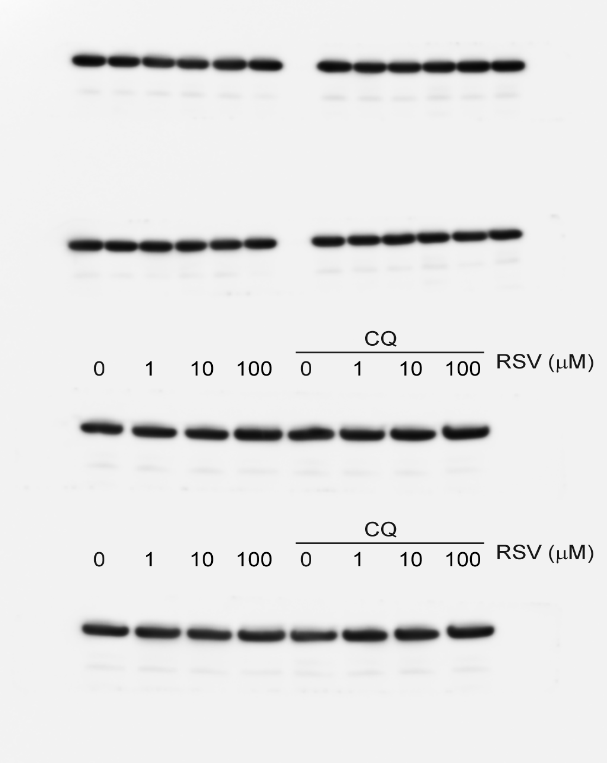


Figure 5D

Caspase-3_(n1)


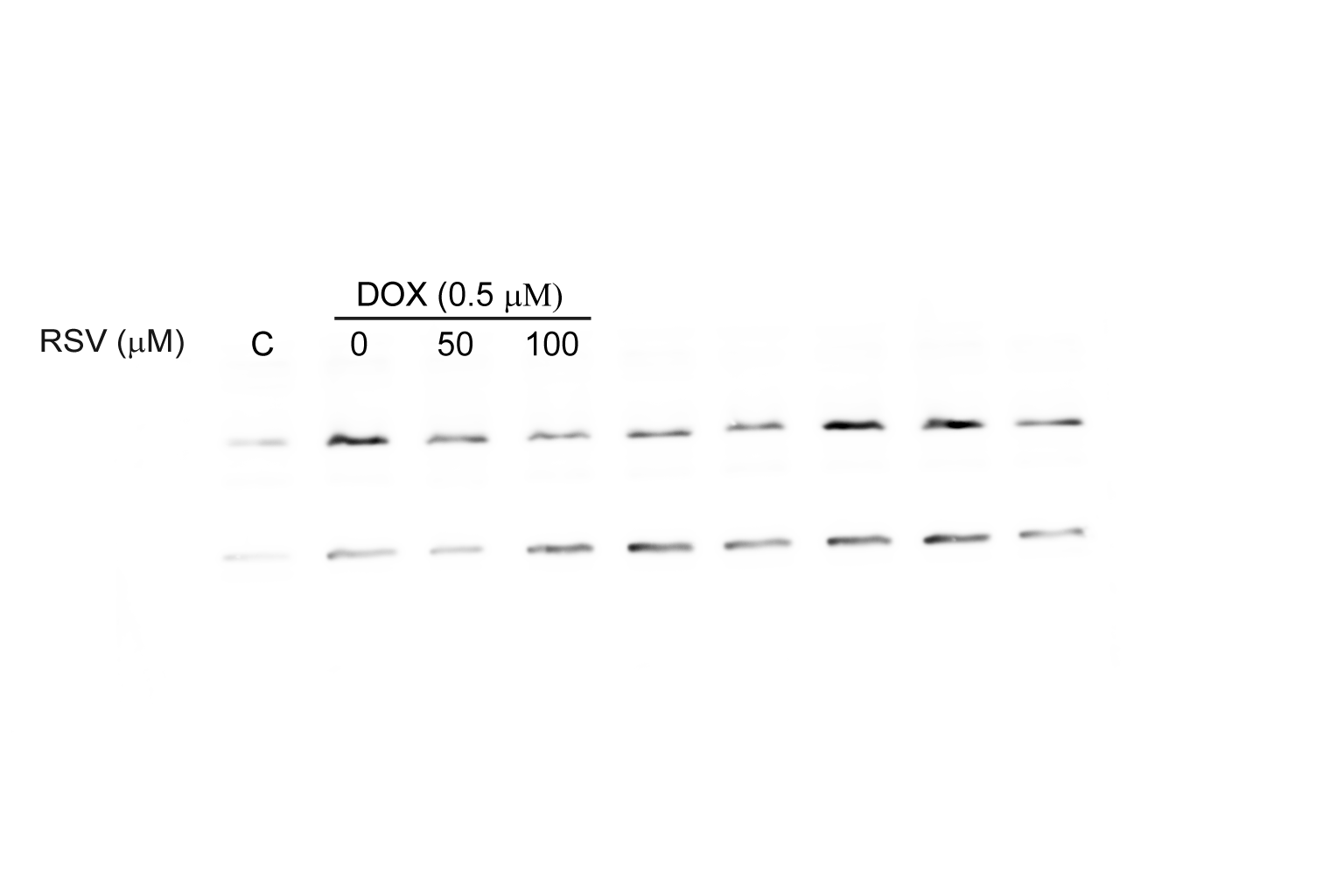


Cleaved-PARP_(n1)


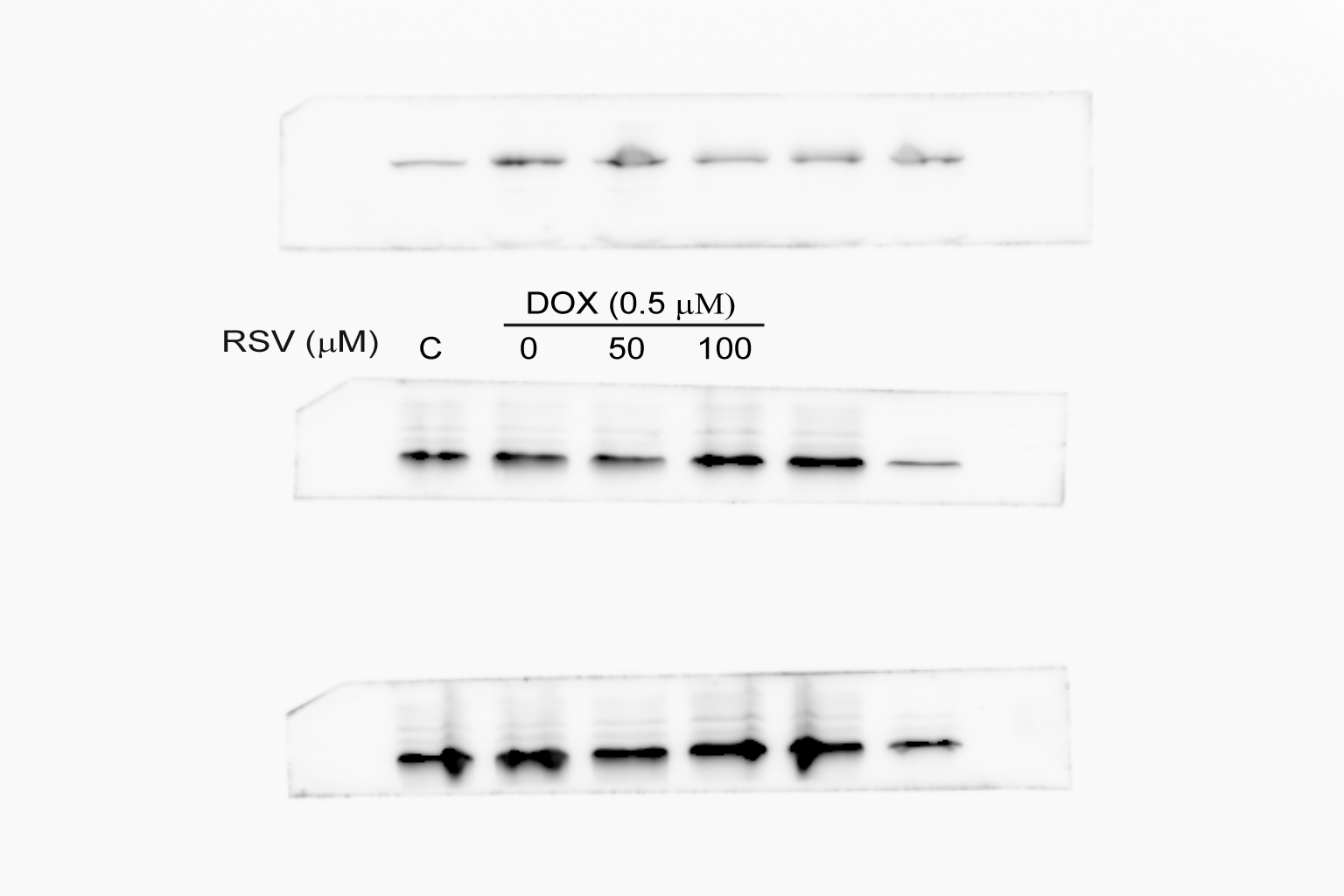


β-actin_(n1)


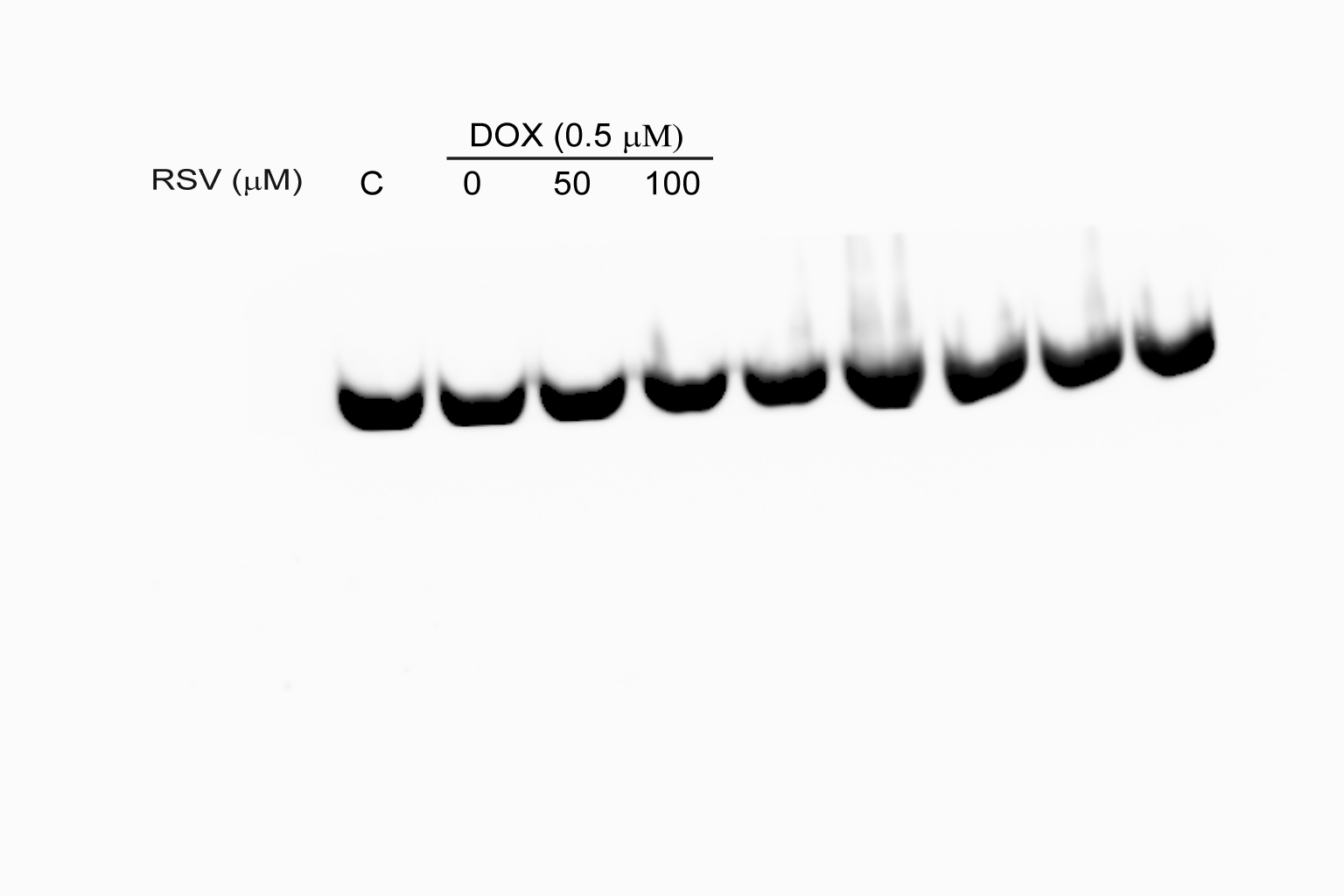


Caspase-3_(n2)


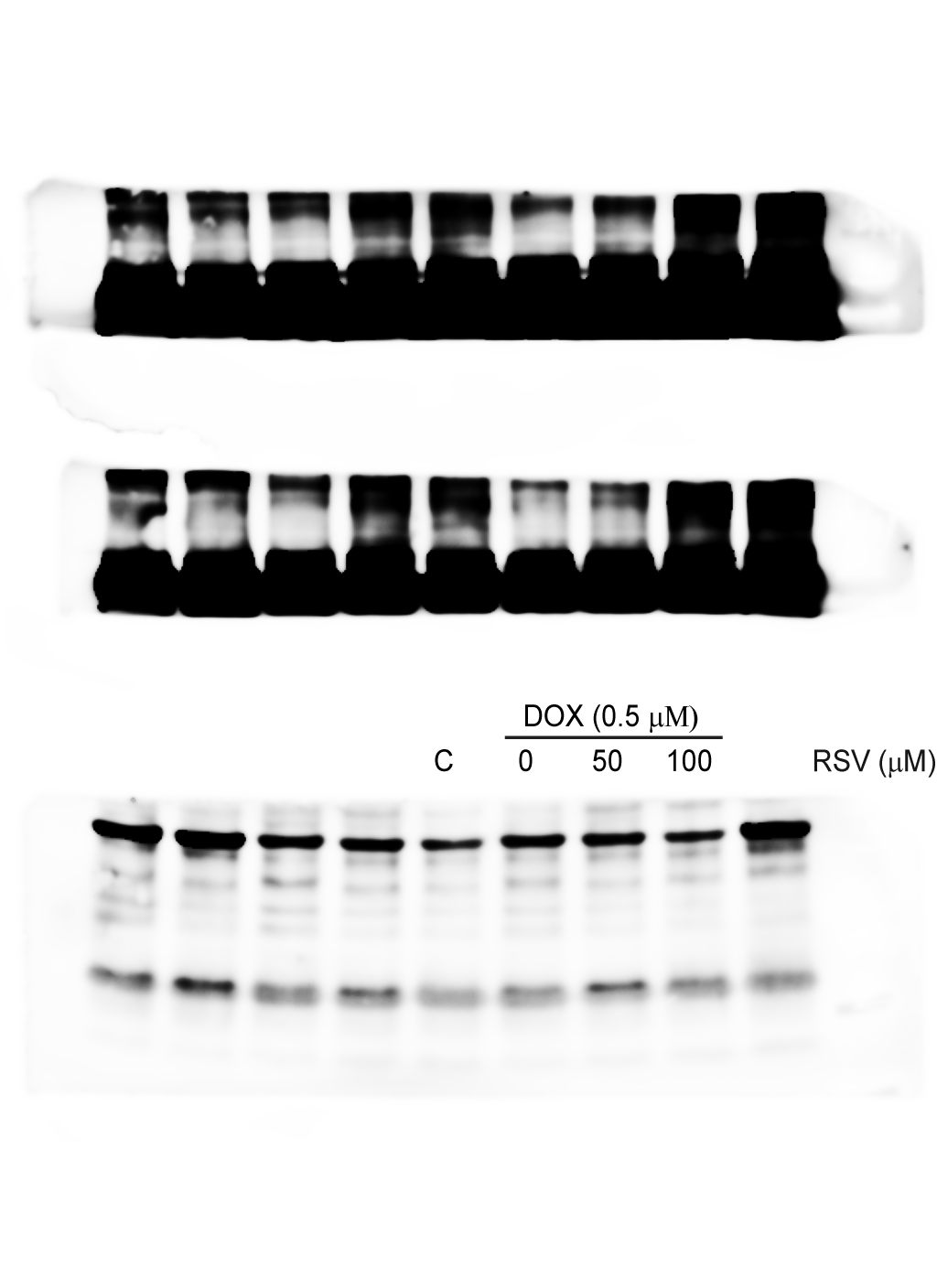


Cleaved-PARP_(n2)


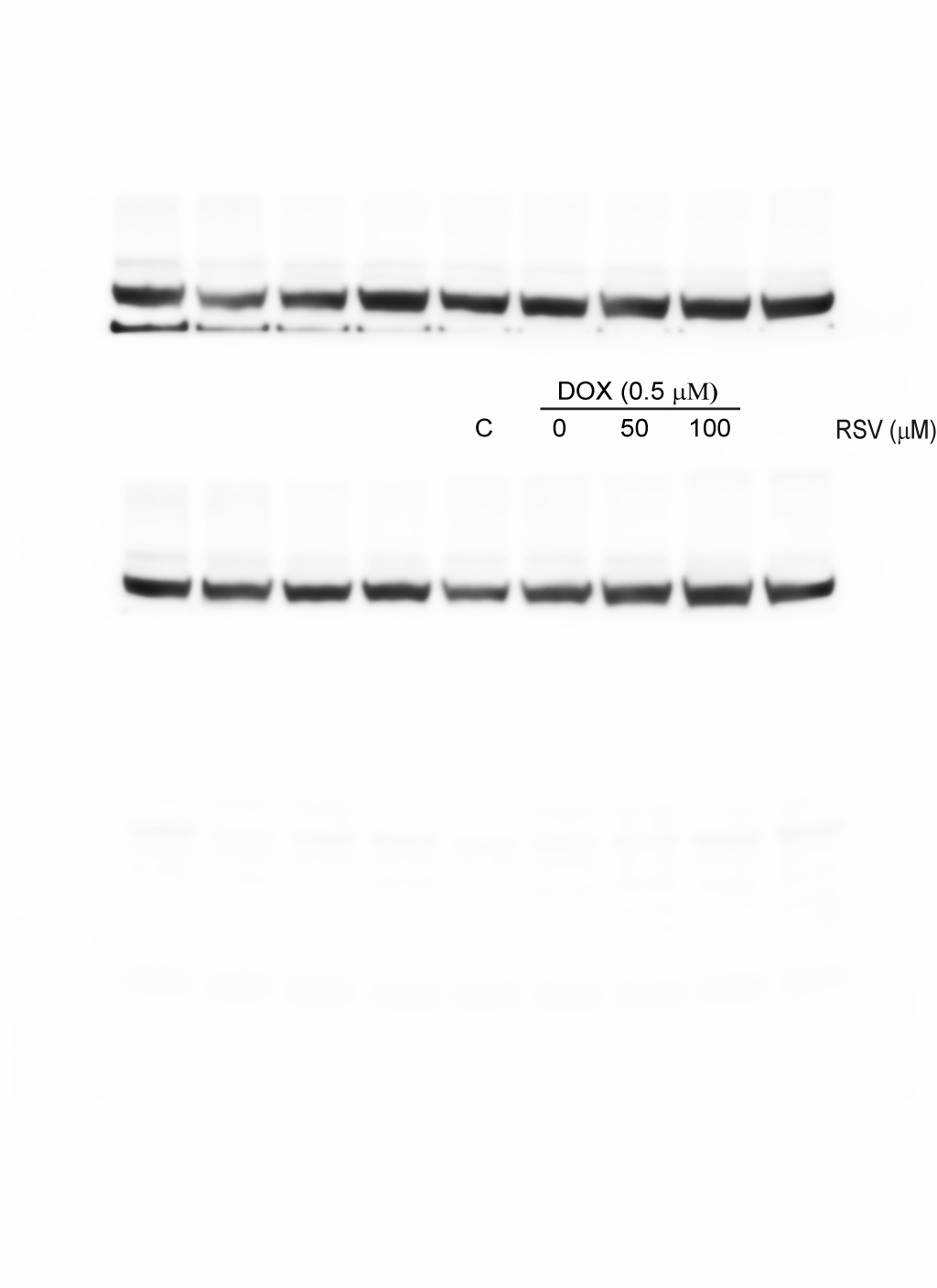


β-actin_(n2)


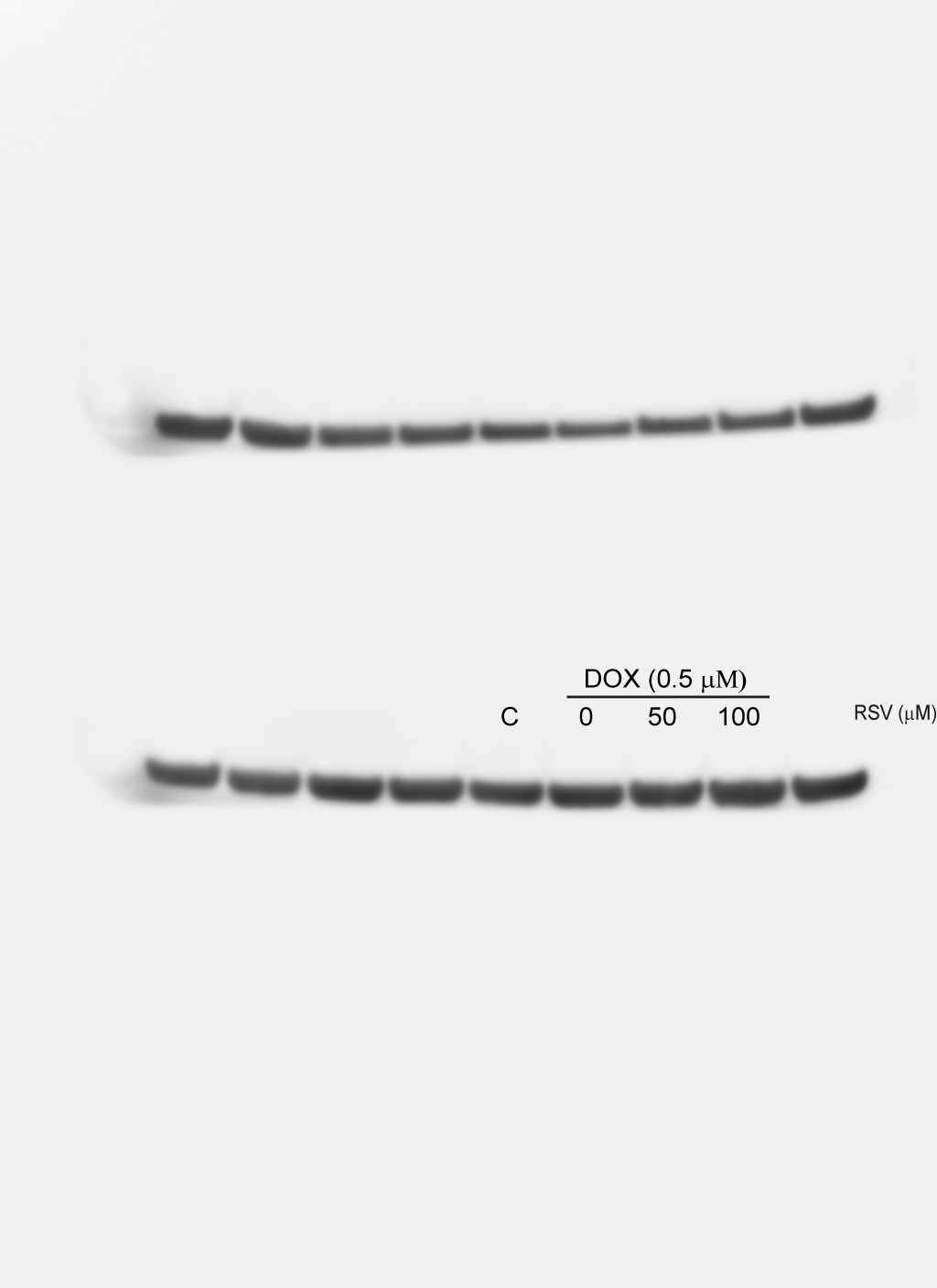


Caspase-3_(n3)


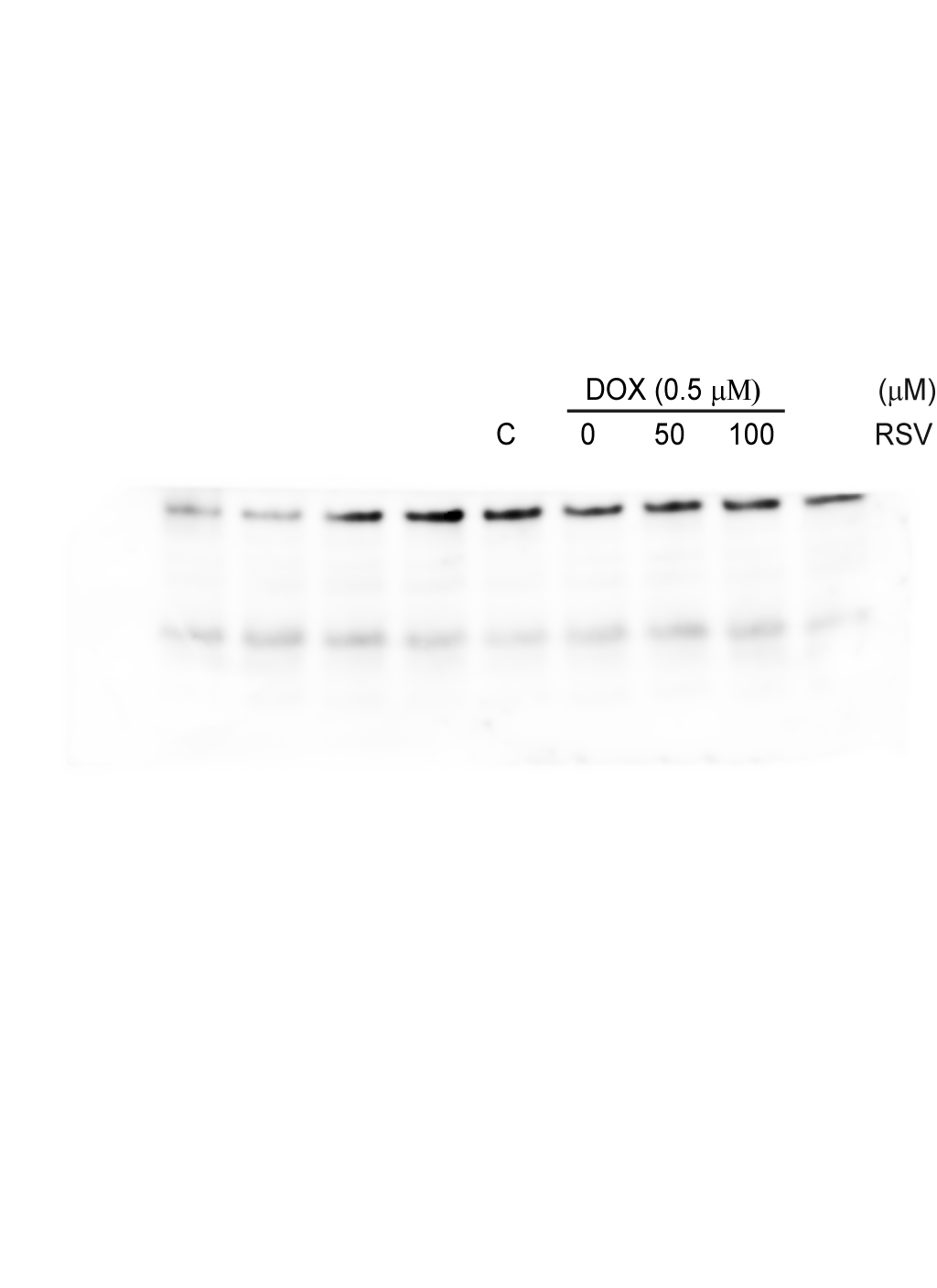


Cleaved-PARP_(n3)


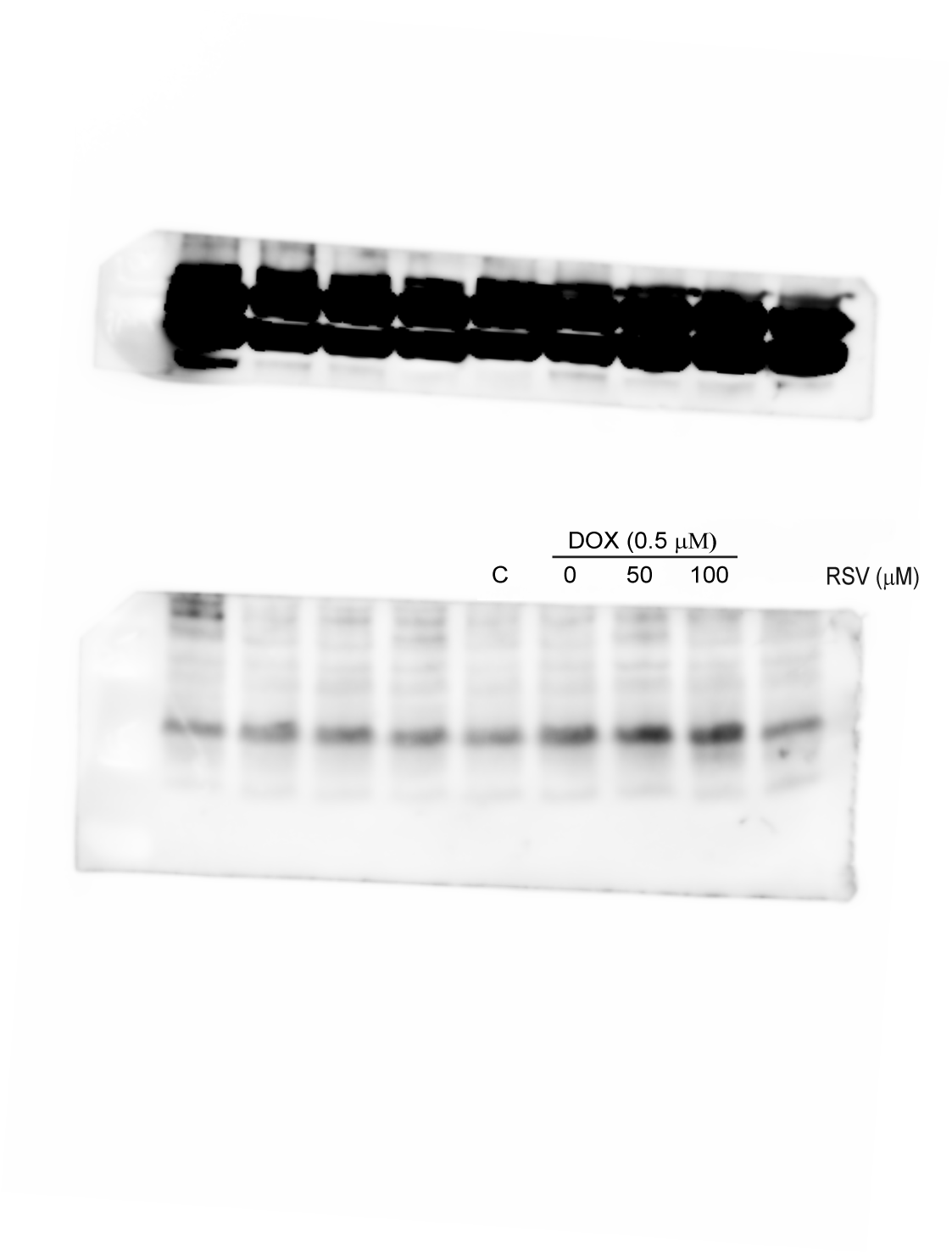


β-actin_(n3)


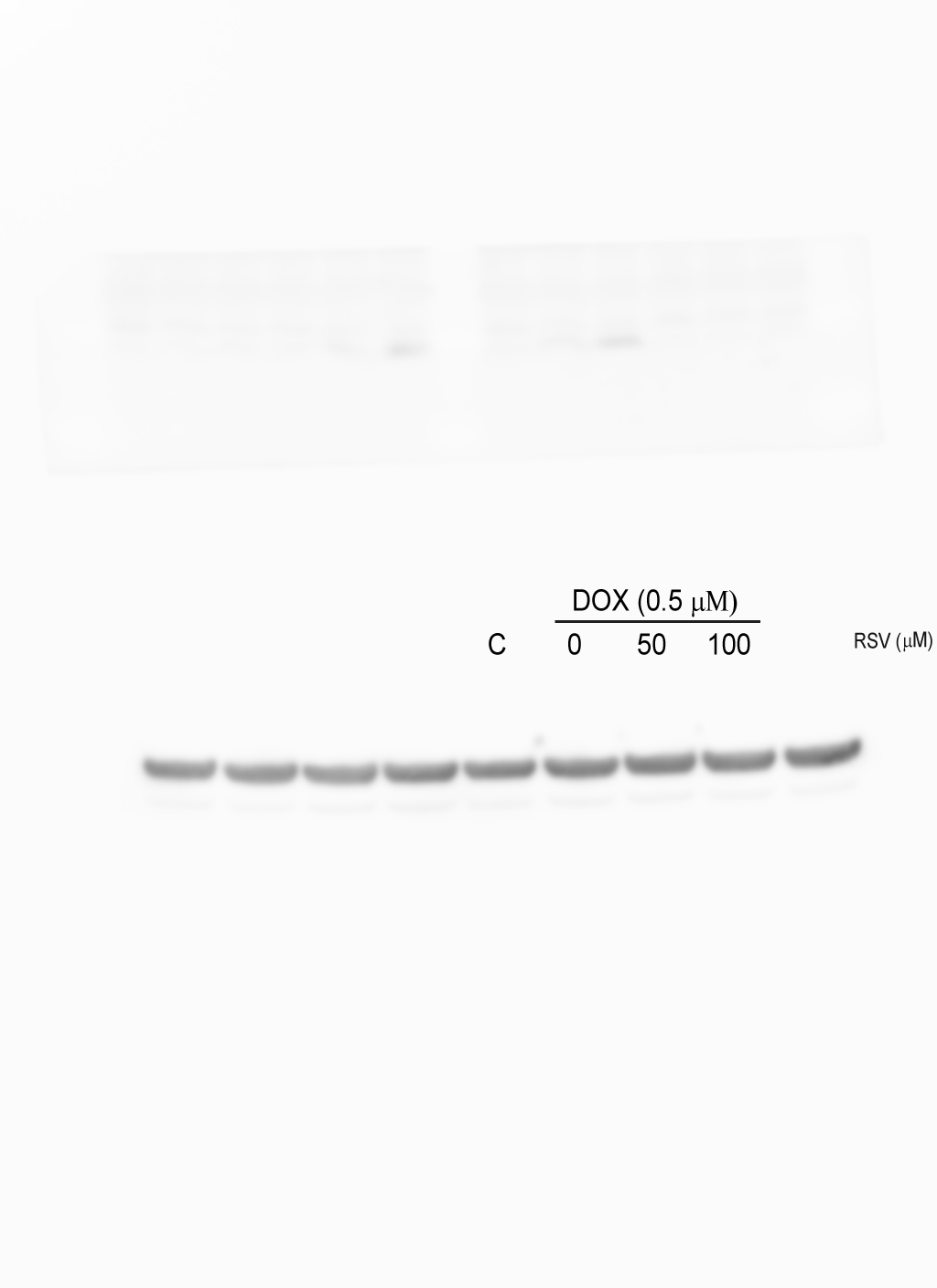


Caspase-3_(n4)


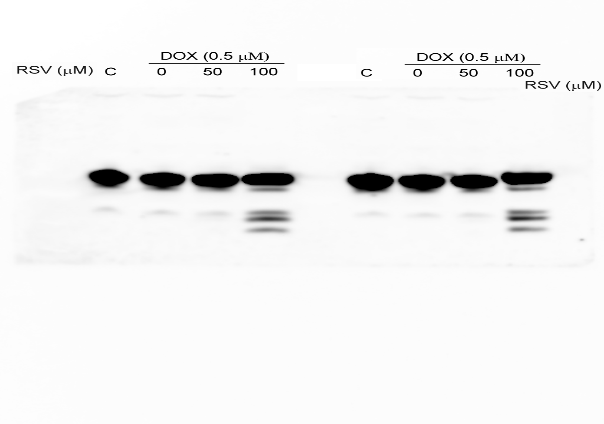


Cleaved-PARP_(n4)


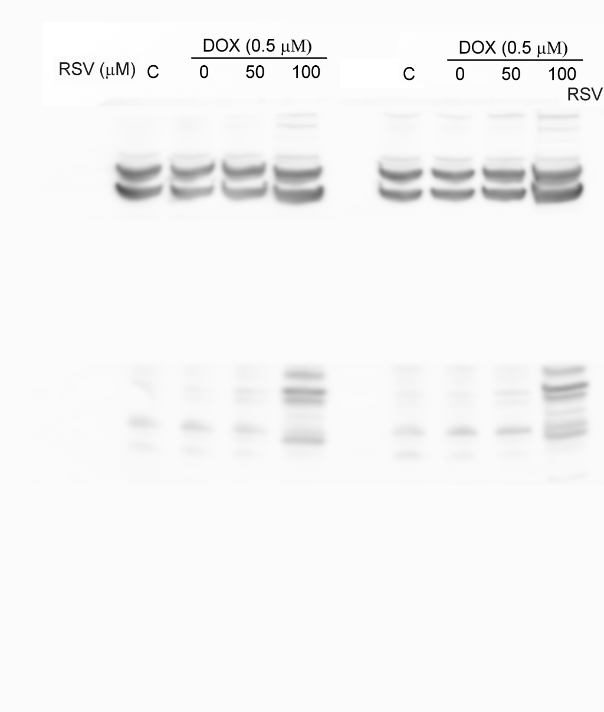


β-actin_(n4)


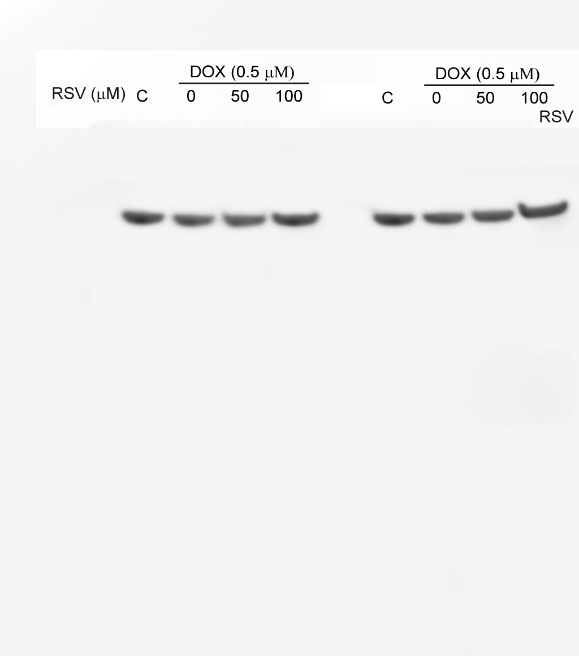


Supplementary Figure 6

Monesin

Luciferase-Full length_(n1)


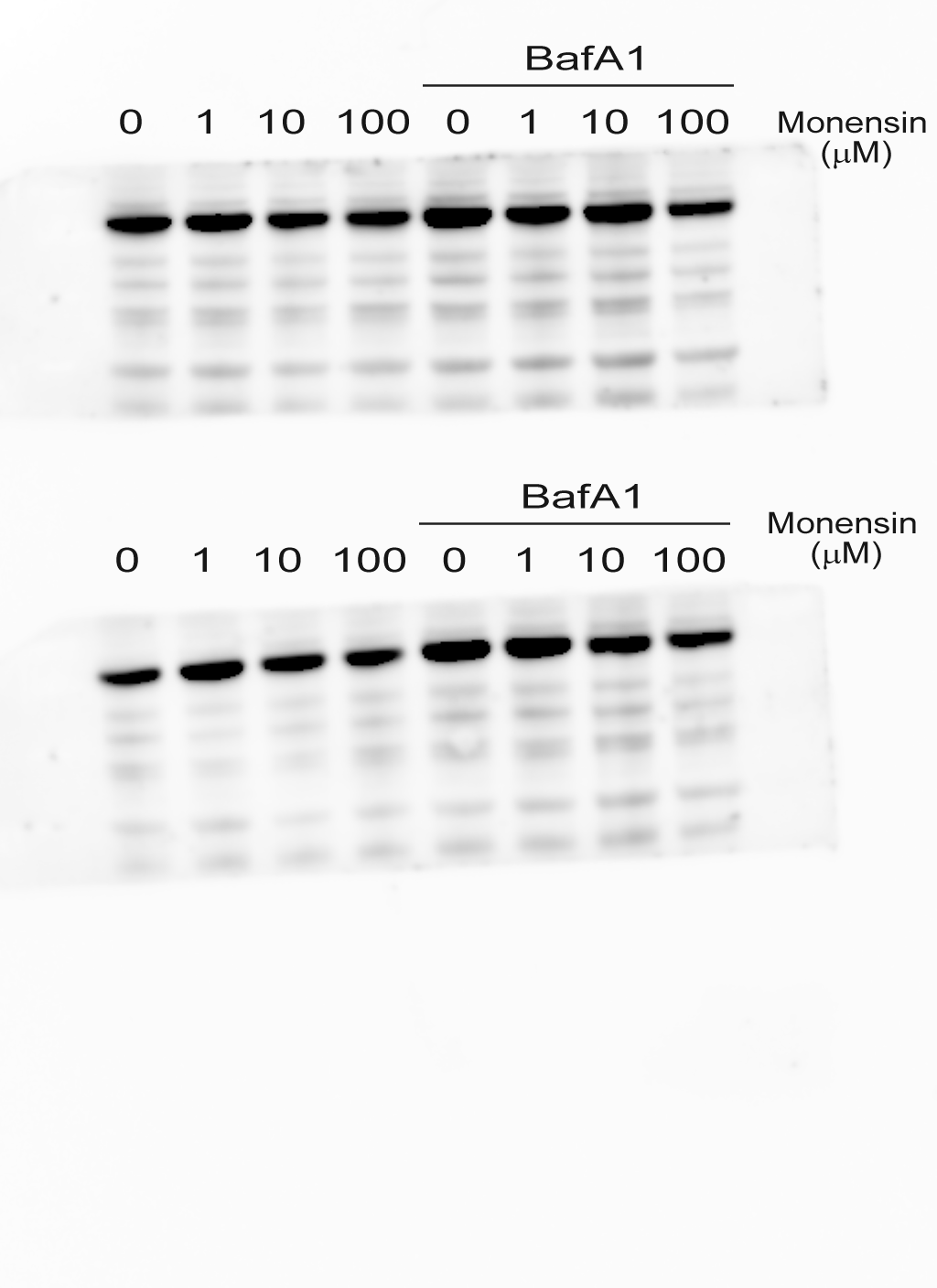


Luciferase-Cleavage_(n1)


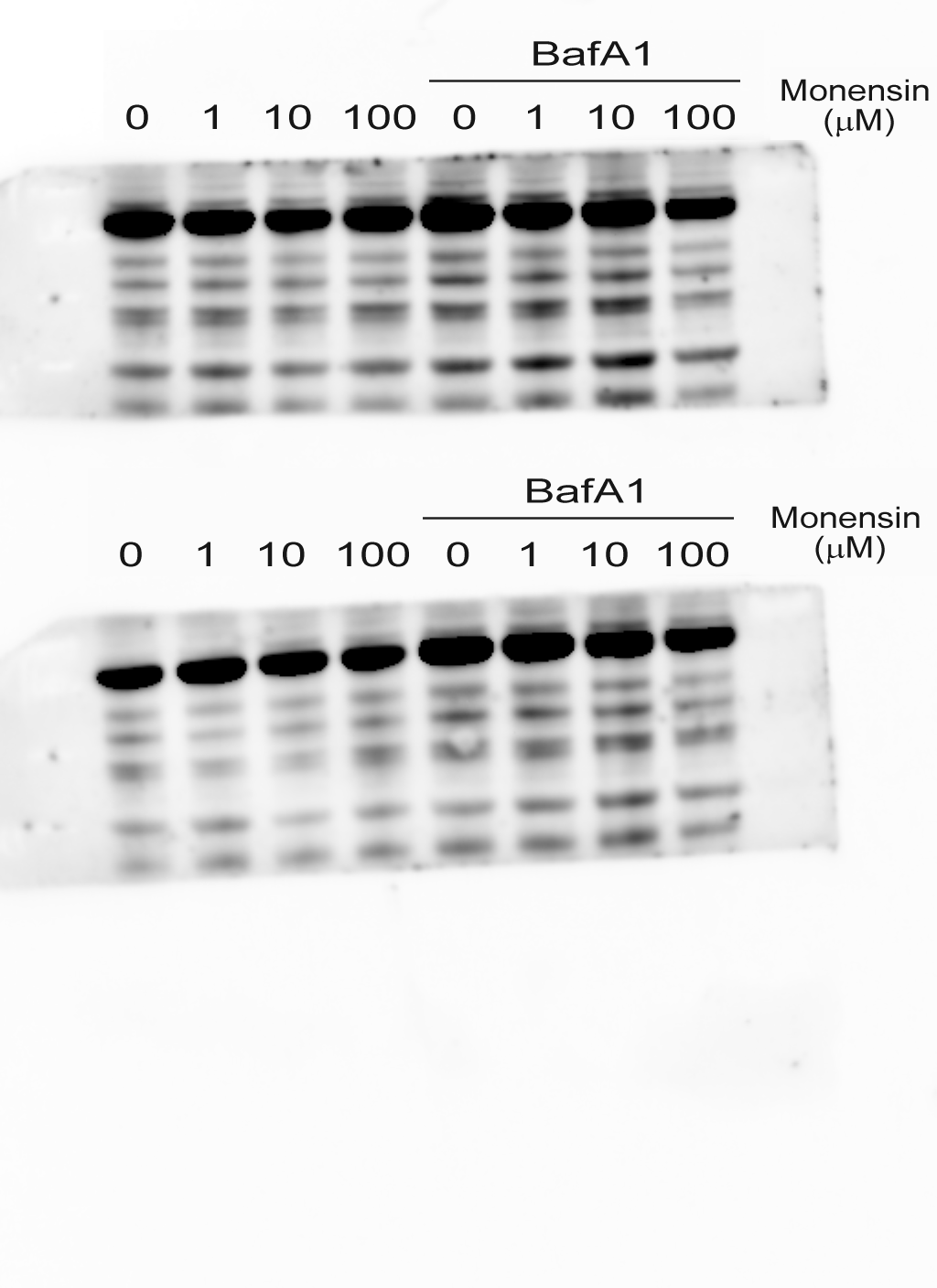


LC3_(n1)

β-actin_(n1)

Luciferase-Full length_(n2)

Luciferase-Cleavage_(n2)

LC3_(n2)

β-actin_(n2)

Luciferase-Full length_(n3)

Luciferase-Cleavage_(n3)

LC3_(n3)

β-actin _(n3)
